# Synthesis and Biological Evaluation of Thiophene-Bridged Indole–Artesunate Hybrids as Selective Anticancer Agents

**DOI:** 10.1021/acsomega.6c04482

**Published:** 2026-07-24

**Authors:** Emrah Kavak, Doğan Çetin, Metin Konuş, Arif Kivrak

**Affiliations:** † 226846 Necmettin Erbakan University, BITAM-Science and Technology Research and Application Center, Konya 42090, Turkey; ‡ Molecular Biology and Genetics, Faculty of Sciences, 53000 Van Yüzüncü Yil University, Van 65080, Turkey; § Chemistry, Faculty of Science, 53004 Eşkisehir Osmangazi University, Eskişehir 26040, Turkey

## Abstract

In this study, five thiophene-bridged indole–artesunate hybrid molecules were synthesized and evaluated as anticancer candidates. Antioxidant screening by DPPH, ABTS, galvinoxyl, and FRAP methods showed no measurable radical scavenging or reducing activity, indicating that these compounds should not be described as antioxidants. Cytotoxic effects were evaluated in Ishikawa, HepG2, HT-29, MDA-MB-231, and Panc-1 cancer cell lines using the MTT assay, and compounds (3*R*,5*aS*,6*R*,8*aS*,9*R*,12*R*,12*aR*)-3,6,9-trimethyldecahydro-12*H*-3,12-epoxy­[1,2]­dioxepino­[4,3-*i*]­isochromen-10-yl 4-(5-(1-methyl-2-phenyl-1*H*-indol-3-yl)­thiophen-2-yl)-4-oxobutanoate (**D1**), synthesis of (3*R*,5*aS*,6*R*,8*aS*,9*R*,12*R*,12*aR*)-3,6,9-trimethyldecahydro-12*H*-3,12-epoxy­[1,2]­dioxepino­[4,3-*i*] isochromen-10-yl 4-(5-(1-methyl-2-pentyl-1*H*-indol-3-yl)­thiophen-2-yl)-4-oxobutanoate (**D2**), and synthesis of (3*R*,5*aS*,6*R*,8*aS*,9*R*,12*R*,12*aR*)-3,6,9-trimethyldecahydro-12*H*-3,12-epoxy­[1,2]­dioxepino­[4,3-*i*]­isochromen-10-yl 4-(5-(1-methyl-2-(*p*-tolyl)-1*H*-indol-3-yl)­thiophen-2-yl)-4-oxobutanoate (**D3**) were found to exhibit significant cytotoxic activity. Compound **D1** (EC_50_: 20.22 μM) showed activity similar to paclitaxel (EC_50_: 19.3 μM) in Ishikawa cells, while compounds **D1** and **D2** (EC_50_: 5.43 μM, EC_50_: 5.63 μM) showed comparable activity to the positive control (EC_50_: 4.5 μM) in HepG2 cells; compound **D3** (EC_50_: 2.75 μM) showed a higher cytotoxic effect. No measurable cytotoxicity was observed in the healthy human endothelial (HUVEC) cell line for any of the tested compounds. Invasion assay showed that compounds **D1** and **D2** strongly inhibited cell invasion, particularly in HepG2 and Panc-1 cell lines. Annexin V/PI assays and real-time PCR results showed that the compounds induced apoptosis, increasing the expression of BAX, P53, and caspase genes while suppressing Bcl-2 expression. Overall, the findings indicate that although the compounds studied in this work do not possess antioxidant properties, they exhibit selective cytotoxic, anti-invasive, and proapoptotic effects, warranting further investigation as potential anticancer agent candidates.

## Graphical abstract

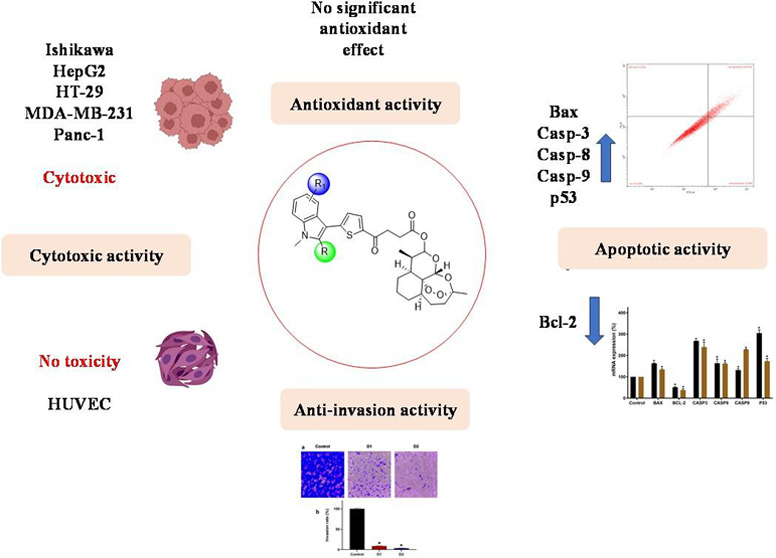

## Introduction

Artemisinin is isolated from the *Artemisia annua* plant and is identified by its endoperoxide architecture with a lactone moiety. Semisynthetic derivatives of artemisinin, dihydroartemisinin, artesunate, and artemether have been synthesized to increase the pharmacological activity and the pharmacokinetic profile of the parent drug derivatives.^1^ As a natural product, artemisinin and artemisinin-based combination therapies have contributed to the reduction of malaria^2^ and cancer cell lines.^3,4^ One of the artemisinin derivatives, artesunate, was approved by the Food and Drug Administration (FDA) as an antimalarial drug on May 26, 2020.^5^ Furthermore, artesunate derivatives have been reported to have anticancer,^6^ anti-inflammatory,^7^ antiangiogenic,^8^ proapoptotic,^9^ antimicrobial,^10^ antiviral^11^ effects, and reactive oxygen species (ROS)-modulating activities.^12^

Recent studies have highlighted molecular hybridization as an effective strategy for improving the pharmacological profiles of artemisinin/artesunate-derived anticancer candidates by combining complementary bioactive scaffolds within a single molecular framework.^13^ Furthermore, artesunate-containing hybrids have been reported to exert anticancer effects through ROS generation, mitochondrial dysfunction, apoptosis induction, and other multitarget mechanisms.^14^

Indole is a heterocyclic molecule formed by a combination of a benzene ring and a pyrrole ring found in both natural and manufactured substances.^15^ Indole derivatives have been reported to possess numerous biological properties, including antihypertensive,^16^ anti-inflammatory,^17^ antimicrobial,^18^ antiproliferative,^19^ proapoptotic,^20^ and anticancer.^19–22^ Indole and thiophene moieties remain attractive pharmacophores in medicinal chemistry because of their diverse biological activities including anticancer effects. Recent studies have demonstrated that hybrid molecules incorporating these heterocyclic scaffolds may exhibit enhanced antiproliferative and proapoptotic activities against various cancer cell lines.^23,24^

Nowadays, the synthesis of hybrid derivatives of bioactive natural substances has become a useful strategy to increase biological and pharmacokinetic activities.^1,25–28^

In this study, we synthesized indole–thiophene-based scaffolds designed using molecular hybridization principles, with particular focus on anticancer applications. The choice of indole is supported by its widely known biological activity, including antiproliferative,^29^ and cytotoxic effects across multiple cancer cell lines.^30^ Based on previous reports on artesunate derivatives, ROS-associated apoptotic mechanisms may contribute to the biological activity of this scaffold.^24^

By uniting these two pharmacophores into a single structure, the goal is to develop compounds capable of multitarget interaction. The hybrid molecules (**D1**, **D2**, **D3**, **D4**, and **D5**) are hypothesized to act through complementary pathways, including oxidative stress induction (from artesunate) and signal modulation or DNA intercalation (from indole), potentially leading to enhanced efficacy and reduced drug resistance.^31,32^ However, because ROS production, mitochondrial membrane potential, cytochrome *c* release, and direct target interactions were not experimentally evaluated in the present study, these mechanisms are considered only as possible hypotheses rather than confirmed pathways for the synthesized compounds.

In the pursuit of novel anticancer agents through molecular hybrid, a new one-pot synthetic strategy was developed for the construction of indole-based hybrid molecules. The aim was to integrate biologically active scaffolds into a single molecular framework to exploit their synergistic therapeutic potential. Five novel indole–artesunate hybrid compounds were designed and synthesized, and their biological and pharmacological properties were investigated. Four distinct assays (DPPH, ABTS, galvinoxyl, and FRAP) were used to investigate the compounds’ antioxidant capacity. Several cancer cell lines (Panc-1, MDA-MB-231, HT-29, HepG2, and Ishikawa) and a healthy cell line (HUVEC) were employed for investigating the compounds’ cytotoxic impacts. In addition, the invasive capacity of these compounds on cancer cells, their apoptotic effects, and changes in mRNA expression of genes implicated in the apoptotic pathway were studied.

## Results and Discussion

### Chemistry

To synthesize indole-containing artesunate analogues, based on retrosynthetic analysis (Figure 1), first we synthesized 2-aryl/alkyl-3-(2-thienyl)­indole derivatives via a one-pot Sonogashira coupling and intramolecular cyclization sequence **1­(a-d)**. The reaction started with the coupling of 2-iodo-*N*,*N*-dimethylaniline with various aryl/alkyl acetylenes in the presence of palladium and copper catalysts, affording *N*,*N*-dimethyl-2-ethynyl­(aryl/alkyl)­aniline (**2a-e**) intermediates with MW. Without isolating these intermediates, 2-iodothiophene and acetonitrile were added directly to the reaction vessel. The reaction was subsequently treated with microwave irradiation, enhancing cyclization to afford one-pot indole derivatives **3­(a-e)**. The overall efficiency of the one-pot procedure was demonstrated through good to excellent yields. The *p*-tolyl-substituted derivative (compound **3c**) was isolated in the highest yield (99%), followed by compounds **3a** (72%), **3b** (70%), and **3d** (74%). To introduce additional functional diversity, the one-pot indole products were subjected to Friedel–Crafts-type regioselective acylation reaction with succinic anhydride in the presence of AlCl_3_, which selectively introduced a ketone-bridged carboxylic acid moiety at the 5-position of the thiophene ring **4­(a-e)**. This transformation yielded carboxylic acid-functionalized indole molecules. Overall, carboxylic acid-functionalized indole and DHA the among the modified derivatives, the highest yield was observed for the C-5 substituted indole **D4** (77%), 2-phenyl **D1** (71%), aliphatic 2-heptyl-substituted compound **D2** (72%), 2-*p*-tolyl-substituted **D3** (26%), and 2-*p*-methoxyphenyl **D5** (52%) (Figure 2).

**Figure 1:**
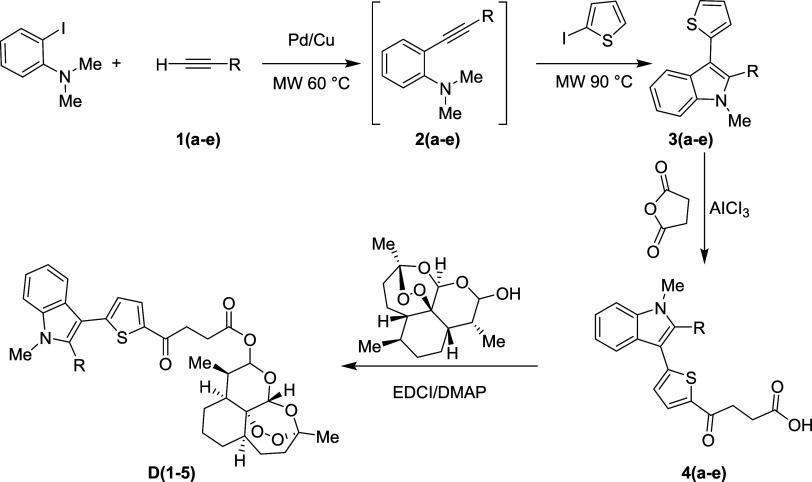
General explanation of the Artemisinin derivatives.

**Figure 2:**
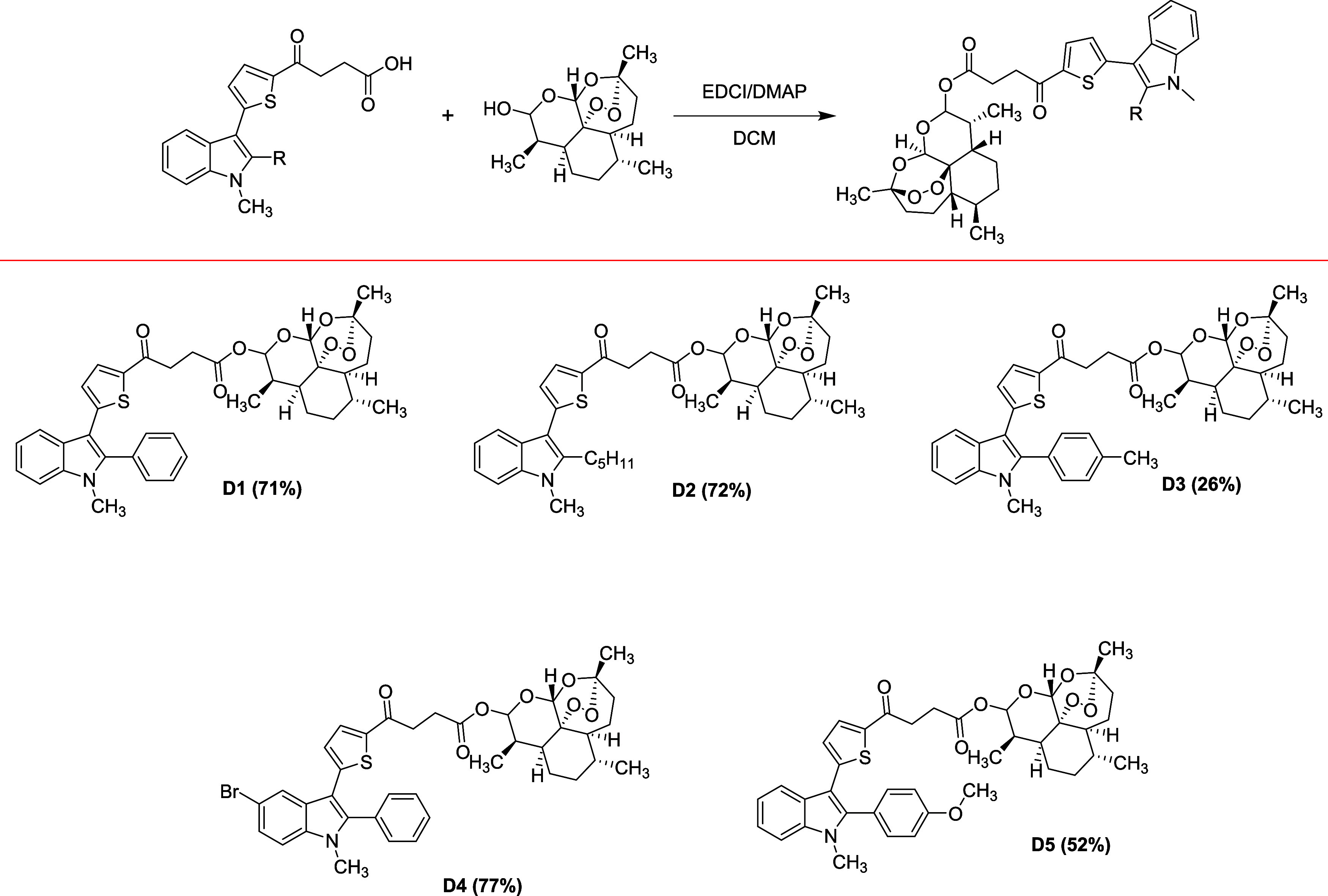
General scheme of synthesized compounds.

According to the literature, the characteristic peaks of Artesunate can be seen around 5.82 parts per million (ppm) as a doublet and 5.44 ppm as a singlet due to the electronegative effect of the oxygen atoms on the ring. There is a singlet at 2.30 ppm that comes from the CH_3_ where the neighbors bridge. As seen in ^1^H NMR spectra, Artesunate’s remaining CH_3_ peaks are detected around 0.96 and 0.84 ppm. The ^13^C NMR spectra of the hybrid compound are important to the determination of carbonyl (C=O) peaks that shift around 190 and 170 ppm in the ^13^C NMR spectrum and the artemisinin structure. There are two carbonyl functional groups on the hybrid molecule, one belonging to the amide and the other belonging to the ester bridge. Moreover, four peaks between 105 and 80 ppm belong to artesunate; the other peaks of artesunate can be seen on the high field of the aliphatic region.^1,33^

All synthesized compounds were characterized using spectroscopic techniques, including NMR (^1^H and ^13^C) and LC-MS/MS spectrometry. The data confirmed the structures and the successful incorporation of functional groups, as intended.

### Antioxidant Capacities of Indole–Artesunate Derivatives

The antioxidant capacity shows different results depending on the oxidant source used. In addition, no analysis method can fully reflect all radical sources or the mechanism of action of all antioxidants in a system.^34,35^ Therefore, different methods for determining the antioxidant capacity are needed. There are many antioxidant capacity methods.^36,37^ These methods are generally divided into two categories: hydrogen atom transfer (HAT) reactions and single electron transfer (SET) reactions. SET-based assays measure the reducing capacity of the antioxidant, whereas HAT-based assays measure the hydrogen atom-donating capacity. In some methods (DPPH and ABTS), both mechanisms can occur together. In this case, the dominant mechanism depends on the structure and properties of the antioxidant compound, together with the solvent system and the dissolution rate in the solvent.^35,38,39^

In this study, four different antioxidant capacity methods were used: DPPH, ABTS, galvinoxyl, and FRAP methods. Galvinoxyl and FRAP methods are SET-based^37,40^ assays, while DPPH and ABTS methods are SET or HAT-based^37,38^ assays.

The amount of antioxidant that scavenges 50% of the radical is expressed as EC_50_. EC_50_ values were determined from standard curve graphs. The EC_50_ values of Trolox used as the standard compound in the DPPH assay were calculated as 24.6 μM. No color loss was seen because none of the compounds under test reacted with the DPPH^•^ radical (Figure 3). As a result, antioxidant capacity could not be measured using this technique. Similarly, antioxidant capacity could not be determined using the ABTS, galvinoxyl, or FRAP techniques because no color change was observed.

**Figure 3:**
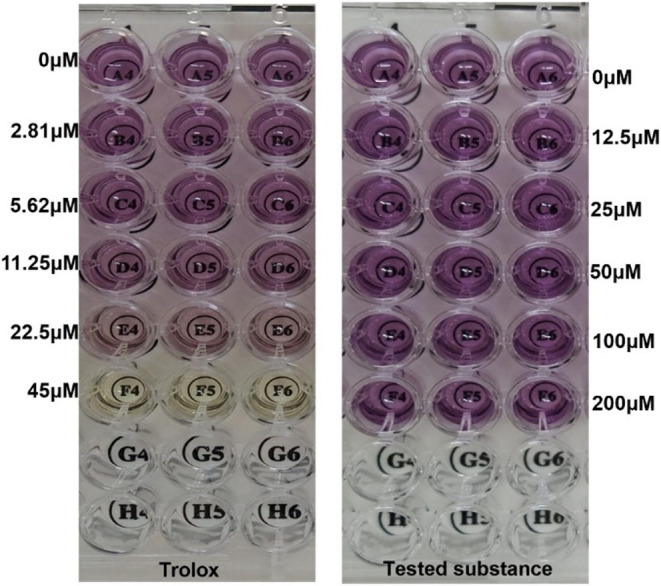
Microplate image of Trolox and test compound in the DPPH assay.

Cancer cells generally produce higher levels of reactive oxygen species (ROS) than do healthy cells. Therefore, they are under oxidative stress and actively utilize their antioxidant systems.^41^ However, high levels of ROS can cause cellular damage in cancer cells.^42^ In fact, cancer cells may be more sensitive to external ROS sources than normal cells.^41,43^ This indicates that a compound under investigation for its anticancer capabilities has antioxidant qualities, which may support the anticancer defenses of cancer cells. As a result, the absence of antioxidant activity in a compound under investigation for anticancer characteristics may be a favorable finding.^41,44^ For these reasons, it is not a bad outcome that the compounds we studied lacked antioxidant properties. This could be seen as a potential benefit for the development of anticancer drugs that specifically work by causing oxidative stress.^44^

### Determination of Anticancer Properties

#### Cytotoxic Analysis

In this study, the tested compounds were prepared at five different concentrations (7.8, 15.6, 31.25, 62.5, and 125 μM) and applied to Ishikawa, HepG2, HT-29, MDA-MB-231, PANC-1, and HUVEC cell lines for 24 h. Their effects on cell viability were investigated by the MTT method. The EC_50_ values obtained after the experimental results are given in a table (Table 1).

**Table 1:** EC_50_ Values of Cell Lines (μM)

compound	Ishikawa	HepG2	HT-29	MDA-MB-231	PANC-1	HUVEC
**D1**	20.22 ± 1.05	5.43 ± 0.91	25.5 ± 0.55	39.19 ± 2.43	64.43 ± 8.21	>125
**D2**	30.57 ± 1.97	5.63 ± 0.02	19.7 ± 0.77	71.21 ± 3.91	34.62 ± 2.84	>125
**D3**	>125	2.75 ± 0.89	32.59 ± 2.45	61.86 ± 2.22	>125	>125
**D4**	>125	>125	>125	>125	>125	>125
**D5**	>125	>125	>125	>125	>125	>125
paclitaxel	19.3 ± 1.21	4.5 ± 1.24	1.4 ± 0.46	14.3 ± 0.23	9.3 ± 1.21	53.7 ± 2.41

The most effective compounds in the Ishikawa cell line were **D1** (EC_50_: 20.22 μM) and **D2** (EC_50_: 30.57 μM). The EC_50_ value of **D1** compound was very close to the EC_50_ value of paclitaxel (19.3 μM) used as a positive control (Table 1).

In the HepG2 cell line, **D1**, **D2**, and **D3** compounds showed good cytotoxic activity. EC_50_ values of **D1** and **D2** compounds (5.43 μM, 5.63 μM) were very close to the EC_50_ value of paclitaxel used as a positive control (4.5 μM), and even the **D3** compound (2.75 μM) showed better activity than the positive control (Table 1). **D1** compound has phenyl in position 2 of the indole ring, **D2** compound has pentyl, while **D3** compound has the *para*-tolyl group (Table 2).

**Table 2:**
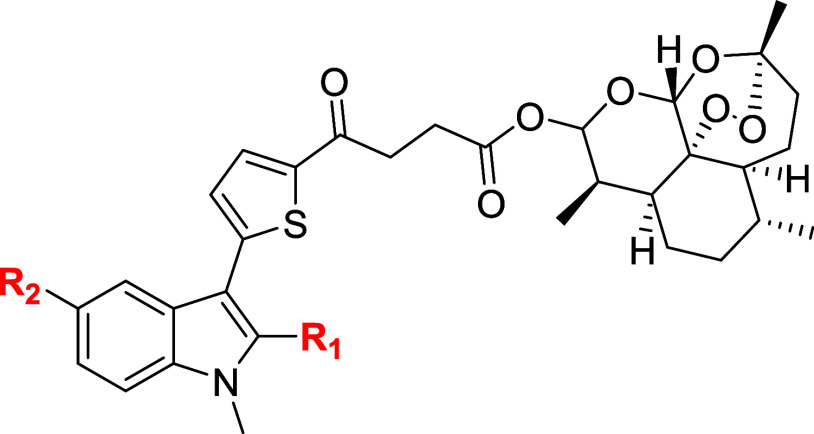
Structures of Tested Compounds

compound	R_1_	R_2_
**D1**	phenyl	H
**D2**	pentyl	H
**D3**	*p*-tolyl	H
**D4**	phenyl	Br
**D5**	*p*-methoxyphenyl	H

The most effective compounds in the HT-29 cell line were compounds **D1**, **D2**, and **D3**. The EC_50_ values of these compounds were 25.5, 19.7, and 32.59 μM, respectively, and the EC_50_ value of the paclitaxel compound used as a positive control was determined as 1.4 μM (Table 1).

The most effective compound in the MDA-MB-231 cell line was compound **D1**. The EC_50_ values of this compound were determined as 39.19 μM, and the EC_50_ value of the paclitaxel compound used as a positive control was determined as 14.3 μM (Table 1).

In the Panc-1 cell line, only compounds **D1** and **D2** had EC_50_ values. EC_50_ values were 64.43 and 34.62 μM, respectively, and the EC_50_ value of the paclitaxel compound used as a positive control was 9.3 μM. For compounds **D4** and **D5**, no EC_50_ value could be calculated for any of the tested cell lines (Table 1).

In the healthy human endothelial (HUVEC) cell line, no EC_50_ value could be detected for any of the tested compounds, while the EC_50_ value of paclitaxel, used as a positive control, was determined as 53.7 μM (Table 1).

Selectivity indices (SI) were calculated using the formula SI = EC_50_ in HUVEC cells/EC_50_ in cancer cells. Because the EC_50_ values of all tested compounds in HUVEC cells were above the highest tested concentration (>125 μM), the SI values should be interpreted as lower-bound estimates rather than exact values. The highest estimated selectivity was observed in HepG2 cells, with SI values of >23 for **D1**, >22.2 for **D2**, and >45.5 for **D3**. However, these findings should be interpreted cautiously because HUVEC was the only noncancerous cell line used in this study. Therefore, the apparent cancer cell-selective cytotoxicity of these compounds should be confirmed in future studies using additional healthy cell models from different tissue origins.

The cytotoxic response of the tested compounds showed cell-line-dependent differences. HepG2 cells appeared to be the most sensitive among the tested cancer cell lines, which may be related to the biological characteristics of hepatocellular carcinoma cells, including high metabolic activity, altered redox homeostasis, mitochondrial vulnerability, and drug-processing capacity. HepG2 cells are widely used in drug metabolism and hepatotoxicity studies, and their liver-derived phenotype may partly influence their response to bioactive hybrid molecules.^45^ In addition, artesunate and its derivatives have been reported to induce cancer cell death through ROS-associated and mitochondrial apoptotic mechanisms.^46,47^ Therefore, the stronger response observed in HepG2 cells may be linked to the interaction between the metabolic and redox characteristics of this cell line and the artesunate-containing hybrid structure of the synthesized compounds. However, this explanation should be interpreted as a possible biological interpretation, rather than a confirmed mechanism.

The electronic effect is more important than reactivity/binding at C-5 of the indole ring. Electron-withdrawing group substituents boost the electron density on the indole ring, particularly at places that are involved in π-stacking or H-bonding. This may make the indole ring more attractive to targets than electron-rich aromatic components. Halogens mostly work via steric/lipophilic actions, inductive withdrawal, and polarizability. Despite its small size, fluorine is routinely used to prevent metabolic oxidation with minimal steric impact. Cl, Br, and I make organic compounds more lipophilic and stable in the biological systems. Br and I are also large and polarizable, enabling halogen bonding in certain protein pockets. According to findings in the literature, **D1** has a biologically active site, whereas **D4** is inactive due to the effect of bromine at the C-5 position of the indole ring.^48^

### Determination of Invasion Capacity

The Matrigel invasion assay was used to measure cancer cells’ ability to invade the basement membrane. In this test, tumor cells were embedded in Matrigel, a matrix that mimics the extracellular matrix. The cells were then exposed to a chemoattractant, such as serum, which encourages them to migrate and degrade the Matrigel. Cells that penetrated the Matrigel and passed to the other side of the porous membrane were stained and observed.^49,50^

The invasive capacities of **D1** and **D2**, which have the most effective cytotoxic properties among the compounds used in this study, were investigated in HepG2, HT-29, and Panc-1 cell lines by using a Matrigel invasion assay.

As a result of the study, it was determined that in the HepG2 cell line, **D1** and **D2** compounds inhibited invasion by 91.2 and 96.6%, respectively, compared to the control group (Figure 4); in the HT-29 cell line, **D1** and **D2** compounds inhibited invasion by 35.1 and 39.2%, respectively, compared to the control group (Figure 5); and in the Panc-1 cell line, **D1** and **D2** compounds inhibited invasion by 97.4 and 94.8%, respectively, compared to the control group (Figure 6).

**Figure 4:**
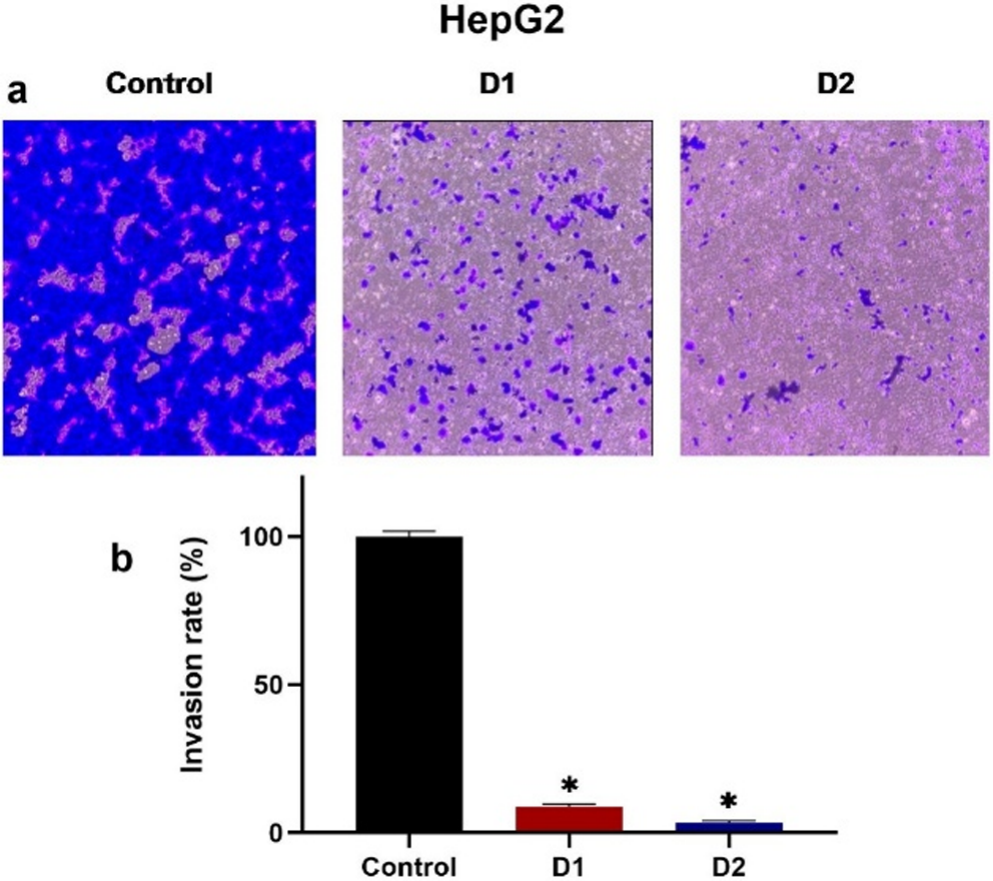
Visualization of the effect of **D1** and **D2** compounds on the invasion capacity of HepG2 cell line (a) and percentage bar chart (b). *Different from the control group (*p* < 0.05).

**Figure 5:**
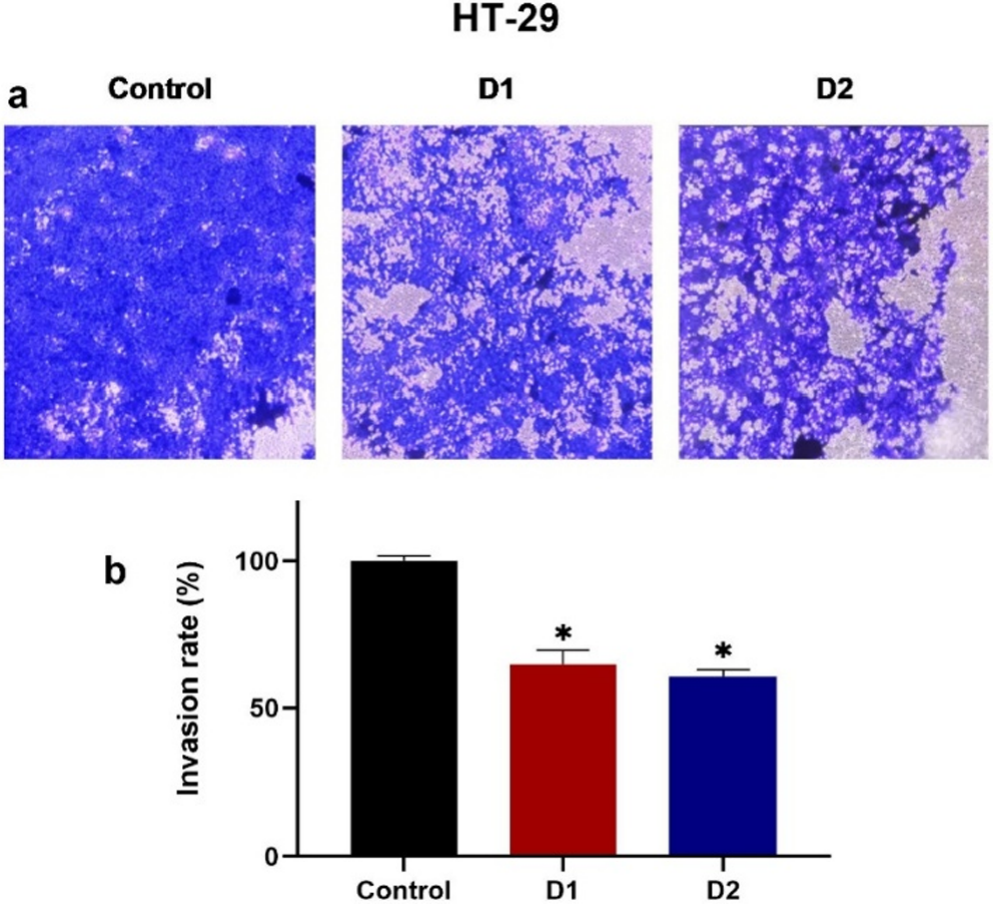
Visualization of the effect of **D1** and **D2** compounds on the invasion capacity of the HT-29 cell line (a) and percentage column chart (b). *Different from the control group (*p* < 0.05).

**Figure 6:**
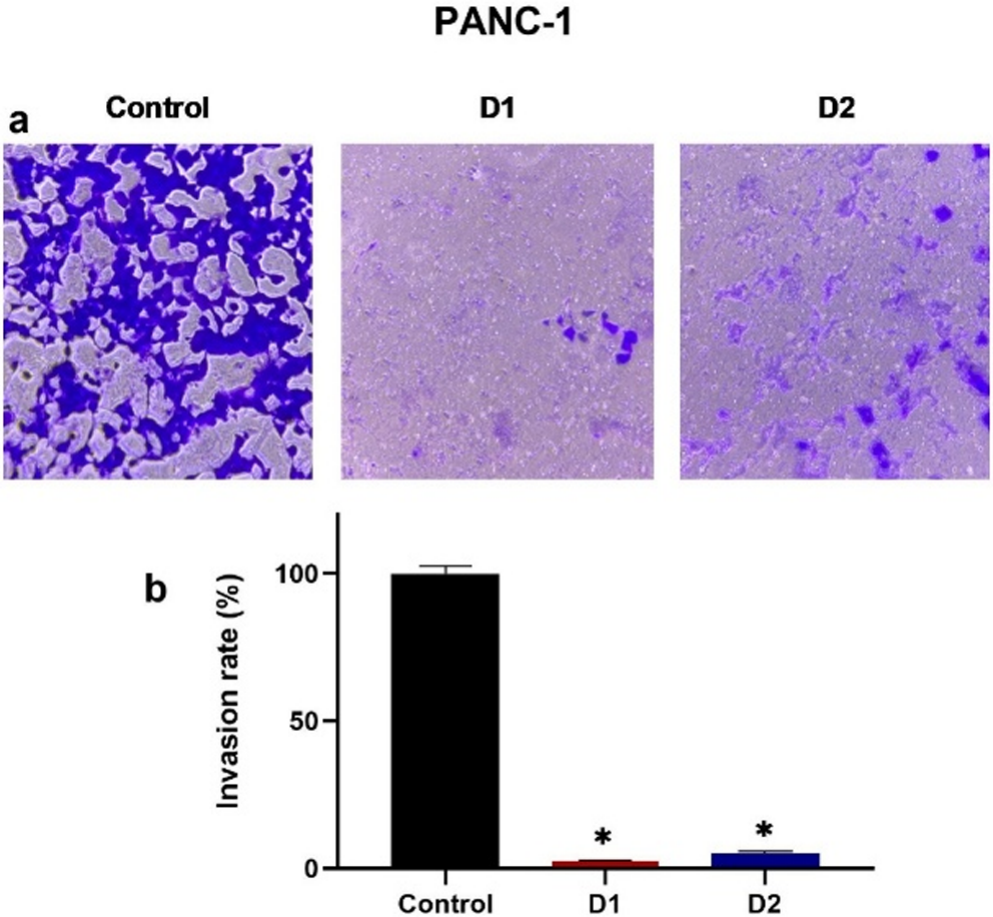
Visualization of the effect of **D1** and **D2** compounds on the invasion capacity of Panc-1 cell line (a) and percentage bar chart (b). *Different from the control group (*p* < 0.05).

Interestingly, PANC-1 cells exhibited a pronounced reduction in invasive capacity despite showing comparatively lower sensitivity in the cytotoxicity assays. This observation suggests that the anti-invasive activity of the synthesized compounds may not be exclusively associated with their cytotoxic effects. Tumor cell invasion is a complex and multifactorial process involving changes in cell adhesion, extracellular matrix degradation, cytoskeletal remodeling, epithelial–mesenchymal transition (EMT), and migration-related signaling pathways. Therefore, compounds capable of interfering with these biological processes may suppress invasion even when their effects on overall cell viability are relatively limited. However, it should be noted that the invasion experiments were performed at concentrations corresponding to EC_50_ values; therefore, the contribution of reduced cell viability to the observed decrease in invasion cannot be completely excluded.^51,52^ Consequently, further studies using subcytotoxic concentrations, together with viability-normalized invasion assays, are required to clarify whether these compounds possess a direct anti-invasive effect independent of their cytotoxic activity.

Cancer cell invasion is a significant contributing factor to cancer-related death. For this reason, researchers frequently target cell invasion in their search for cancer-treating drugs.^53,54^ In one study, they applied artemisinin, a molecule derived from the *A. annua* plant, to human hepatocellular carcinoma (HCC) and HepG2 cell lines at different concentrations. Analyses revealed that artemisinin inhibits invasion by increasing TIMP2, Cdc42, and E-cadherin activity and decreasing metalloproteinase activity.^55^ In another study, they determined the cytotoxic effects of artesunate on glioma cells using the MTT method and then determined the invasive properties of artesunate on glioma cells using Matrigel invasion chambers.^56^ In a study investigating the effect of indole-3-carbinol (I3C) on invasion in the T47-D cell line, they reported that I3C suppressed invasion by increasing PTEN and E-cadherin expression in the T47-D cell line and that similar effects were also observed in the MCF-7 and MDA-MB-468 breast cancer cell lines.^57^

#### Apoptosis Analysis

Normally, the phosphatidylserine membrane lipid found on the inner surface of healthy cells passes to the outer membrane of the cell in the event of apoptosis. In this event, which occurs in the early stage of apoptosis, the cell’s membrane integrity is preserved. In the advanced stage of apoptosis, it begins to deteriorate.^58,59^

Determination of apoptosis by the annexin V/PI method in flow cytometry is a common method that allows distinguishing apoptotic and necrotic cells by measuring the transport of phosphatidylserines in the inner membrane of cells to the outer membrane and propidium iodide (PI) uptake.^60^

In this study, the apoptotic properties of the compounds tested with the flow cytometry device were investigated using the Annexin V-FITC/PI apoptosis kit. Hydrogen peroxide (H_2_O_2_) was used as a positive control because it is a widely used inducer of oxidative-stress-associated apoptosis and provides a reproducible reference for Annexin V/PI-based flow-cytometric apoptosis analysis.^61,62^ However, paclitaxel was not included as an apoptosis-positive control in the present experimental design, although it was used as a cytotoxicity reference compound in the MTT assay. This limitation has been acknowledged, and future studies should include paclitaxel or another clinically relevant anticancer drug as an additional apoptosis-positive control. The results obtained at the end of the test are given in Table 3. Representative Annexin V/PI plots, quadrant definitions, and the flow-cytometric gating strategy are provided in the Supporting Information (Figures S43 and S44).

**Table 3:** Apoptotic Percentages of Compounds Tested for Apoptotic Properties by the Annexin-V/PI Method in Ishikawa, HepG2, MDA-MB-231, HT-29, and PANC-1 Cell Lines Compared to the Control Group

cell lines	control	H_2_O_2_	**D1**	**D2**	**D3**
Ishikawa	3.58 ± 0.62%	14.99 ± 2.05%	16.29 ± 2.20%	24.49 ± 3.15%	
HepG2	1.99 ± 0.35%	12.93 ± 1.40%	20.18 ± 2.45%	12.6 ± 1.31%	15.08 ± 1.85%
HT-29	4.23 ± 0.75%	36.63 ± 3.12%	20.23 ± 3.30%	14.57 ± 1.65%	16.56 ± 2.20%
MDA-MB-231	1.96 ± 0.31%	18.32 ± 2.45%	10.53 ± 1.50%	12.96 ± 1.85%	16.9 ± 3.25%
PANC-1	4.06 ± 0.65%	8.22 ± 1.05%	21.71 ± 2.75%	23.37 ± 2.95%	

In the Ishikawa cell line, the positive control had an apoptotic rate of 14.99%, whereas compounds **D2** and **D1** showed higher activity than the positive control with apoptotic rates of 24.49 and 16.29%, respectively (Table 3).

In the HepG2 cell line, the apoptotic rate in the positive control was 12.93%. Compounds **D1** and **D3** showed higher activity than the positive control, with apoptotic rates of 20.18 and 15.08%, respectively. Compound **D2** (12.6%) also exhibited an apoptotic activity comparable to that of the positive control (Table 3).

In the HT-29 cell line, the apoptotic rate in the positive control was 36.63%. None of the tested compounds (**D1**, **D2**, and **D3**) showed higher activity than the positive control. The highest apoptotic rate was observed with compound **D1** at 20.23% (Table 3).

In the MDA-MB-231 cell line, the positive control had an apoptotic rate of 18.32%, whereas compounds **D1**, **D2**, and **D3** showed lower activity, with apoptotic rates of 10.53, 12.96, and 16.9%, respectively.

In the Panc-1 cell line, the positive control had an apoptotic rate of 8.22%, whereas the **D2** and **D1** compounds showed higher activity than the positive control with apoptotic rates of 23.37 and 21.71%, respectively.

When all of the results were examined, it was found that the compounds’ apoptotic properties varied considerably across cell lines and that they showed the best activity compared with the positive control in the Panc-1 cell line. In contrast, they showed lower activity in the HT-29 cell line (Table 3).

#### Gene Expression Analysis

Two protein families are crucial to apoptosis: One is the Bcl-2 family, which acts as the decision maker of apoptosis, and the other is the caspase family, which serves as the executioner of apoptosis. Bcl-2 family members include antiapoptotic proteins (Bcl-2, Bcl-xL) and proapoptotic proteins (Bax, Bak, Bid, Bim, and Bad), and they tightly control the activation of the mitochondrial apoptotic pathway by regulating mitochondrial homeostasis.^63^ During the apoptosis process, expressions of antiapoptotic genes decrease, while expressions of proapoptotic genes and caspases increase.

The apoptotic properties of compounds, with EC_50_ values determined by the MTT method in PANC-1, MDA-MB-231, HT-29, HepG2, and Ishikawa cell lines, and changes in mRNA expression of Bax, P53, Bcl-2, caspase-3, caspase-8, and caspase-9 genes involved in the apoptotic pathway were investigated by real-time PCR. Changes in mRNA expression (%) of apoptotic pathway genes are shown as column charts for each cell line.

Compared to the control group, the EC_50_ doses of compounds **D1** and **D2** tested in the Ishikawa cell line showed increases in Bax gene expression by 87.3 and 336.1%, respectively; caspase-3 gene expression by 111.4 and 169.1%, respectively; caspase-8 gene expression by 58.1 and 280.6%, respectively; caspase-9 gene expression by 95.4 and 98%, respectively; and P53 gene expression by 201.4 and 324.8%, respectively (Figure 7). While compound **D2** had no statistically significant effect on Bcl-2 gene expression at the EC_50_ dose, compound **D1** reduced this gene expression by 55.7% (Figure 7).

**Figure 7:**
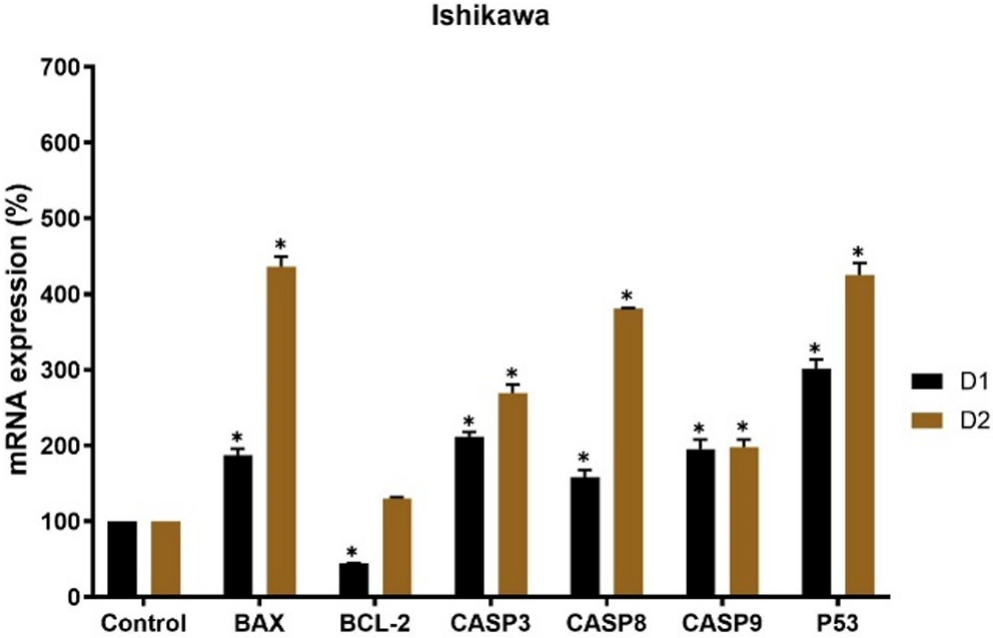
Effects of compounds **D1** and **D2** on mRNA levels of BCL-2, BAX, caspase-3, caspase-8, caspase-9, and P53, which are involved in apoptosis pathways, after the application of EC_50_ doses to the Ishikawa cell line, are shown as a column graph. *Different from the control group (*p* < 0.05).

In the HepG2 cell line, the EC_50_ doses of compounds **D1**, **D2**, and **D3** increased Bax gene expression by 37.7, 149.4, and 27.9%, respectively; caspase-3 gene expression by 227.2, 219.3, and 153.2%, respectively; caspase-8 gene expression by 169.6, 42.5, and 97.3%, respectively; caspase-9 gene expression by 79.7, 85.3, and 462.6%, respectively; and P53 gene expression by 160, 146.4, and 594.1%, respectively, compared to the control group. The EC_50_ doses of compounds **D2** and **D3** did not show a statistically significant effect on Bcl-2 gene expression, while compound **D1** decreased this gene expression by 31.7% (Figure 8).

**Figure 8:**
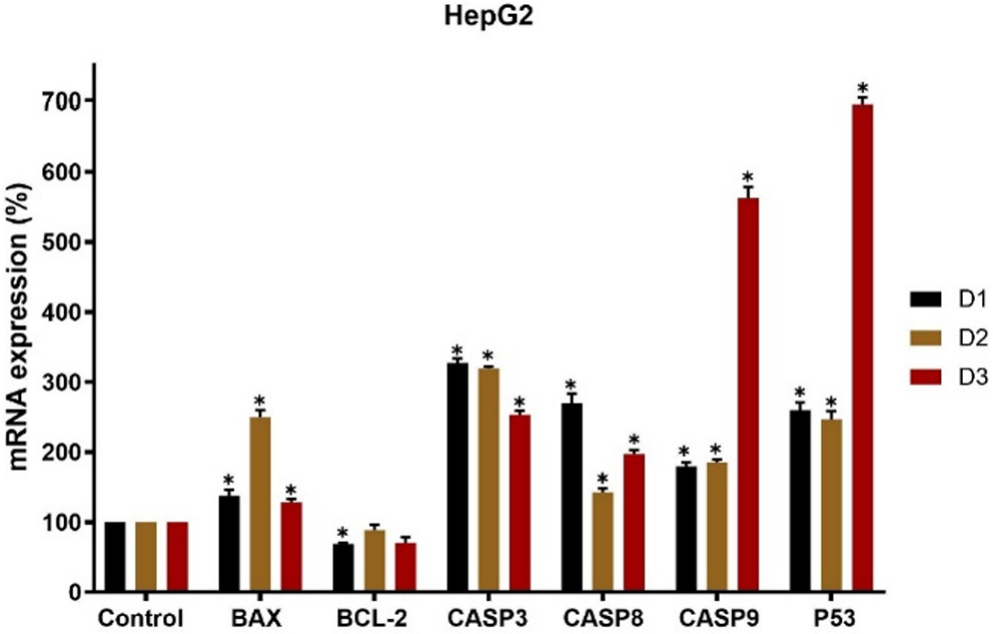
Effects of compounds **D1**, **D2**, and **D3** on the mRNA levels of BCL-2, BAX, caspase-3, caspase-8, caspase-9, and P53, which are involved in apoptosis pathways, after the application of EC_50_ doses to the HepG2 cell line, are shown as a column graph. *Different compared to the control group (*p* < 0.05).

In the HT-29 cell line, the EC_50_ doses of **D1**, **D2**, and their compounds showed no statistically significant effect on Bax gene expression compared with the control group, while compound **D3** increased it by 63.9%. The EC_50_ doses of compounds **D1**, **D2**, and **D3** increased caspase-3 gene expression by 64.1, 88.1, and 100.2%, respectively; caspase-8 gene expression by 136.7, 45.9, and 35.3%, respectively. The EC_50_ doses of compound **D1** did not show a statistically significant effect on caspase-9 gene expression, while compounds **D2** and **D3** increased it by 51.1 and 57.4%, respectively. The EC_50_ doses of compounds **D1**, **D2**, and **D3** increased the expression of the P53 gene by 240.6, 180.5, and 168.7%, respectively. While EC_50_ doses of compounds **D1** and **D2** did not show a statistically significant effect on the expression of the Bcl-2 gene, compound **D3** reduced the level of expression of this gene by 65.1% (Figure 9).

**Figure 9:**
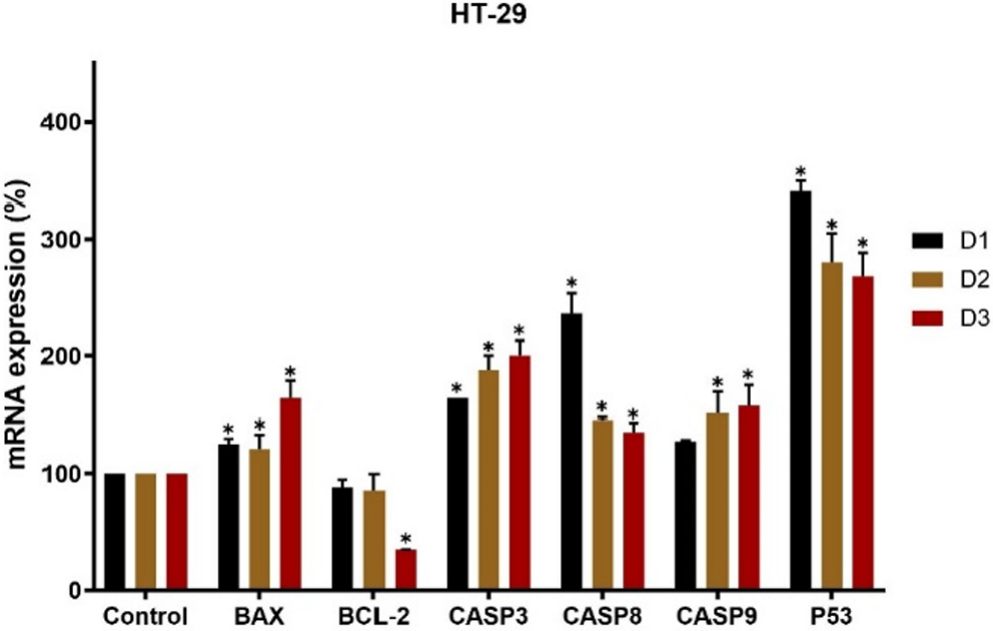
Effects of compounds **D1**, **D2**, and **D3** on mRNA levels of BCL-2, BAX, caspase-3, caspase-8, caspase-9, and P53, which are involved in apoptosis pathways, after the application of EC_50_ doses to the HT-29 cell line, are shown as a column graph. *Different compared to the control group (*p* < 0.05).

Compared to the control group, the EC_50_ doses of compounds **D1**, **D2**, and **D3** tested in the MDA-MB-231 cell line showed increases in Bax gene expression by 146.4, 459.9, and 108.7%, respectively; caspase-3 gene expression by 52.5, 144, and 159.1%, respectively; caspase-8 gene expression by 64.7, 134.1, and 125.2%; caspase-9 gene expression by 102.9, 155.8, and 230.6%; and P53 gene expression by 142.6, 209.8, and 93%, respectively. The EC_50_ doses of compounds **D1**, **D2**, and **D3** did not show a statistically significant effect on the Bcl-2 gene expression (Figure 10).

**Figure 10:**
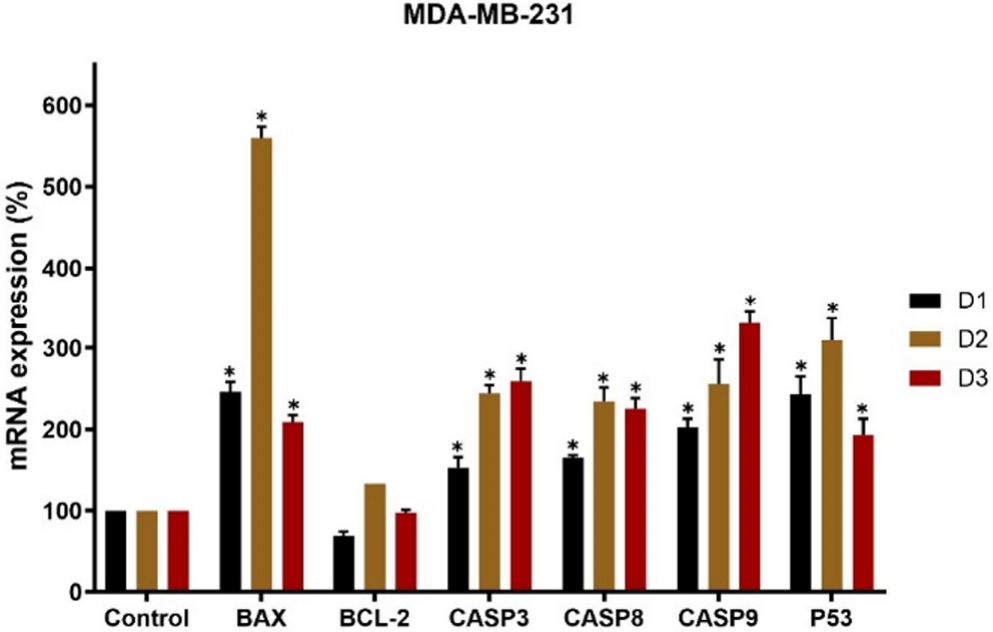
Effects of compounds **D1**, **D2**, and **D3** on the mRNA levels of BCL-2, BAX, caspase-3, caspase-8, caspase-9, and P53, which are involved in apoptosis pathways, after the application of EC_50_ doses to the MDA-MB-231 cell line, are shown as a column graph. *Different compared to the control group (*p* < 0.05).

Compared to the control group, the EC_50_ doses of compounds **D1** and **D2** tested in the PANC-1 cell line showed an increase in Bax gene expression by 63 and 33.8%, respectively; caspase-3 gene expression by 167.9 and 139.6%, respectively; caspase-8 gene expression by 63.5 and 61.9%, respectively; caspase-9 gene expression by 31.6 and 127.9%, respectively; and P53 gene expression by 204.7 and 73.2%, respectively. The EC_50_ doses of compounds **D1** and **D2** decreased the level of Bcl-2 gene expression by 48.8 and 61.6%, respectively (Figure 11).

**Figure 11:**
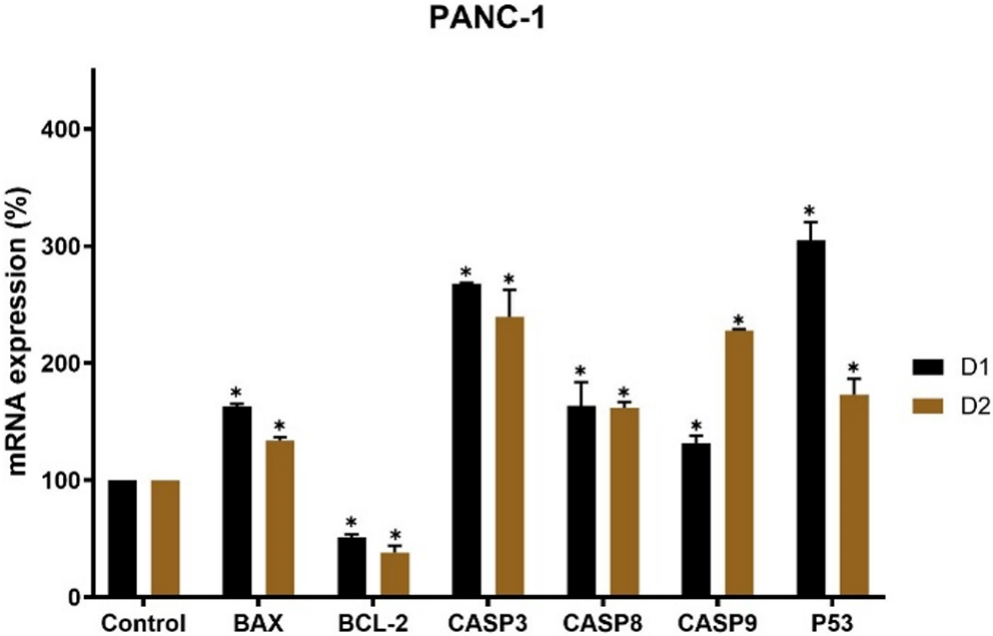
Effects of compounds **D1** and **D2** on the mRNA levels of BCL-2, BAX, caspase-3, caspase-8, caspase-9, and P53, which are involved in apoptosis pathways, after the application of EC_50_ doses to the PANC-1 cell line, are shown as a column graph. *Different compared to the control group (*p* < 0.05).

The tested compounds modulated the expression of apoptosis-related genes by generally decreasing the antiapoptotic marker Bcl-2 while increasing the expression of proapoptotic markers, including Bax, P53, caspase-3, caspase-8, and caspase-9 gene expression, indicating that these compounds may be effective in the apoptotic pathway. When examining the intrinsic and extrinsic pathways of apoptosis, the initiator caspase in the intrinsic pathway is caspase-9, while the initiator caspase in the extrinsic pathway is caspase-8.

Therefore, the increased expression of both caspase-8 and caspase-9 observed in the present study suggests that both intrinsic and extrinsic apoptosis-related signaling pathways may contribute to the observed effects. However, because the present study evaluated mRNA expression rather than caspase protein levels or enzymatic activities, these findings should be interpreted cautiously and cannot alone confirm functional activation of both pathways. Crosstalk between the extrinsic and intrinsic apoptotic pathways may also be involved. Caspase-8 can cleave the cytosolic BH3-only protein Bid to generate truncated Bid (tBid), which subsequently translocates to mitochondria and promotes mitochondrial outer membrane permeabilization (MOMP). This process facilitates the release of cytochrome *c*, apoptosome assembly through interaction with Apaf-1, and subsequent activation of caspase-9, thereby establishing a molecular link between death receptor signaling and mitochondrial apoptosis pathways.^64–66^

In addition, the observed upregulation of TP53 together with increased Bax expression and reduced Bcl-2 expression is consistent with the well-established role of p53 in regulating mitochondrial apoptosis. Activated p53 can transcriptionally induce proapoptotic genes such as BAX, while suppressing antiapoptotic proteins including BCL-2, thereby shifting the cellular balance toward apoptosis.^67,68^ The concomitant increase in caspase-3, the principal executioner caspase, further supports the possibility that apoptotic signaling progresses toward downstream effector mechanisms.^69^ Nevertheless, because gene expression changes do not necessarily correlate with protein abundance or enzymatic activation, additional mechanistic studies including caspase activity assays, Bid/tBid protein analysis, mitochondrial membrane potential measurements, cytochrome *c* release assays, Western blot validation of apoptosis-related proteins, and functional inhibition studies are required to confirm the precise apoptotic mechanism induced by these compounds.

## Conclusion

In this study, we first developed an efficient strategy to conjugate a variety of indole-based artemisinin hybrid compounds, which were synthesized and evaluated for their antioxidant, cytotoxic, anti-invasive, and apoptosis-associated biological effects. The antioxidant potentials of the synthesized compounds were evaluated using DPPH, ABTS, galvinoxyl, and FRAP methods; however, no significant antioxidant activity was detected in any of the tested compounds. Therefore, the biological activity of these compounds should not be interpreted as being associated with antioxidant capacity. The cytotoxic effects of the compounds were examined in different cancer cell lines using the MTT method. In general, compounds **D1**, **D2**, and **D3** were determined to show cytotoxic activity in the tested cell lines. While compound **D1**(EC_50_: 20.22 μM) exhibited activity similar to paclitaxel (EC_50_: 19.3 μM) in the Ishikawa cell line, the activity of compounds **D1** and **D2** (5.43 μM, 5.63 μM) in the HepG2 cell line was found to be comparable to the positive control (4.5 μM). Furthermore, compound **D3** (2.75 μM) was found to exhibit a stronger cytotoxic effect in HepG2 cells than the positive control. In HT-29, MDA-MB-231, and Panc-1 cell lines, compounds **D1** and/or **D2** showed moderate cytotoxic activity. In contrast, no measurable cytotoxicity was observed in any of the compounds tested on the healthy human endothelial cell line (HUVEC). However, this interpretation should be considered cautiously because only one noncancerous cell line was used, and further validation using additional normal cell models is required. Invasion experiments revealed that compounds **D1** and **D2** significantly inhibited cell invasion in HepG2, HT-29, and Panc-1 cell lines compared to control groups. The high invasion suppression observed particularly in HepG2 and Panc-1 cell lines suggests that these compounds may be effective in metastatic processes. The apoptotic effects of the compounds were evaluated using the Annexin V/PI method, and it was found that they significantly affected apoptosis-related processes in all tested cancer cell lines compared to control groups. In the Ishikawa, HepG2, and Panc-1 cell lines, the apoptotic effects of the compounds were observed to be close to or higher than those of hydrogen peroxide, which was used as a positive control. To elucidate the apoptosis mechanism at the molecular level, the mRNA expression levels of BAX, P53, Bcl-2, caspase-3, caspase-8, and caspase-9 genes were analyzed using real-time PCR. The most significant gene expression changes were observed in the Panc-1 cell line. In general, it was determined that the compounds increased the expression of proapoptotic genes and decreased the expression of the antiapoptotic Bcl-2 gene. Overall, the synthesized thiophene-bridged indole–artesunate hybrids, particularly **D1**, **D2**, and **D3**, represent promising anticancer candidates with cytotoxic, anti-invasive, and apoptosis-associated effects. Further mechanistic and preclinical studies are required to clarify their precise molecular mechanisms, confirm their selectivity, and evaluate their potential for anticancer drug development.

## Materials and Methods

### Chemicals and Materials

All synthesized molecules were determined by ^1^H NMR, ^13^C NMR, and LC-MS/MS. ^1^H and ^13^C NMR spectra were obtained by using an Agilent NMR (400 MHz) spectrometer and a JEOL ECZ500R. Chemical shifts were reported in parts per million (ppm) downfield from an internal tetramethyl silane (TMS) reference. Thick-walled glass columns and “flash grade” silica (Merck 230–400 mesh) were used for flash chromatography. A commercially prepared 0.25 mm silica gel plate was applied for thin-layer chromatography (TLC). All starting chemicals and catalysts were purchased from commercial sources with high purity. The chemicals and solvents were used directly from commercial stock, as mentioned in the method. High-resolution mass spectra (LC-MS/MS) were recorded on a Thermo Scientific Q Exactive mass spectrometer equipped with an APCI source. Anton Paar GmbH - Monowave 300 (Microwave synthesis reactor) was used for the synthesis of intermediates.

#### General Procedure for Synthesis of 2-Aryl/alkyl-1-methyl-3-(thiophen-2-yl)-1*H*-indole

*N*,*N*-*d*imethyl-2-iodo aniline (1 mmol) dissolved in Et_3_N (6 mL) under argon, Pd­(PPh_3_)_2_Cl_2_ (0.05 mmol), aryl/alkyl terminal alkyne (1.2 mmol), CuI (0.05 mmol), respectively, were added. The mixture was afforded in a microwave device at 60 °C/1 h/800 rpm. Product conversion was checked with TLC, and 2-iodothiophene (3 mmol) was added to the mixture with acetonitrile (CH_3_CN) (3 mL) under argon inert gas. The mixture was allowed to react a second time in a microwave device at 90 °C/1 h/800 rpm. The resulting mixture was poured onto 20 mL of distilled water, the water phase was washed with chloroform (3 × 40 mL), the organic phase was dried over MgSO_4_, filtered, and concentrated under vacuum. It was purified by column chromatography using a hexane solvent system.

#### Synthesis of 1-Methyl-2-phenyl-3-(thiophen-2-yl)-1*H*-indole (**3a**)

*N*,*N*-dimethyl-2-iodo aniline (8.1 mmol, 2000 mg), Et_3_N (6 mL), Pd­(PPh_3_)_2_Cl_2_ (284 mg, 0.4 mmol), phenylacetylene (**1a**) (911 mg, 8.9 mmol), CuI (77 mg, 0.41 mmol), acetonitrile (CH_3_CN) (3 mL), and 2-iodothiophene (5.671 g, 27.1 mmol) were used in the reaction. The targeted product was obtained with 1887 mg, 72% yield. ^1^H NMR (400 MHz, CDCl_3_) δ 8.03–7.97 (m,1H), 7.52–7.40 (m, 6H), 7.38–7.32 (m, 1H), 7.31–7.24 (m, 1H), 7.19–7.14 (m, 1H), 7.02–6.93 (m, 1H), 3.64 (s, 3H) (Figure S1). ^13^C NMR (100 MHz, CDCl_3_) δ 138.5, 137.4, 137.2, 131.9, 131.4, 128.8, 128.7, 127.0, 126.9, 124.9, 123.5, 122.6, 120.7, 120.1, 109.8, 108.7, 31.1 (Figure S2).^70^

#### Synthesis of 1-Methyl-2-pentyl-3-(thiophen-2-yl)-1*H*-indole (**3b**)

*N*,*N*-*d*imethyl-2-iodo aniline (0.80 mmol, 200 mg), Et_3_N (6 mL), Pd­(PPh_3_)_2_Cl_2_ (0.04 mmol, 28 mg), hept-1-ine (1b) (0.97 mmol, 93 mg), CuI (0.04 mmol, 8 mg), acetonitrile (CH_3_CN) (3 mL), and 2-iodothiophene (2.42 mmol, 510 mg) were used in the reaction. The targeted product obtained 160 mg, 70% yield. ^1^H NMR (400 MHz, CDCl_3_) δ 7.80 (dq, *J* = 8.0; 1.1 Hz, 1H), 7.35–7.31 (m, 2H), 7.27–7.22 (m, 1H), 7.19–7.14 (m, 2H), 7.12 (dd, *J* = 3.5; 1.2 Hz, 1H), 3.75 (s, 3H), 2.94 (t, *J* = 8.0 Hz, 2H), 1.73–1.63 (m, 2H), 1.47–1.31 (m, 4H), 0.92 (t, *J* = 7.0 Hz, 3H) (Figure S3). ^13^C NMR (100 MHz, CDCl_3_) δ 139.3, 137.7, 136.9, 127.5, 127.4, 125.0, 123.8, 121.7, 120.2, 119.3, 109.0, 107.0, 32.0, 30.2, 30.0, 25.3, 22.6, 14.3 (Figure S4).

#### Synthesis of 1-Methyl-2-*p*-tolyl-3-(thiophen-2-yl)-1*H*-indole (**3c**)

*N*,*N*-dimethyl-2-iodo aniline (0.80 mmol, 200 mg), Et_3_N (6 mL), Pd­(PPh_3_)_2_Cl_2_ (0.04 mmol, 28 mg), *p*-tolyl acetylene (**1c**) (0.97 mmol, 113 mg), CuI (0.04 mmol, 8 mg), acetonitrile (CH_3_CN) (3 mL), and 2-iodothiophene (2.42 mmol, 510 mg) were used in the reaction. The target product was obtained with 244 mg, 99% yield. ^1^H NMR (400 MHz, CDCl_3_) δ 7.97 (dq, *J* = 8.0; 1.1 Hz, 1H), 7.41 (dt, *J* = 8.2; 1.0 Hz, 1H), 7.36–7.22 (m, 6H), 7.15 (dd, *J* = 5.1; 1.2 Hz, 1H), 7.01–6.97 (m, 1H), 6.96 (dd, *J* = 3.6; 1.2 Hz, 1H), 3.63 (s, 3H), 2.45 (s, 3H) (Figure S5). ^13^C NMR (100 MHz, CDCl_3_) δ 138.8, 138.7, 137.6, 137.2, 131.3, 129.5, 128.8, 127.0, 126.9, 124.9, 123.4, 122.5, 120.6, 120.0, 109.7, 108.5, 31.1, 21.7 (Figure S6). LC/MS-MS [M + Na]^+^ calc. for C_20_H_17_NS, 326.0979; found, 326.0976.

#### Synthesis of 2-(4-Methoxyphenyl)-1-methyl-3-(thiophen-2-yl)-1*H*-indole (**3d**)

*N*,*N*-dimethyl-2-iodo aniline (0.80 mmol, 200 mg), Et_3_N (6 mL), Pd­(PPh_3_)_2_Cl_2_ (0.04 mmol, 28 mg), 1-ethynyl-4-methoxybenzene (**1d**) (0.97 mmol, 128 mg), CuI (0.04 mmol, 8 mg), acetonitrile (CH_3_CN) (3 mL), and 2-iodothiophene (2.42 mmol, 510 mg) were used in the reaction. The targeted product was obtained with 190 mg, 74% yield. ^1^H NMR (400 MHz, CDCl_3_) δ 7.98 (dq, *J* = 8.0; 0.7 Hz, 1H), 7.41 (d, *J* = 8.1 Hz, 1H), 7.37–7.30 (m, 3H), 7.26 (td, *J* = 8.1; 1.2 Hz, 1H), 7.16 (dd, *J* = 5.0; 1.3 Hz, 1H), 7.04–6.96 (m, 4H), 3.89 (s, 3H), 3.62 (s, 3H) (Figure S8). ^13^C NMR (100 MHz, CDCl_3_) δ 160.1, 138.4, 137.7, 137.1, 132.6, 127.0, 126.8, 124.7, 123.9, 123.3, 122.4, 120.6, 120.0, 114.2, 109.7, 108.5, 55.5, 31.0 (Figure S8). LC/MS-MS [M + H]^+^ calc. C_20_H_17_NOS 320.1104: found 320.1112.

#### Synthesis of 4-Bromo-*N*,*N*-dimethyl-2-(phenylethynyl)­aniline (**a**)

4-Bromo-2-iodo-*N*,*N*-dimethylaniline (1000 mg, 1.53 mmol) is dissolved in 10 mL of Et_3_N under argon gas at room temperature. Pd­(PPh_3_)_2_Cl_2_ (108 mg, 0.14 mmol), phenylacetylene (344 mg, 3.36 mmol), and CuI (27 mg, 0.14 mmol) were added sequentially, and the mixture was stirred at room temperature overnight. The mixture is controlled by TLC. Chloroform (60 mL) was added and washed with 3 × 20 mL distilled water, and the organic phase was dried with MgSO_4_. Column chromatography with ethyl acetate/*n*-Hexane (1:100) yielded 900 mg, 98% yield of the targeted product. ^1^H NMR (400 MHz, CDCl_3_) δ 7.57–7.50 (m, 3H), 7.38–7.31 (m, 5H), 3.0 (s, 6H) (Figure S9).^71^

#### Synthesis of 5-Bromo-3-iodo-1-methyl-2-phenyl-1*H*-indole (**b**)

4-Bromo-*N*,*N*-dimethyl-2-(phenylethynyl)­aniline (**a**) (890 mg, 2.96 mmol) was stirred in 20 mL of DCM under argon. Molecular iodine (1500 mg, 5.92 mmol) was added, and the mixture was stirred at room temperature overnight. The reaction is finished by adding 30 mL of saturated Na_2_S_2_O_3_. The organic phase is washed with 1 × 20 mL of distilled water, and the organic phase is dried over MgSO_4_. The crude product was purified using column chromatography with ethyl acetate/*n*-hexane (1:100). The target product was obtained with 780 mg, 64% yield. ^1^H NMR (500 MHz, CDCl_3_) δ 7.66 (d, *J* = 1.9 Hz, 1H), 7.56–7.49 (m, 3H), 7.47–7.45 (m, 2H), 7.38 (dd, *J* = 8.6:1.9 Hz, 1H), 7.20 (d, *J* = 10.0 Hz, 1H), 3.67 (s, 3H) (Figure S10). ^13^C NMR (125 MHz, CDCl_3_) δ 143.1, 136.7, 132.3, 131.3, 131.0, 129.1, 128.7, 125.9, 124.2, 114.2, 111.6, 58.0, 32.4 (Figure S11).^72^

#### Synthesis of 5-Bromo-1-methyl-2-phenyl-3-(thiophen-2-yl)-1*H*-indole (**3e**)

5-Bromo-3-iodo-1-methyl-2-phenyl-1*H*-indole (**b**) (100 mg, 0.24 mmol) was dissolved in DMF (2.5 mL) under argon. EtOH (2.5 mL), Pd­(PPh_3_)_4_ (56 mg, 0.05 mmol), 2-thiophenboronic acid (31 mg, 0.24 mmol), K_2_CO_3_ (50 mg, 0.36 mmol), and deionized water (1 mL) were added, respectively, at an inert atmosphere and room temperature. The mixture was left in the microwave reactor at 75 °C for 1 h, then controlled with TLC, and 30 mL of ethyl acetate was added, and the organic phase was washed with 3 × 10 mL of brine, dried over MgSO_4_, and filtered, and the solvent was removed by evaporator under reduced pressure. Purified by column chromatography using ethyl acetate/*n*-hexane (1:5 mL) were employed to afford 79 mg of the desired product, 89% yield. ^1^H NMR (500 MHz, CDCl_3_) δ 7.95 (d, *J* = 1.8 Hz, 1H), 7.39–7.35 (m, 3H), 7.31–7.27 (m, 3H), 7.18–7.14 (m, 1H), 6.81 (dd, *J* = 3.5:1.0 Hz, 1H), 3.5 (s, 3H) (Figure S12). ^13^C NMR (125 MHz, CDCl_3_) δ 130.5, 136.5, 135.9, 131.3, 131.2, 129.1, 128.8, 128.6, 127.2, 125.4, 125.3, 124.0, 122.6, 114.0, 11.3, 108.3, 31.3 (Figure S13).

#### General Procedure for Synthesis of 4-(5-(2-Aryl/alkyl-1-methyl-1*H*-indol-3-yl)­thiophen-2-yl)-4-oxobutanoic Acid

2-Aryl/alkyl-1-methyl-3-(thiophen-2-yl)-1*H*-indole (1 mmol) and succinic anhydride (2 mmol) dissolved in DCM (10 mL) 30 min after AlCl_3_ (3 mmol) were added piece by piece in an ice bath under argon, and the mixture was brought to room temperature until the reaction was completed. The pH is adjusted to 5–6 with 2 M HCl solution. The water phase was washed with chloroform (3 × 50 mL). The organic phase was dried over MgSO_4_, filtered, and the organic solvent was concentrated under vacuum. It was purified by column chromatography using the hexane/ethyl acetate (1/1) solvent system to give the desired product.

#### Synthesis of 4-(5-(1-Methyl-2-phenyl-1*H*-indol-3-yl)­thiophen-2-yl)-4-oxobutanoic Acid (**4a**)

1-Methyl-2-phenyl-3-(thiophen-2-yl)-1*H*-indole (**3a**) (1 mmol, 289 mg), succinic anhydride (2 mmol, 200 mg), DCM (10 mL), and AlCl_3_ (3 mmol, 400 mg) were employed to afford 150 mg of the desired product, 39% yield. ^1^H NMR (400 MHz, CDCl_3_) δ 8.03 (dt, *J* = 7.8; 0.6 Hz, 1H), 7.57 (d, *J* = 4 Hz, 1H), 7.52–7.45 (m, 2H), 7.41–7.26 (m, 6H), 6.81 (d, *J* = 4.0 Hz, 1H), 3.60 (s, 3H), 3.17 (t, *J* = 8.2 Hz, 2H), 2.76 (t, *J* = 6.7 Hz, 2H) (Figure S14). ^13^C NMR (100 MHz, CDCl_3_) δ 190.5, 178.2, 147.7, 140.0, 139.8, 137.4, 132.9, 131.3, 131.2, 129.5, 129.2, 126.2, 125.3, 123.1, 121.4, 120.1, 110.1, 108.2, 33.5, 31.1, 28.5 (Figure S15). LC/MS-MS [M + Na]^+^ calc. C_23_H_19_NO_3_SNa 412.0982: found 412.0988 (Figure S34).

#### Synthesis of 4-(5-(1-Methyl-2-pentyl-1*H*-indol-3-yl)­thiophen-2-yl)-4-oxobutanoic Acid (**4b**)

1-Methyl-2-pentyl-3-(thiophen-2-yl)-1*H*-indole (**3b**) (0.53 mmol, 150 mg), succinic anhydride (0.79 mmol, 79 mg) dissolved in DCM (10 mL), and AlCl_3_ (4.23 mmol, 565 mg) were employed to afford 120 mg of the desired product, 59% yield. ^1^H NMR (400 MHz, CDCl_3_) δ 8.05–8.03 (m, 1H), 7.78–7.75 (m, 2H), 7.37–7.32 (m, 1H), 7.19–7.14 (m, 1H), 7.09 (dd, *J* = 3.5; 1.2 Hz, 1H), 3.8 (s, 3H), 3.46–3.37 (m, 2H), 2.97–2.89 (m, 2H), 2.87–278 (m, 2H), 1.73–1.60 (m, 3H), 1.45–1.30 (m, 3H), 0.89 (t, *J* = 7 Hz, 3H) (Figure S16). ^13^C NMR (100 MHz, CDCl_3_) δ 198.2, 178.4, 143.8, 141.0, 136.5, 130.1, 127.6, 125.6, 124.5, 121.1, 120.4, 118.9, 109.9, 107.8, 33.6, 31.9, 30.3, 29.9, 28.7, 25.5, 22.6, 14.2 (Figure S17). LC/MS-MS [M + H]^+^ calc. for C_22_H_26_NO_3_S 384.1627: Found 384.1639 (Figure S35).

#### Synthesis of 4-(5-(1-Methyl-2-(*p*-tolyl)-1*H*-indol-3-yl)­thiophen-2-yl)-4-oxobutanoic Acid (**4c**)

1-Methyl-3-(thiophen-2-yl)-2-(*p*-tolyl)-1*H*-indole (**3c**) (0.62 mmol, 190 mg), succinic anhydride (0.62 mmol, 63 mg), DCM (10 mL), and AlCl_3_ (5 mmol, 668 mg) were employed to afford 140 mg of the desired product, 55% yield. ^1^H NMR (400 MHz, CDCl_3_) δ ^1^H NMR (400 MHz, CDCl_3_) δ 8.05–7.92 (m, 2H), 7.85 (d, *J* = 8.5 Hz, 1H), 7.58 (d, *J* = 2.9 Hz, 1H), 7.40 (d, *J* = 8.2 Hz, 1H), 7.23–7.14 (m, 3H), 7.01–6.90 (m, 2H), 3.67 (s, 3H), 3.45 (d, *J* = 6.6 Hz, 2H), 2.85 (d, *J* = 8.8 Hz, 2H), 2.43 (s, 3H) (Figure S18). ^13^C NMR (100 MHz, CDCl_3_) δ 197.9, 178.2, 130.9, 130.7, 129.6, 129.3, 127.8, 127.0, 126.9, 125.3, 125.1, 123.9, 123.7, 122.3, 121.6, 120.4, 119.4, 110.3, 33.4, 31.0, 28.4, 21.4 (Figure S19). LC/MS-MS [M + H]^+^ calc. for C_24_H_21_NO_3_SNa 426.1139: found 426.1143 (Figure S36).

#### Synthesis of 4-(5-(5-Bromo-1-methyl-2-phenyl-1*H*-indol-3-yl)­thiophen-2-yl)-4-oxobutanoic Acid (**4d**)

5-Bromo-1-methyl-2-phenyl-3-(thiophen-2-yl)-1*H*-indole (**3d**) (1.25 mmol, 460 mg), succinic anhydride (1.75 mmol, 137 mg) dissolved in DCM (10 mL), and AlCl_3_ (9.9 mmol, 1332 mg) were employed to afford 480 mg of the desired product, 82% yield. ^1^H NMR (500 MHz, CDCl_3_) δ 8.12 (d, *J* = 1.8 Hz, 1H), 7.60 (d, *J* = 4 Hz, 1H), 7.53–7.49 (m, 3H), 7.43 (dd, *J* = 8.8:1.7 Hz, 1H), 7.40–7.37 (m, 2H), 7.29 (d, *J* = 8.7 Hz, 1H), 6.82 (d, *J* = 4.1 Hz, 1H), 3.60 (s, 3H), 3.20 (t, *J* = 6.7 Hz, 2H), 2.78 (t, *J* = 6.7 Hz, 2H) (Figure S20). ^13^C NMR (125 MHz, CDCl_3_) δ 197.8, 177.5, 140.3, 136.0, 133.0 (2C), 131.1, 129.8, 129.2, 127.8, 126.7, 125.9, 125.7, 122.5, 119.4, 114.8, 111.6, 107.8, 33.5, 31.3, 28.2 (Figure S21). LC/MS-MS [M + H]^+^ calc. for C_23_H_18_BrNO_3_S 468.0263: found 468.0282.

#### Synthesis of 4-(5-(2-(4-Methoxyphenyl)-1-methyl-1*H*-indol-3-yl)­thiophen-2-yl)-4-oxobutanoic Acid (**4e**)

2-(4-Methoxyphenyl)-1-methyl-3-(thiophen-2-yl)-1*H*-indole (**3e**) (0.59 mmol, 190 mg), succinic anhydride (0.59 mmol, 60 mg), DCM (10 mL), and dissolved in AlCl_3_ (4.75 mmol, 635 mg) were employed to afford 150 mg of the desired product, 60% yield. ^1^H NMR (400 MHz, CDCl_3_) δ 8.11–8.09 (m, 1H), 8.00–8.90 (m, 1H), 7.86–7.81 (m, 1H), 7.40–7.35 (m, 1H), 7.33–7.25 (m, 2H),7.19–7.13 (m, 1H), 7.04–6.94 (m, 3H), 3.86 (s, 3H), 3.63 (dd, *J* = 20.8; 2.7 Hz, 3H), 3.48–3.40 (m, 2H), 2.88–2.80 (m, 2H) (Figure S22). ^13^C NMR (100 MHz, CDCl_3_) δ 197.9, 178.6, 160.1, 142.1, 139.4, 139.4, 136.4, 132.1, 130.4, 126.9, 124.9, 123.6, 122.8, 122.2, 121.5, 120.3, 119.3, 114.0, 110.3, 55.2, 33.3, 30.9, 28.4 (Figure S23). LC/MS-MS [M + H]^+^ Calc. for C_24_H_21_NO_4_SH 420.1264: found 420.1275 (Figure S37).

#### General Procedure for Synthesis of (3*R*,5*aS*,6*R*,8*aS*,9*R*,12*R*,12*aR*)-3,6,9-Trimethyldecahydro-12*H*-3,12-epoxy­[1,2]­dioxepino­[4,3-i]­isochromen-10-yl 4-(5-(2-(aryl/alkyl)-1-methyl-1*H*-indol-3-yl)­thiophen-2-yl)-4-oxobutanoate

4-(5-(2-(Aryl/alkyl)-1-methyl-1*H*-indol-3-yl)­thiophen-2-yl)-4-oxobutanoic acid (1 mmol), dihydroartemisinin (1.5 mmol) dissolved in DCM (10 mL) an ice bath under argon, EDCI (4 mmol), and dimethylaminopyridine (DMAP) (1 mmol) were added, respectively, 1 h after addition, and the mixture was brought to room temperature and stirred at room temperature overnight. Conversion of the starting material was followed by thin-layer chromatography (TLC). The reaction was terminated after the starting material was completely consumed. Then, 30 mL of DI water was added and washed with chloroform (3 × 50 mL). The organic phase was dried with MgSO4 and filtered, and the organic solvent was removed under vacuum. It was purified using the hexane/ethyl acetate (5/1) solvent system to give the desired product (Figure 2).

#### Synthesis of (3*R*,5*aS*,6*R*,8*aS*,9*R*,12*R*,12*aR*)-3,6,9-Trimethyldecahydro-12*H*-3,12-epoxy­[1,2]­dioxepino­[4,3-*i*]­isochromen-10-yl 4-(5-(1-methyl-2-phenyl-1*H*-indol-3-yl)­thiophen-2-yl)-4-oxobutanoate (**D1**)

4-(5-(1-Methyl-2-phenyl-1*H*-indol-3-yl)­thiophen-2-yl)-4-oxobutanoic acid (**4a**) (0.09 mmol, 36 mg), DCM (5 mL), dihydroartemisinin (0.13 mmol, 40 mg), EDCI (0.36 mmol, 71 mg), and DMAP (0.09 mmol, 11 mg) were employed to afford 43 mg of the desired product with 71% yield. ^1^H NMR (400 MHz, CDCl_3_) δ 8.02 (d, *J* = 7.8 Hz, 1H), 7.57 (d, *J* = 4.0 Hz, 1H), 7.51–7.44 (m, 3H), 7.42–7.37 (m, 3H), 7.34 (td, *J* = 7.0; 1.1 Hz, 1H), 7.30–7.24 (m, 1H), 6.78 (d, *J* = 4.0 Hz, 1H), 5.78 (d, *J* = 9.9 Hz, 1H), 5.41 (s, 1H), 3.59 (s, 3H), 3.30–3.07 (m, 2H), 2.91–2.71 (m, 2H), 6.64–6.50 (m, 1H), 2.41–2.30 (m, 1H), 2.07–1.97 (m, 2H), 1.92–1.84 (m, 2H), 1.82–1.66 (m, 4H), 1.63–1.53 (m, 2H), 1.54–1.44 (m, 2H), 1.41 (s, 3H), 1.34–1.19 (m, 4H), 0.94 (d, *J* = 6.1 Hz, 3H), 0.84 (d, *J* = 7.1 Hz, 3H) (Figure S24). ^13^C NMR (100 MHz, CDCl_3_) δ 190.5, 171.9, 147.4, 140.0, 139.8, 137.3, 132.8, 131.3, 131.1, 129.5, 129.1, 126.1, 125.2, 123.0, 121.4, 120.1, 110.0, 108.2, 104.6, 92.3, 91.7, 80.3, 51.7, 45.4, 37.4, 36.4, 35.0, 34.3, 32.0, 31.1, 28.7, 26.2, 24.8, 22.2, 20.4, 12.3 (Figure S25). LC/MS-MS [M + Na]^+^ calc. for C_38_H_41_NO_7_SNa 678.2501: found 678.2510 (Figure S38).

#### Synthesis of (3*R*,5*aS*,6*R*,8*aS*,9*R*,12*R*,12*aR*)-3,6,9-Trimethyldecahydro-12*H*-3,12-epoxy­[1,2]­dioxepino­[4,3-*i*]­isochromen-10-yl 4-(5-(1-methyl-2-pentyl-1*H*-indol-3-yl)­thiophen-2-yl)-4-oxobutanoate (**D2**)

4-(5-(1-Methyl-2-pentyl-1*H*-indol-3-yl) thiophen-2-yl)-4-oxobutanoic acid (**4b**) (0.35 mmol, 100 mg), DCM (5 mL), dihydroartemisinin (0.53 mmol, 203 mg), EDCI (1.41 mmol, 291 mg), and DMAP (0.35 mmol, 43 mg) were employed to afford 122 mg of the desired product with 72% yield. ^1^H NMR (400 MHz, CDCl_3_) δ 8.03–8.01 (m, 1H), 7.86–7.80 (m, 1H), 7.79–7.74 (m, 1H), 7.36–7.31 (m, 1H), 7.17–7.14 (m, 1H), 7.09 (dd, *J* = 3.5:1.2 Hz, 1H), 5.82 (d, *J* = 9.8 Hz, 1H), 5.44 (s, 1H), 3.81 (s, 3H), 3.55–3.40 (m, 2H), 3.00–2.85 (m, 2H), 2.65–2.50 (m, 1H), 2.42–2.30 (m, 2H), 2.07–1.97 (m, 3H), 1.92–1.82 (m, 4H), 1.78–1.60 (m, 5H), 1.54–1.22 (m, 8H), 0.98–0.86 (m, 11H) (Figure S26). ^13^C NMR (100 MHz, CDCl_3_) δ 197.7171.9, 143.4, 136.2, 131.2, 130.0, 127.3, 125.3, 124.2, 121.9, 120.6, 120.2, 119.1, 118.6, 109.6, 104.0, 96.3, 87.7, 81.1, 52.5, 51.5, 45.4, 44.3, 37.4, 36.3, 34.7, 34.1, 31.7, 30.7, 26.0, 25.6, 24.7, 22.1, 20.4, 13.9, 13.2, 12.7 (Figure S27). LC/MS-MS [M + Na]^+^ calc. for C_37_H_47_NO_7_SNa 672.2965: found 672.2985 (Figure S39).

#### Synthesis of (3*R*,5*aS*,6*R*,8*aS*,9*R*,12*R*,12*aR*)-3,6,9-Trimethyldecahydro-12*H*-3,12-epoxy­[1,2]­dioxepino­[4,3-*i*]­isochromen-10-yl 4-(5-(1-methyl-2-(*p*-tolyl)-1*H*-indol-3-yl)­thiophen-2-yl)-4-oxobutanoate (**D3**)

4-(5-(1-Methyl-2-(*p*-tolyl)-1*H*-indol-3-yl) thiophen-2-yl)-4-oxobutanoic acid (**4c**) (0.25 mmol, 100 mg), DCM (5 mL), dihydroartemisinin (0.38 mmol, 109 mg), EDCI (1.02 mmol, 196 mg), and DMAP (0.25 mmol, 31 mg) were employed to afford 43 mg of the desired product with 26% yield. ^1^H NMR (400 MHz, CDCl3) δ 8.58 (d, *J* = 1.7 Hz, 1H), 7.96–7.93 (m, 1H), 7.39 (d, *J* = 8.5 Hz, 1H), 7.34–7.28 (m, 2H), 7.18–7.14 (m, 1H), 7.01–6.96 (m, 2H), 6.91 (dd, *J* = 3.5:1.1 Hz, 1H), 6.82 (d, *J* = 4.0 Hz, 1H), 5.83 (d, *J* = 9.8 Hz, 1H), 5.44 (s, 1H), 3.67 (s, 3H), 3.52–3.40 (m, 2H), 2.98–2.78 (m, 2H), 2.62–2.53 (m, 1H), 2.42 (s, 3H), 2.40–2.30 (m, 1H), 2.08–1.98 (m, 1H), 1.92–1.84 (m, 1H), 1.80–1.58 (m, 3H), 1.54–1.40 (m, 4H), 1.35–1.25 (m, 4H), 0.98 (d, *J* = 6.0 Hz, 3H), 0.89 (d, *J* = 7.2 Hz, 3H) (Figure S28). ^13^C NMR (100 MHz, CDCl_3_) δ 197.9, 172.1, 147.7, 142.4, 139.3, 136.6, 132.8, 130.9, 130.6, 129.5, 128.0, 127.1, 125.3, 123.9, 122.9, 121.3, 120.6, 119.6, 110.5, 104.6, 92.7, 91.7, 80.3, 51.7, 45.5, 37.4, 36.4, 35.0, 34.3, 32.1, 31.3, 28.9, 26.2, 24.8, 22.2, 20.4, 12.3 (Figure S29). LC/MS-MS [M + Na]^+^ calc. for C^39^H^43^NO^7^SNa 692.2657: found 692.2675 (Figure S40).

#### Synthesis of (3*R*,5*aS*,6*R*,8*aS*,9*R*,12*R*,12*aR*)-3,6,9-Trimethyldecahydro-12*H*-3,12-epoxy [1,2]­dioxepino­[4,3-*i*]­isochromen-10-yl 4-(5-(5-bromo-1-methyl-2-phenyl-1*H*-indol-3-yl)­thiophen-2-yl)-4-oxobutanoate (**D4**)

4-(5-(5-Bromo-1-methyl-2-phenyl-1*H*-indol-3-yl)­thiophen-2-yl)-4-oxobutanoic acid (**4d**) (0.07 mmol, 33 mg), DCM (5 mL), dihydroartemisinin (0.10 mmol, 30 mg), EDCI (0.28 mmol, 54 mg), and DMAP (0.07 mmol, 9 mg) were employed to afford 40 mg of the desired product with 77% yield. ^1^H NMR (400 MHz, CDCl_3_) δ 8.08–8.06 (m, 1H), 7.57–7.54 (m, 1H), 7.48–7.44 (m, 3H), 7.39–7.32 (m, 1H), 7.23 (dd, *J* = 8.6:1.8 Hz, 1H), 6.77–6.73 (m, 1H), 5.75 (d, *J* = 9.8 Hz, 1H), 5.39 (s, 1H), 3.55 (s, 3H), 3.36–3.07 (m, 1H), 2.78–2.70 (m, 1H), 2.39–2.30 (m, 1H), 2.12–2.05 (m, 2H), 2.03–1.96 (m, 2H), 1.89–1.82 (m, 1H), 1.76–1.65 (m, 2H), 1.61–1.55 (m, 1H), 1.48–1.39 (m, 4H), 1.35–1.22 (m, 4H), 0.94–0.80 (m, 6H) (Figure S30). ^13^C NMR (100 MHz, CDCl_3_) δ 190.6, 171.9, 146.4, 140.8, 140.5, 136.0, 132.8, 131.1, 130.7, 129.7, 129.2, 127.8, 125.8, 125.6, 122.5, 114.7, 11.5, 107.8, 104.7, 92.3, 91.7, 80.4, 51.7, 45.4, 37.5, 36.4, 34.3, 33.3, 32.0, 31.3, 28.6, 26.2, 24.8, 22.2, 20.4, 12.3 (Figure S31). LC/TOF [M + Na]^+^ calc. for C_38_H_40_BrNO_7_SNa 756.1601: found 756.1575 (Figure S41).

#### Synthesis of (3*R*,5*aS*,6*R*,8*aS*,9*R*,12*R*,12*aR*)-3,6,9-Trimethyldecahydro-12*H*-3,12-epoxy­[1,2]­dioxepino­[4,3-i]­isochromen-10-yl 4-(5-(2-(4-methoxyphenyl)-1-methyl-1*H*-indol-3-yl)­thiophen-2-yl)-4-oxobutanoate (**D5**)

4-(5-(2-(4-Methoxyphenyl)-1-methyl-1*H*-indol-3-yl) thiophen-2-yl)-4-oxobutanoic acid (**4e**) (0.14 mmol, 60 mg), (5 mL) dihydroartemisinin (0.21 mmol, 61 mg), EDCI (0.57 mmol, 109 mg), and DMAP (0.14 mmol, 18 mg) were employed to afford 55 mg of the desired product with 56% yield. ^1^H NMR (400 MHz, CDCl_3_) δ 8.59 (d, *J* = 7.8 Hz, 1H), 7.99–7.92­(m 1H), 7.38 (d, *J* = 4.0 Hz, 1H), 7.33–7.28 (m, 2H), 7.18–7.14 (m, 1H), 7.02–6.92 (m, 4H), 5.83 (d, *J* = 9.9 Hz, 1H), 5.43 (s, 1H), 3.86 (s, 3H), 3.61­(s, 3H) 3.57–3.35 (m, 2H), 2.99–2.81 (m, 2H), 2.62–2.55 (m, 1H), 2.41–2.31 (m, 1H), 2.05–1.98 (m, 1H), 1.92–1.84 (m, 1H), 1.82–1.66 (m, 4H), 1.63–1.53 (m, 2H), 1.54–1.44 (m, 2H), 1.41 (s, 3H), 1.34–1.19 (m, 4H), 0.94 (d, *J* = 6.1 Hz, 3H), 0.84 (d, J = 7.1 Hz, 3H) (Figure S32). ^13^C NMR (100 MHz, CDCl_3_) δ 190.5, 171.9, 147.4, 140.0, 139.8, 137.3, 132.8, 131.3, 131.1, 129.5, 129.1, 126.1, 125.2, 123.0, 121.4, 120.1, 110.0, 108.2, 104.6, 92.3, 91.7, 80.3, 51.7, 45.4, 37.4, 36.4, 35.0, 34.3, 32.0, 31.1, 28.7, 26.2, 24.8, 22.2, 20.4, 12.3 (Figure S33). LC/MS-MS [M + Na]^+^ calc. for C_39_H_43_NO_8_SNa 708.2607: found 708.2627 (Figure S42).

### DPPH Assay

The radical scavenging capacities of the compounds were determined by modifying the DPPH method of Blois.^73^ The method was applied as stated in our previous study.^74^ %Radical scavenging activity (%RSA) was calculated as stated in “eq 1”.

1 $$\%\mathrm{RSA}=\frac{\left({\mathrm{blank}}_{\mathrm{Abs}}-{\mathrm{sample}}_{\mathrm{Abs}}\right)}{{\mathrm{blank}}_{\mathrm{Abs}}}\times 100$$

### ABTS Assay

The method of Re (1999) was used with modification.^75^ The method was applied as previously described.^74^ %RSA was calculated as specified in the DPPH method.

### Galvinoxyl Assay

Antioxidant capacities of the synthesized compounds were determined by modifying the galvinoxyl method of Shi and Niki.^76^ A concentration range of 13.15–210.5 μM was established for all samples, and 0.125–2 μM for Trolox. Galvinoxyl solution and the compounds were mixed in 0.5 mL tubes to achieve a galvinoxyl concentration of 8 μM. The tubes were incubated in the dark for 20 min. After incubation, the samples were transferred to a 96-well microplate and read at 428 nm with a microplate reader. %Radical scavenging activity was calculated as described in eq 1. The antioxidant potentials of the samples were compared with Trolox.

#### FRAP (Fe3+ Ion Reducing Power) Assay

The Benzie and Strain (1996) method was modified and applied.^77^ Acetate buffer (0.3 M, pH: 3.60), TPTZ (0.01 M), and FeCl_3_ (0.02 M) solutions were mixed in a 10:1:1 ratio and incubated at 37 °C for 30 min. A concentration range of 37.5–600 μM was established for all samples, and 6.25–100 μM for Trolox. Three samples were prepared for each concentration and blank. The compound and FRAP solution were mixed in a 1:2 ratio in a 96-well microplate and incubated in a heat block (37 °C) for 4 min. After incubation, samples were read at 593 nm with a microplate reader. The values obtained were compared with Trolox, which was used as a standard.

#### Cell Culture and MTT Assay

Pancreatic (PANC-1), human breast (MDA-MB-231), colon (HT-29), liver (HepG2), endometrial (Ishikawa) cancer cell lines, and a healthy endothelial (HUVEC) cell line were used in this study. The cell lines were purchased from the European Cell Culture Collection (ECACCCC). These cell lines were grown in DMEM or RPMI medium containing 1% penicillin/streptomycin and 10% fetal bovine serum (FBS) in an incubator at 37 °C, 5% CO_2_, and 95% humidity.

The cytotoxic properties of the tested derivatives on cells were determined by using the MTT assay. The tested compounds were dissolved in dimethyl sulfoxide (DMSO), and dilutions were made with DMSO.

The tested compounds were applied at final concentrations of 7.8, 15.6, 31.25, 62.5, and 125 μM. Each concentration was evaluated using three technical replicates. The final DMSO concentration in all treatment wells did not exceed 0.5%, and vehicle control wells contained the same final DMSO concentration as the corresponding treatment wells. The method was performed as described below. 96-well microplates were seeded with 10^3^ cells/well and 100 μL of medium. After 24 h of incubation, the culture was refreshed with 200 μL of fresh medium and treated with various concentrations of the desired compound, and left in the incubator for 24 h. DMSO was added to the controls. After 24 h of incubation, the medium was carefully removed, and 100 μL of fresh medium and 10 μL of MTT solution were added. The medium was incubated at 37 °C for 4 h.

The medium was then removed, 50 μL of DMSO was added, and the plate was incubated for 30 min at 37 °C to dissolve the formazan crystals that had precipitated from the bottom of the plate. After the formazan crystals were dissolved, absorbance values were measured at 590 nm using a microplate reader. Cell viability was calculated relative to the vehicle control. EC_50_ values were calculated using exponential trendline curves generated in Microsoft Excel from the concentration–response data. When cell viability did not decrease below 50% at the highest tested concentration, the EC_50_ value was reported as >125 μM.

### Determination of Invasion Capacity

To determine the effects of cytotoxic compounds on cell invasiveness, experiments were performed in 24-well cell culture plates using “Transwell Matrigel Invasion Chamber” dishes. The invasive properties of the compounds on PANC-1, HepG2, and HT-29 cells were investigated. Before the experiment, the Matrigel wells were removed from the −20 °C refrigerator and placed in the 24-well plate. 0.5 mL of serum-free medium was added to the Matrigel well and to the chamber where the 0.5 mL well was placed. After incubation at 37 °C for 2 h, the medium was removed.

Then, 10^4^ cells were seeded in the lower chamber of each invasion well with 0.750 mL of serum-containing medium and in the upper chamber with 0.5 mL of serum-free medium. The EC_50_ doses of the tested compounds were applied to the plates and incubated at 37 °C for 24 h. After 24 h of incubation, the medium was removed from the plate. Cells that remained on the upper surface of the Matrigel membrane without invasion were removed. To fix cells that had invaded and migrated to the lower surface of the membrane, methanol was added to the 0.5 mL Matrigel well and to the chamber where the 0.5 mL well was placed. The plate was incubated at −20 °C for 10 min.

The methanol was then removed, and 0.5 mL of crystal violet dye was added to the Matrigel well and the chamber where the 0.5 mL well was placed. The dye was then removed from the invasion well and washed with phosphate-buffered saline (PBS), after which it was photographed under a light microscope.^78^

Cell counts were then performed using the ImageJ program. The formula in eq 2 was used to calculate the %invasion value.

2 $$\mathrm{\% invasion}=\frac{n\mathrm{umber of cells in the}\;M\mathrm{atrigel matrix basement membrane}}{n\mathrm{umber of cells in the control membrane}}\times 100$$

### Determination of Apoptosis

Apoptotic properties of the compounds used in this study were determined using the Annexin V-FITC/PI Apoptosis kit (ABP Biosciences) and in accordance with the manufacturer’s instructions.

For this purpose, cells were seeded in 6-well plates at 30 × 10^3^ cells/well in 3 mL of medium and incubated for 24 h. After 24 h, EC_50_ doses of the compounds and 200 μM concentration of hydrogen peroxide (H_2_O_2_), used as a positive control, were applied and incubated for 24 h. After incubation, the medium was removed, and the cells were lifted using Trypsin-EDTA. After, the cells were centrifuged at 2000 rpm for 5 min. Annexin V/PI staining was then applied as specified in the manufacturer’s protocol. The percentages of viable, apoptotic, and necrotic cells were obtained using a flow cytometry device (CytoFLEX, Beckman Coulter). Flow cytometry data were first evaluated using an FSC-A versus SSC-A plot, and the main cell population was selected within the P1 gate to exclude debris and events outside the target cell population. Events within the P1 gate were subsequently analyzed using FITC-A versus PE-A plots. Annexin V–/PI– cells were classified as viable, Annexin V+/PI– cells as early apoptotic, Annexin V+/PI+ cells as late apoptotic/secondary necrotic, and Annexin V–/PI+ cells as necrotic. Total apoptosis was calculated as the sum of the early and late apoptotic populations. Representative plots are provided in the Supporting Information (Figures S43 and S44).

### Determination of mRNA Expression of Genes Involved in Apoptosis

30 × 10^3^ cells were seeded into each well and incubated in the incubator for 24 h. After 24 h, the EC_50_ doses of the compounds were applied and incubated for 24 h. After incubation, the Analytik Jena innuPREP RNA Mini Kit was used to isolate total RNA, and the manufacturer’s recommended kit procedure was followed. The ABM OneScript cDNA Synthesis Kit was used to synthesize cDNA, following the manufacturer’s recommended procedure.

Real-time PCR was performed in 96-well microplates using the KiloGreen MasterMix kit from ABM. For the reaction, 7.5 μL of a mixture containing 5 μL of KiloGreen fluorescent dye, 0.5 μL of forward primer, 0.5 μL of reverse primer, and 1.5 μL of RNase-free sterile water was added to each well. 2.5 μL of cDNA diluted 1:1 was then added to each well.

In this study, primer sequences for BAX, P53, BCL2, Casp3, Casp8, Casp9, and GAPDH were used to determine apoptosis-related gene expression (Table 4). The GAPDH gene was selected as the control gene (housekeeping) in the study, and the data were normalized using the GAPDH gene. Amplification was performed by placing the plate on a Biosystems StepOnePlusqPCR (Thermo, USA) device. The 2^–ΔΔCT^ method was used to determine changes in mRNA expression. Additionally, primer melt curves and numerical gene expression data were added to the Supporting Information (Figures S46–S56).

**Table 4:** Forward (F) and Reverse (R) Primer Sequences Used in the Study

gene	sequences of the primers
GAPDH	F: GTCTCCTCTGACTTCAACAGCG
	R: ACCACCCTGTTGCTGTAGCCAA
P53	F: CCTCAGCATCTTATCCGAGTGG
	R: GGATGGTGGTACAGTCAGAGC
BAX	F: TCAGGATGCGTCCACCAAGAAG
	R: TGTGTCCACGGCGGCAATCATC
BCL2	F: ATCGCCCTGTGGATGACTGAGT
	R: GCCAGGAGAAATCAAACAGAGGC
CASP3	F: GGAAGCGAATCAATGGACTCTGG
	R: GCATCGACATCTGTACCAGACC
CASP8	F: AGAAGAGGGTCATCCTGGGAGA
	R: TCAGGACTTCCTTCAAGGCTGC
CASP9	F: GTTTGAGGACCTTCGACCAGCT
	R: CAACGTACCAGGAGCCACTCTT

### Statistical Analysis

The data obtained from the study were expressed as mean ± standard deviation. One-way analysis of variance (one-way ANOVA) followed by Dunnett’s multiple comparisons post hoc test was used to compare statistically significant differences between the control and treated groups. Statistical calculations were performed using GraphPad Prism v.9.0 software. Statistical significance was defined as *p* < 0.05.

The data underlying this study are available in the published article and its online Supporting Information.

## Supplementary Material



## Notes

Spectral characterization data (^1^H NMR, ^13^C NMR, HRMS) for all compounds, additional qPCR results of the cell line (PDF)

## Abbreviations

DMSO: dimethyl sulfoxide
FDA: food and drug administration
H_2_O_2_: hydrogen peroxide
HAT: hydrogen atom transfer
HCC: hepatocellular carcinoma
I3C: indole-3-carbinol
LC-MS/MS: high-resolution mass spectra
ROS: reactive oxygen species
SET: single electron transfer
TLC: thin-layer chromatography
TMS: tetramethyl silane
