# Supplementary material for: Synthesis and Biological Evaluation of Thiophene-Bridged Indole–Artesunate Hybrids as Selective Anticancer Agents
Source: ACS Omega. 2026 Jul 24;11(31):46670–84. doi: 10.1021/acsomega.6c04482 (PMC13470901; doi:10.1021/acsomega.6c04482)
Supplement: Supplementary file 1 [file ao6c04482_si_001.pdf]

Supporting Information

Synthesis and Biological Evaluation of Thiophene-Bridged Indole–Artesunate Hybrids as Selective Anticancer Agents

Emrah Kavak, [§ * a] **Doğan Çetin,** [§ * a b] **Metin Konuş** [b], **Arif Kivrak** [c]

[a] Necmettin Erbakan University, BITAM-Science and Technology Research and Application Center, Konya, 42090, Turkey;

[b] Molecular Biology and Genetics Faculty of Sciences, Van Yüzüncü Yil University, Van, 65080, Turkey

[c] Chemistry, Faculty of Science Eşkisehir Osmangazi University, Eskişehir, 26040, Turkey

[§] **E.K** and **D.C**·contributed equally to this work**.**

TABLE OF CONTENTS

Figure S1 . ^{1}H NMR spectrum of 3a. 4
Figure S2. ^{13}C NMR spectrum of 3a. 4
Figure S3. ^{1}H NMR spectrum of 3b. 5
Figure S4. ^{13}C NMR spectrum of 3b. 5
Figure S5. ^{1}H NMR spectrum of 3c. 6
Figure S6. ^{13}C NMR spectrum 3c. 6
Figure S7. ^{1}H NMR spectrum of 3d. 7
Figure S8. ^{13}C NMR spectrum of 3d. 7
Figure S9. ^{1}H NMR spectrum of a. 8
Figure S10. ^{1}H NMR spectrum of b. 8
Figure S11. ^{13}C NMR spectrum of b. 9
Figure S12. ^{1}H NMR spectrum of 3e. 9
Figure S13. ^{13}C NMR spectrum of 3e. 10
Figure S14. ^{1}H NMR spectrum of 4a 11
Figure S15. ^{13}C NMR spectrum of 4a. 11
Figure S16. ^{1}H NMR spectrum of 4b. 12
Figure S17. ^{13}C NMR spectrum of 4b. 12
Figure S18. ^{1}H NMR spectrum of 4c. 13
Figure S19. ^{13}C NMR spectrum of 4c. 13
Figure S20. ^{1}H NMR spectrum of 4d. 14
Figure S21. ^{13}C NMR spectrum of 4d. 14
Figure S22. ^{1}H NMR spectrum of 4e. 15
Figure S23. ^{13}C NMR spectrum of 4e. 15
Figure S24. ^{1}H NMR spectrum of D1. 16
Figure S25. ^{13}C NMR spectrum of D1. 16
Figure S26. ^{1}H NMR spectrum of D2. 17
Figure S27. ^{13}C NMR spectrum of D2. 17

Figure S28. ^{1}H NMR spectrum of D3. 18
Figure S29. ^{13}C NMR spectrum of D3. 18
Figure S30. ^{1}H NMR spectrum of D4. 19
Figure S31. ^{13}C NMR spectrum of D4. 19
Figure S32. ^{1}H NMR spectrum of D5. 20
Figure S33. ^{13}C NMR spectrum of D5. 20
Figure S34. LC MS/MS Spectrum of 4a. 21
Figure S35. LC MS/MS Spectrum of 4b. 21
Figure S36. LC MS/MS Spectrum of 4c. 22
Figure S37. LC MS/MS Spectrum of 4e. 22
Figure S38. LC MS/MS Spectrum of D1. 23
Figure S39. LC MS/MS Spectrum of D2. 23
Figure S40. LC MS/MS Spectrum of D3. 24
Figure S41. LC MS/MS Spectrum of D4. 24
Figure S42. LC MS/MS Spectrum of D5. 25
Figure S43. The main cell population was identified using an FSC-A versus SSC-A plot and selected within the P1 gate. 25
Figure S44. Events within P1 were analyzed using FITC-A versus PE-A fluorescence.. 26
Figure S45. Melt curve of BAX. 30
Figure S46. Melt curve of P53. 30
Figure S47. Melt curve of Bcl-2. 31
Figure S48. Melt curve of Caspase-3. 31
Figure S49. Melt curve of Caspase-8. 32
Figure S50. Melt curve of Caspase-9. 32
Figure S51. Melt curve of GAPDH. 33
Table S1. qPCR results for HepG2 cell line ....**Error! Bookmark not defined.**
Table S2. qPCR results for Ishikawa cell line ....**Error! Bookmark not defined.**
Table S3. qPCR results for MDA-MB-231 cell line .**Error! Bookmark not defined.**
Table S4. qPCR results for HT-29 cell line ....**Error! Bookmark not defined.**
Table S5. Table S5. qPCR results for Panc-1 cell line**Error! Bookmark not defined.**

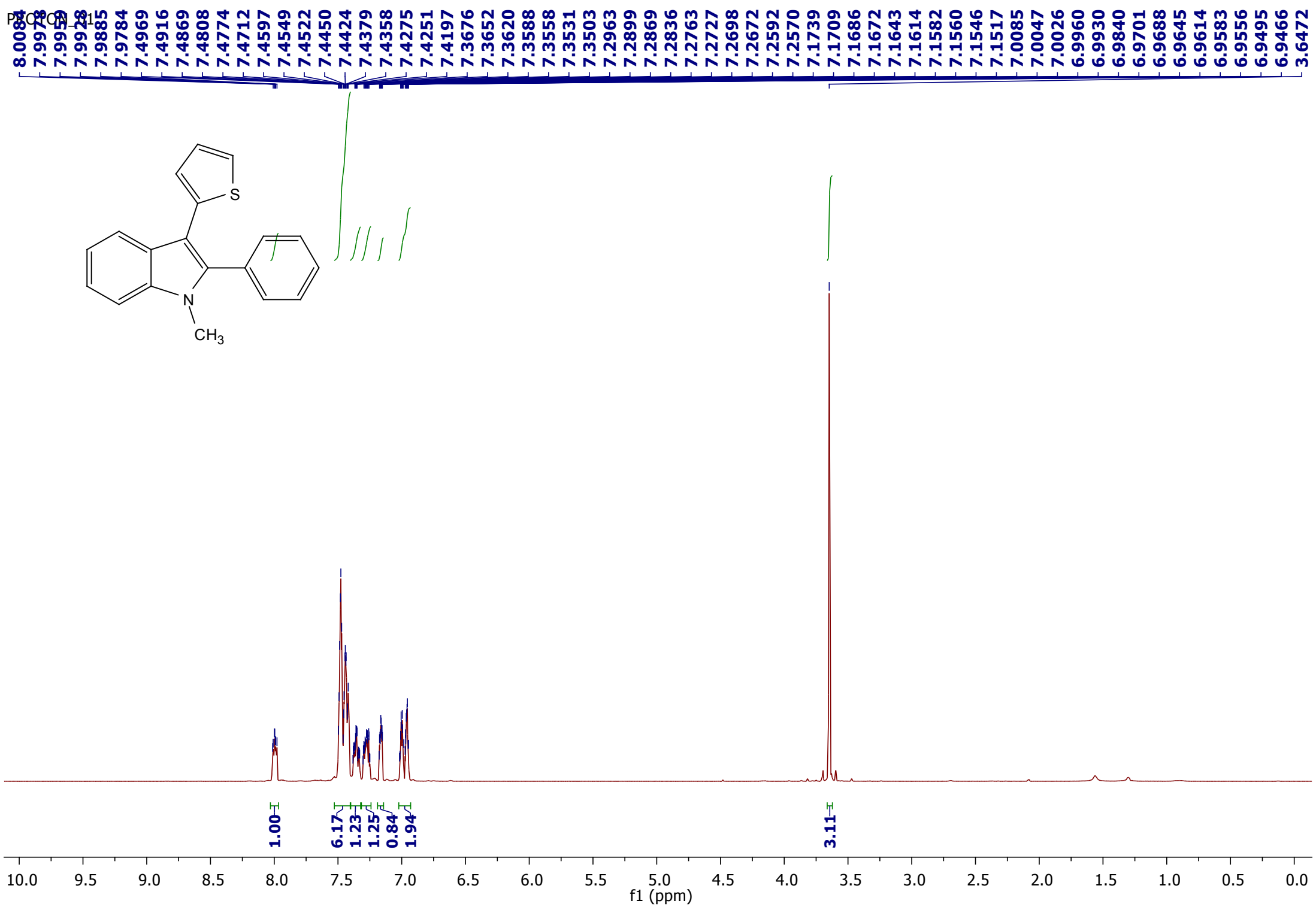

Figure S1 . ^{1}H NMR spectrum of 3a.

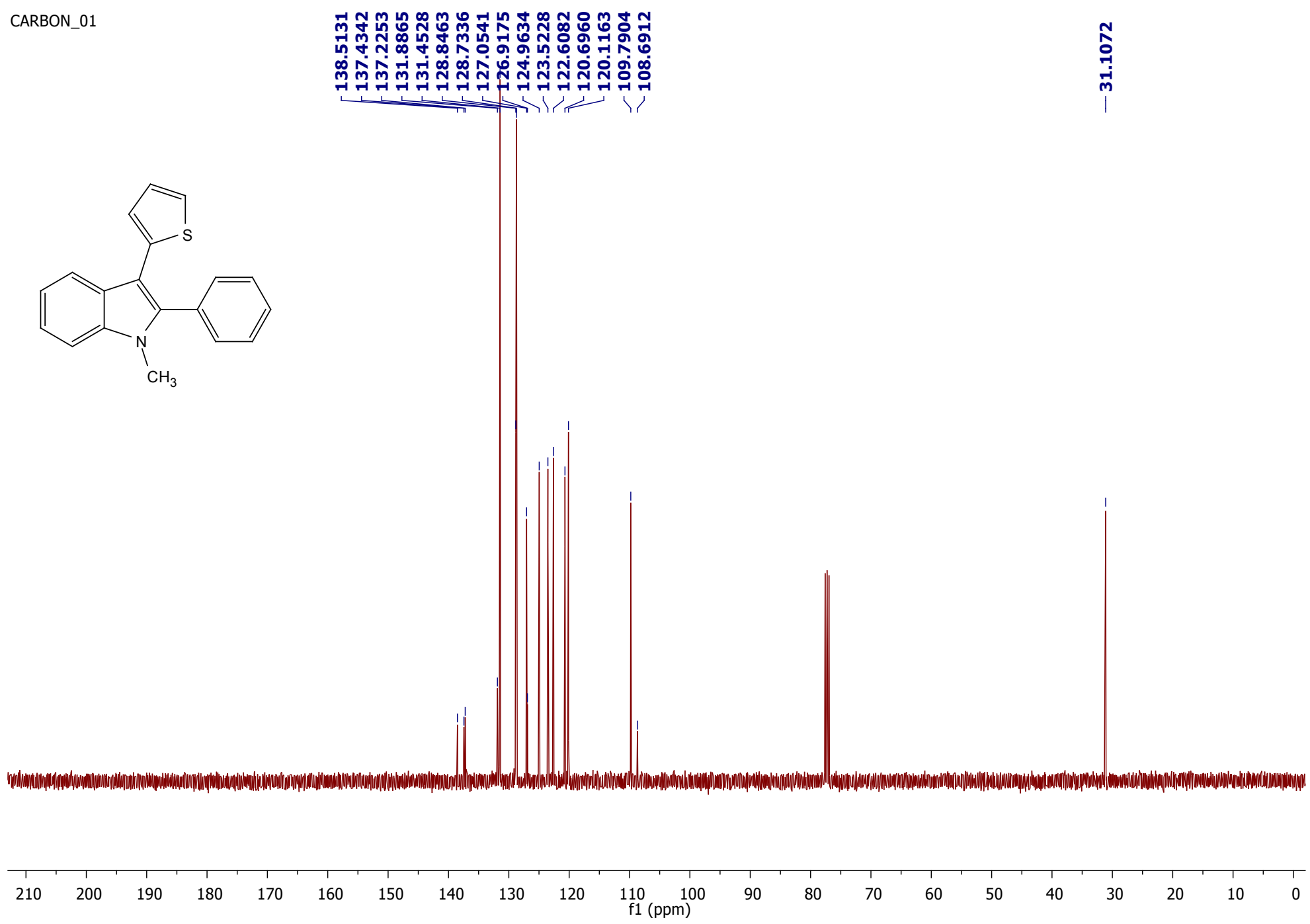

Figure S2. ^{13}C NMR spectrum of 3a.

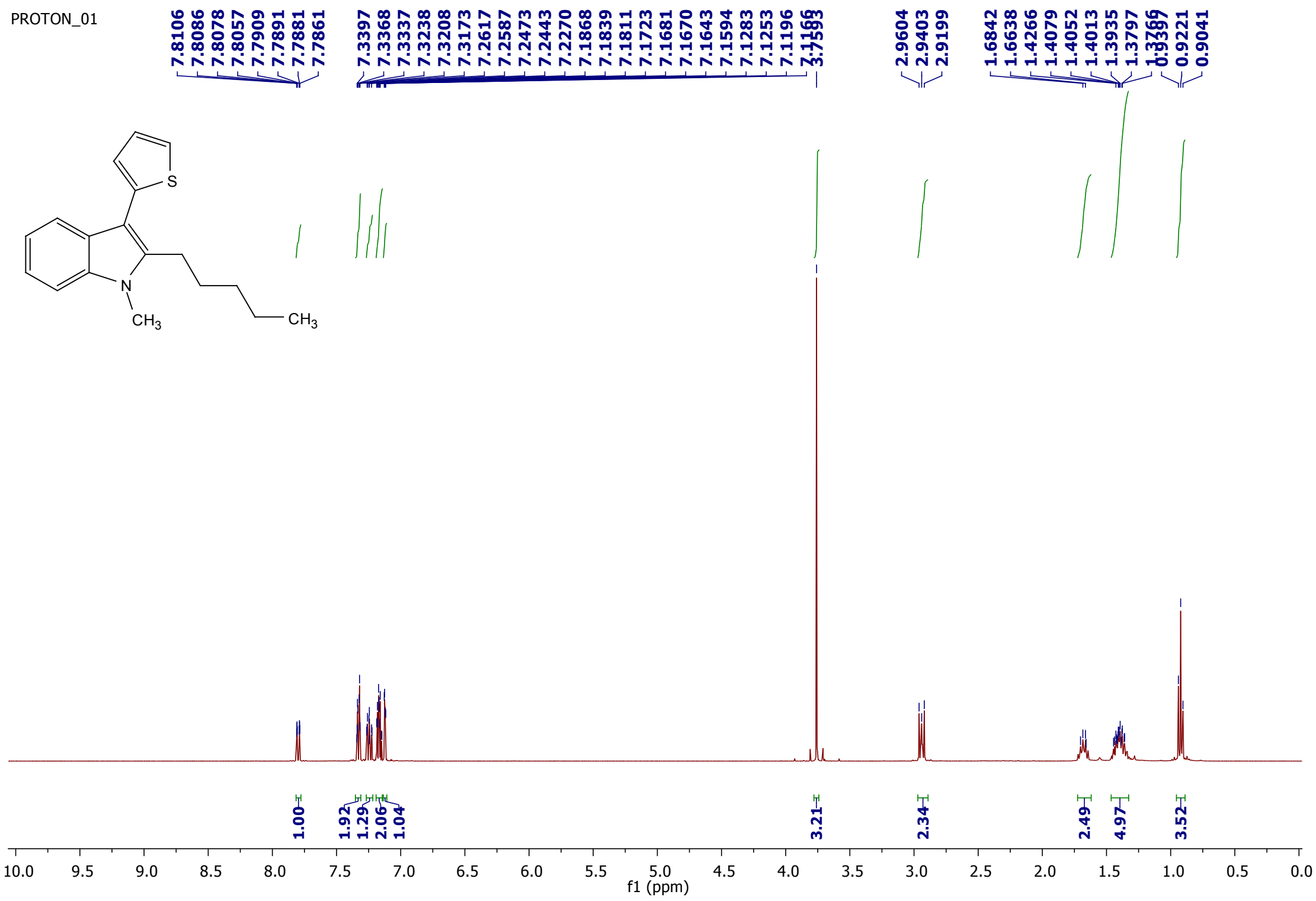

Figure S3. ^{1}H NMR spectrum of 3b.

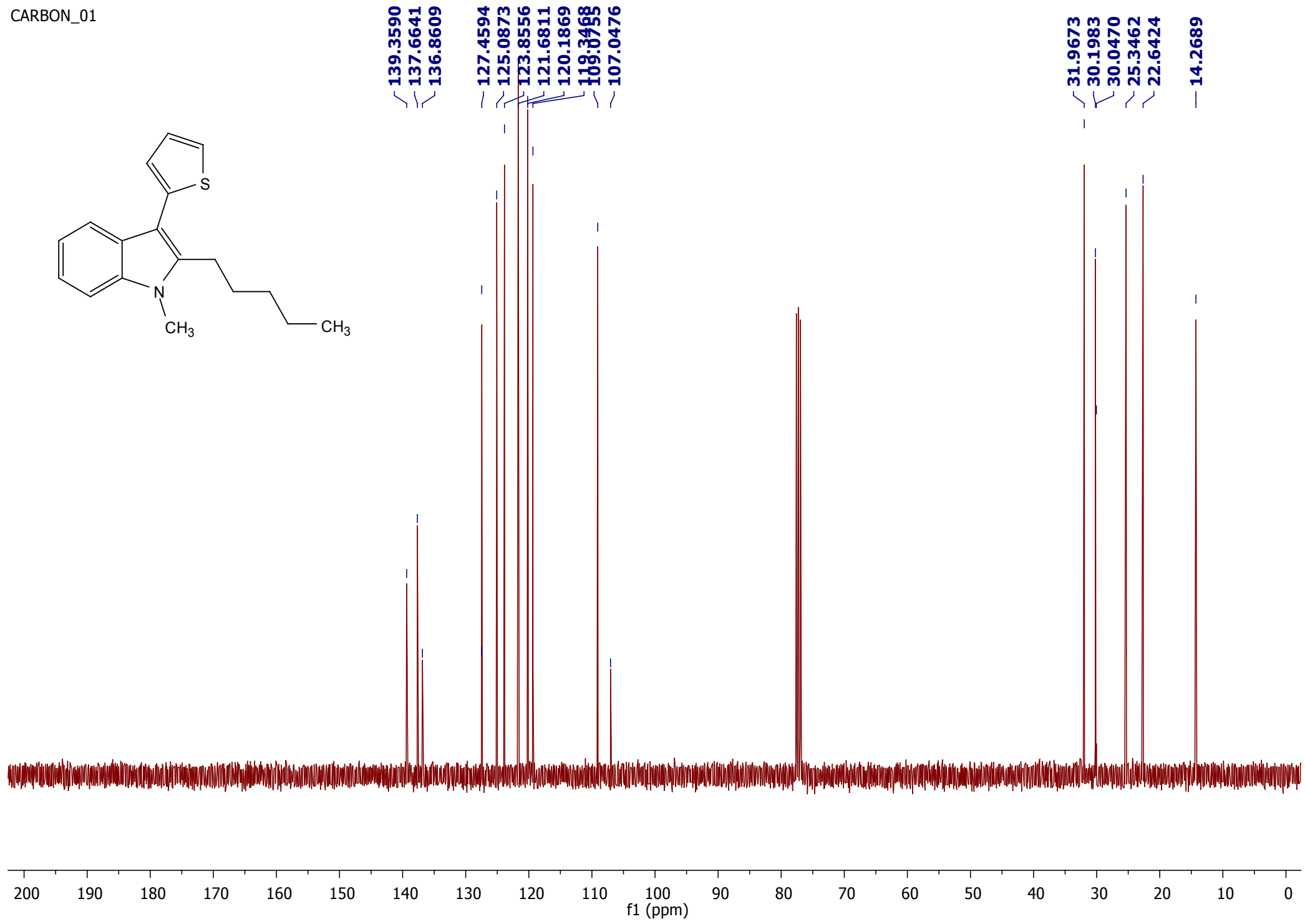

Figure S4. ^{13}C NMR spectrum of 3b.

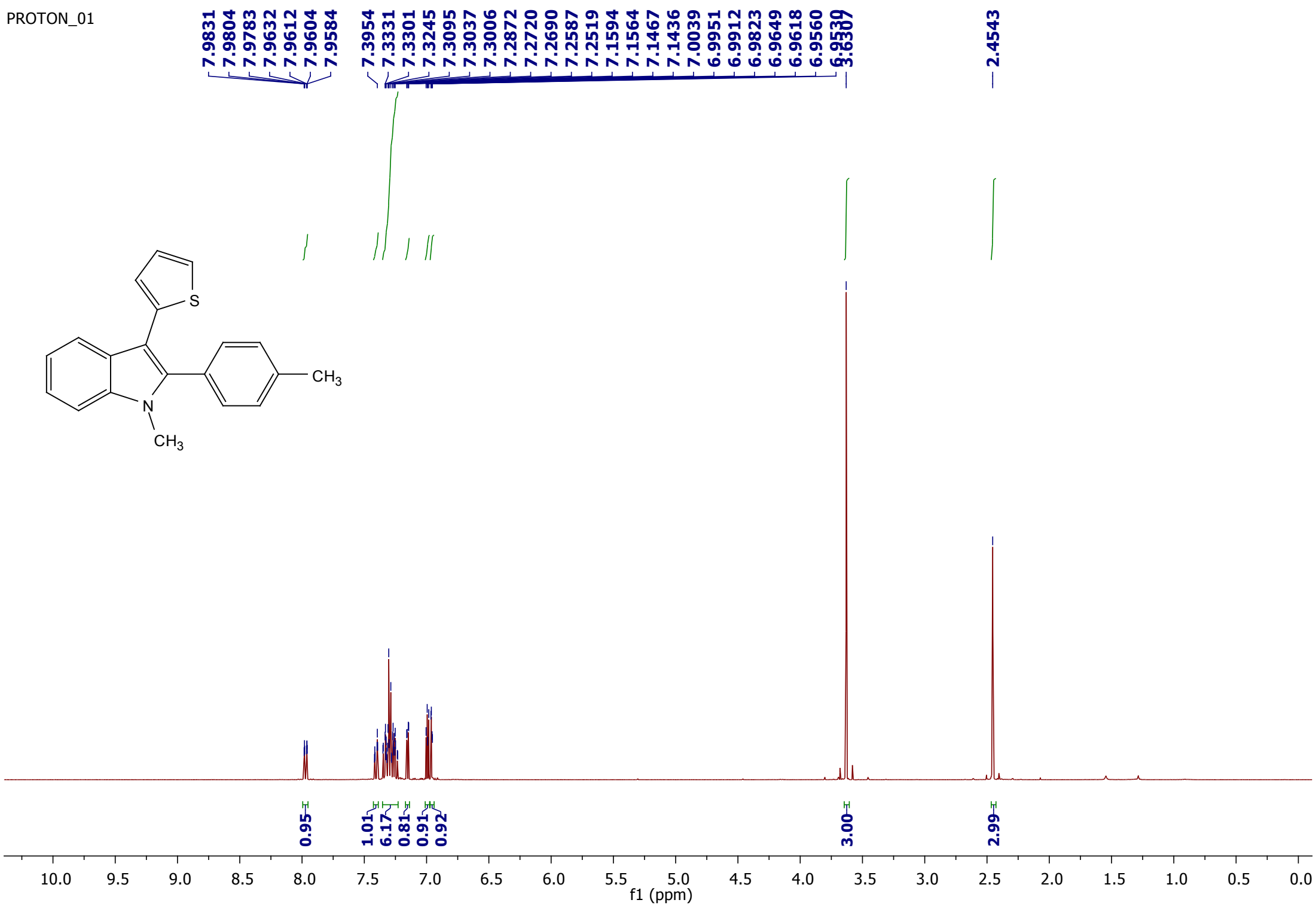

Figure S5. ^{1}H NMR spectrum of 3c.

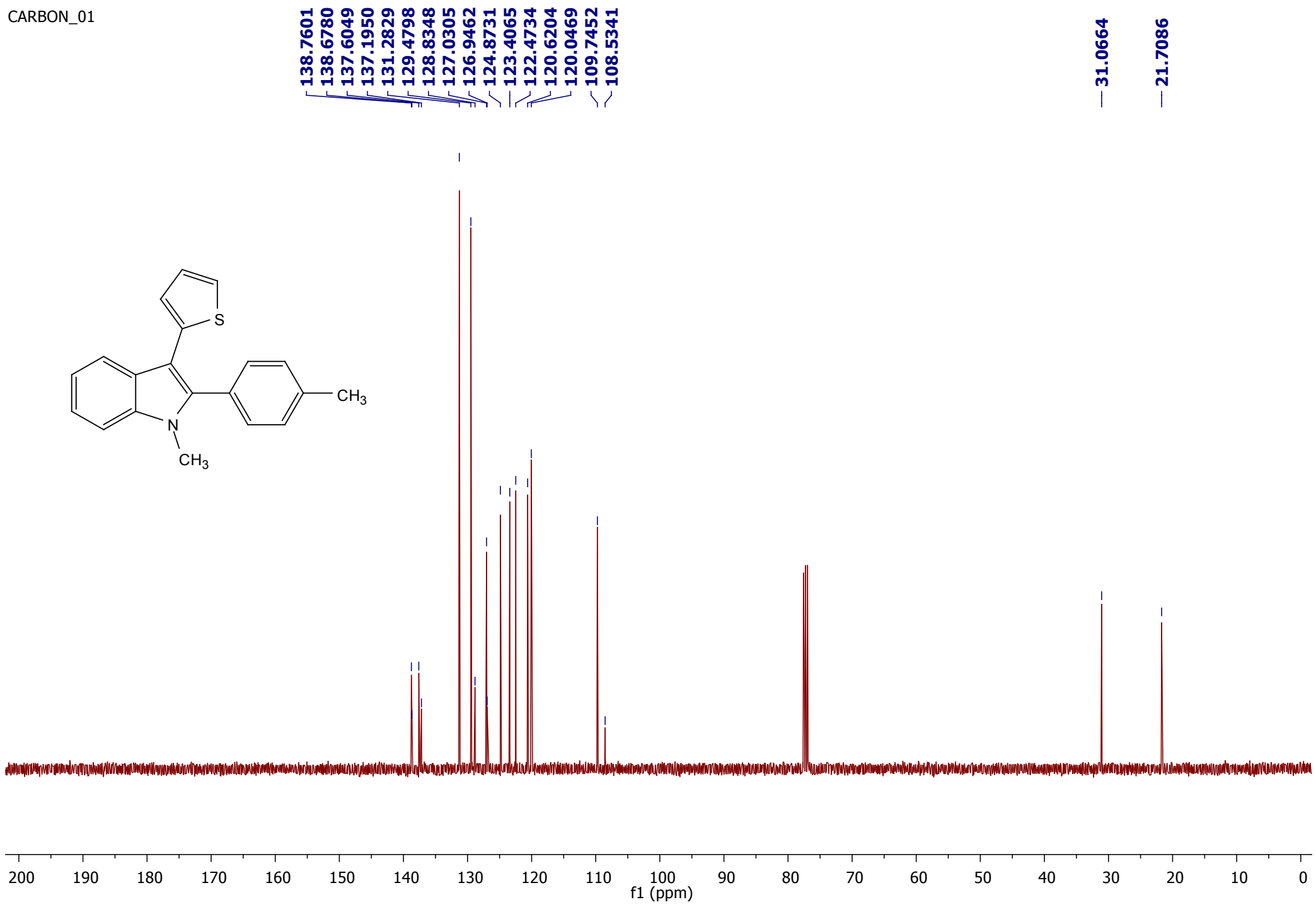

Figure S6. ^{13}C NMR spectrum 3c.

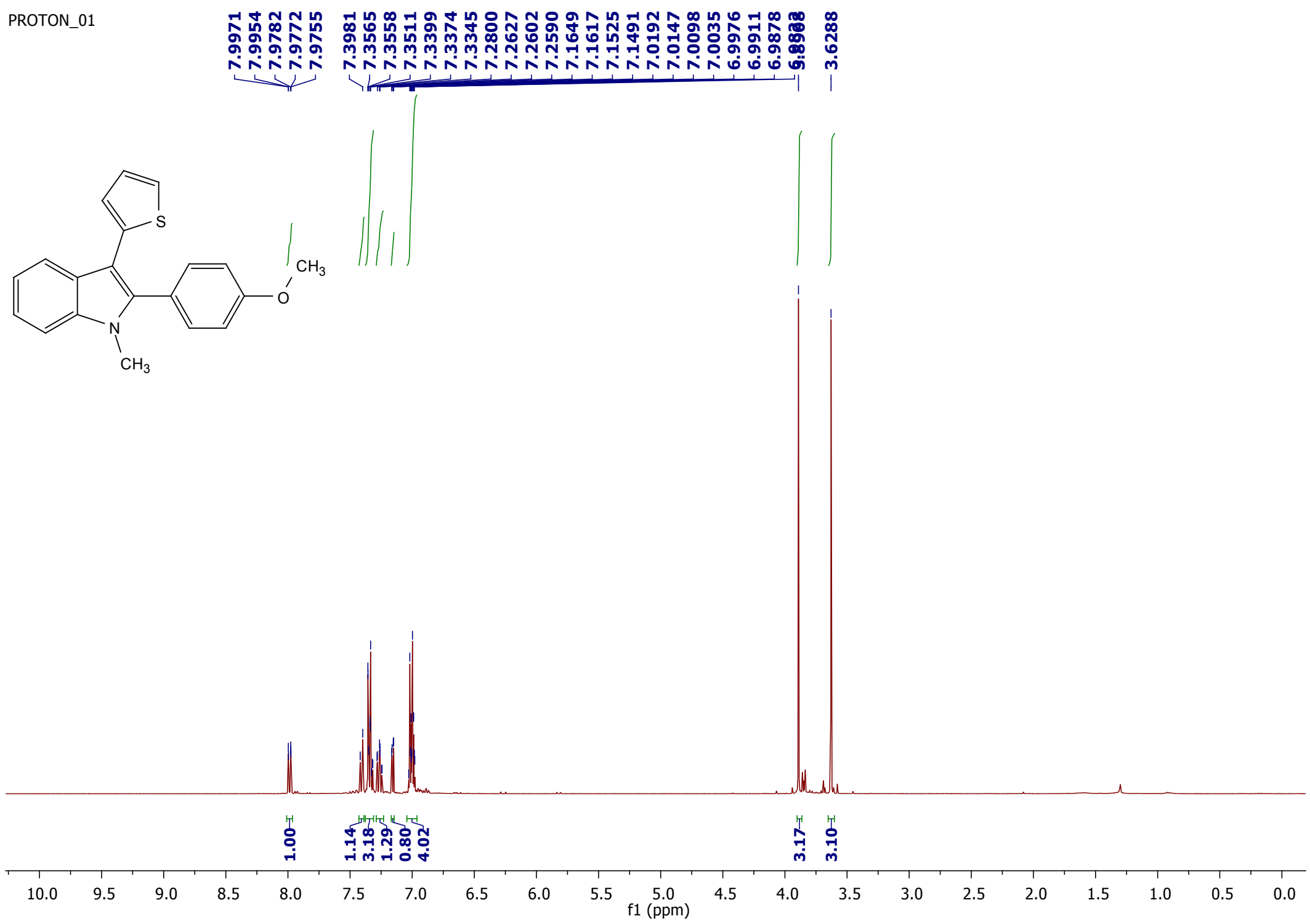

Figure S7. ^{1}H NMR spectrum of 3d.

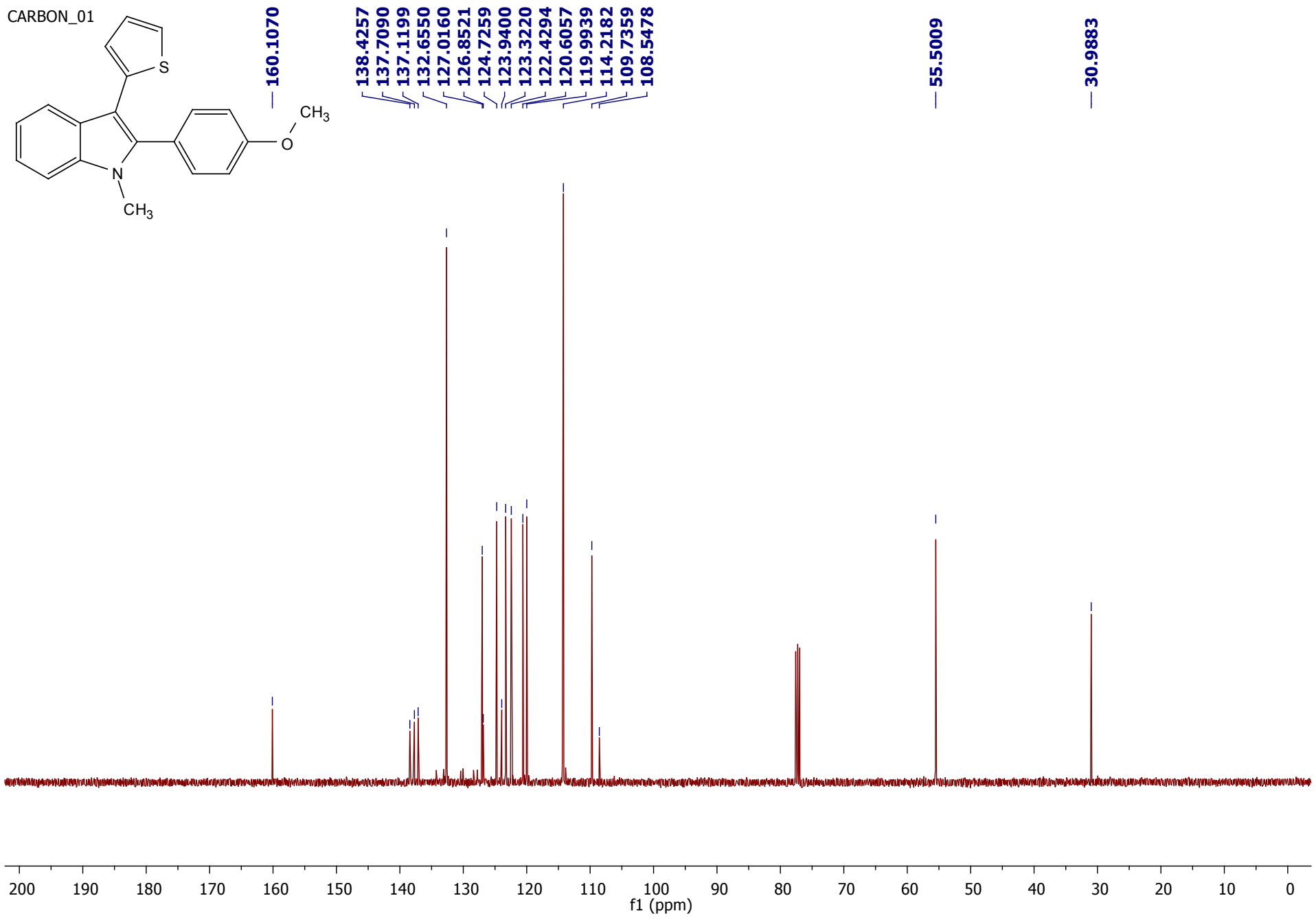

Figure S8. ^{13}C NMR spectrum of 3d.

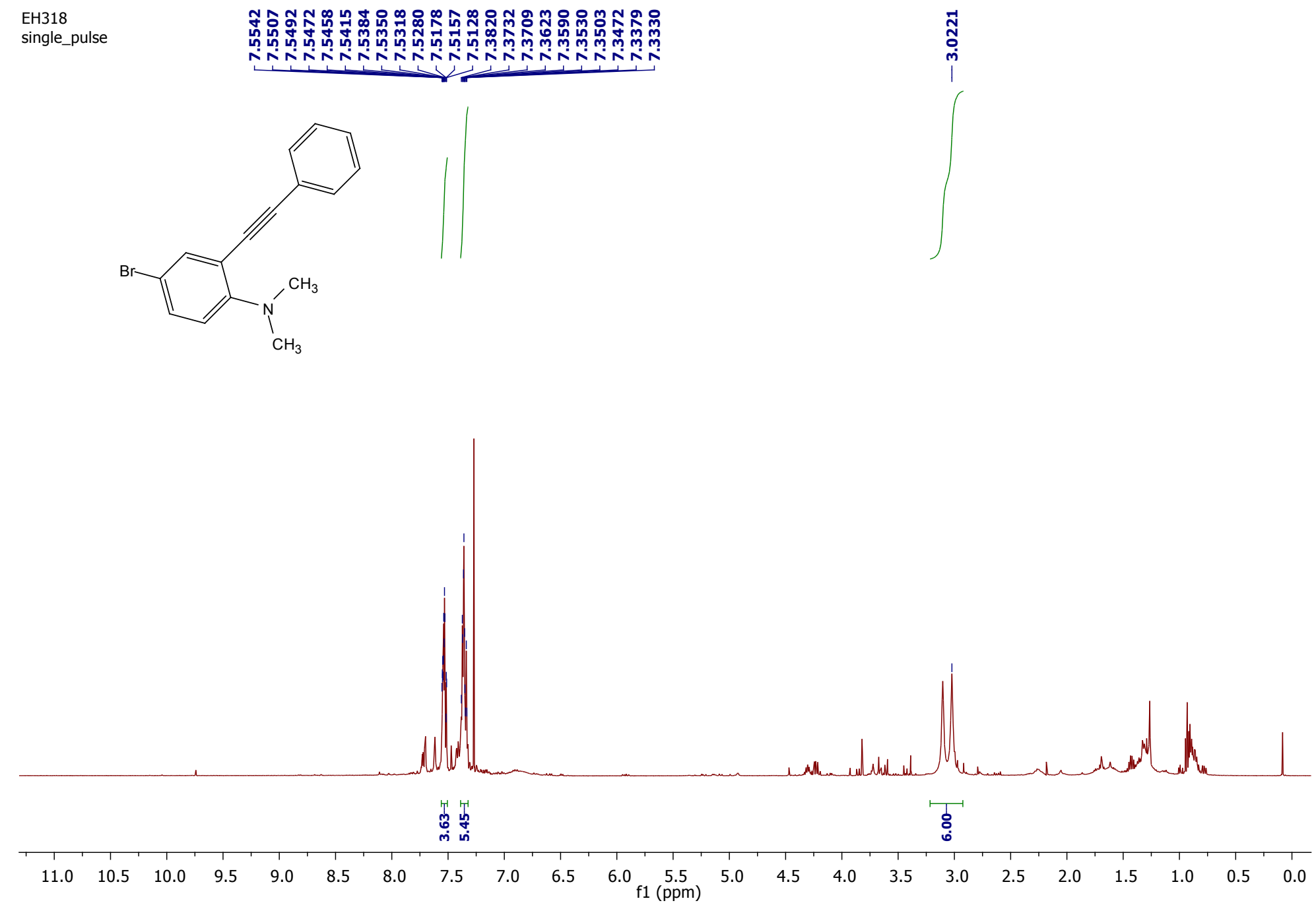

Figure S9. ^{1}H NMR spectrum of a.

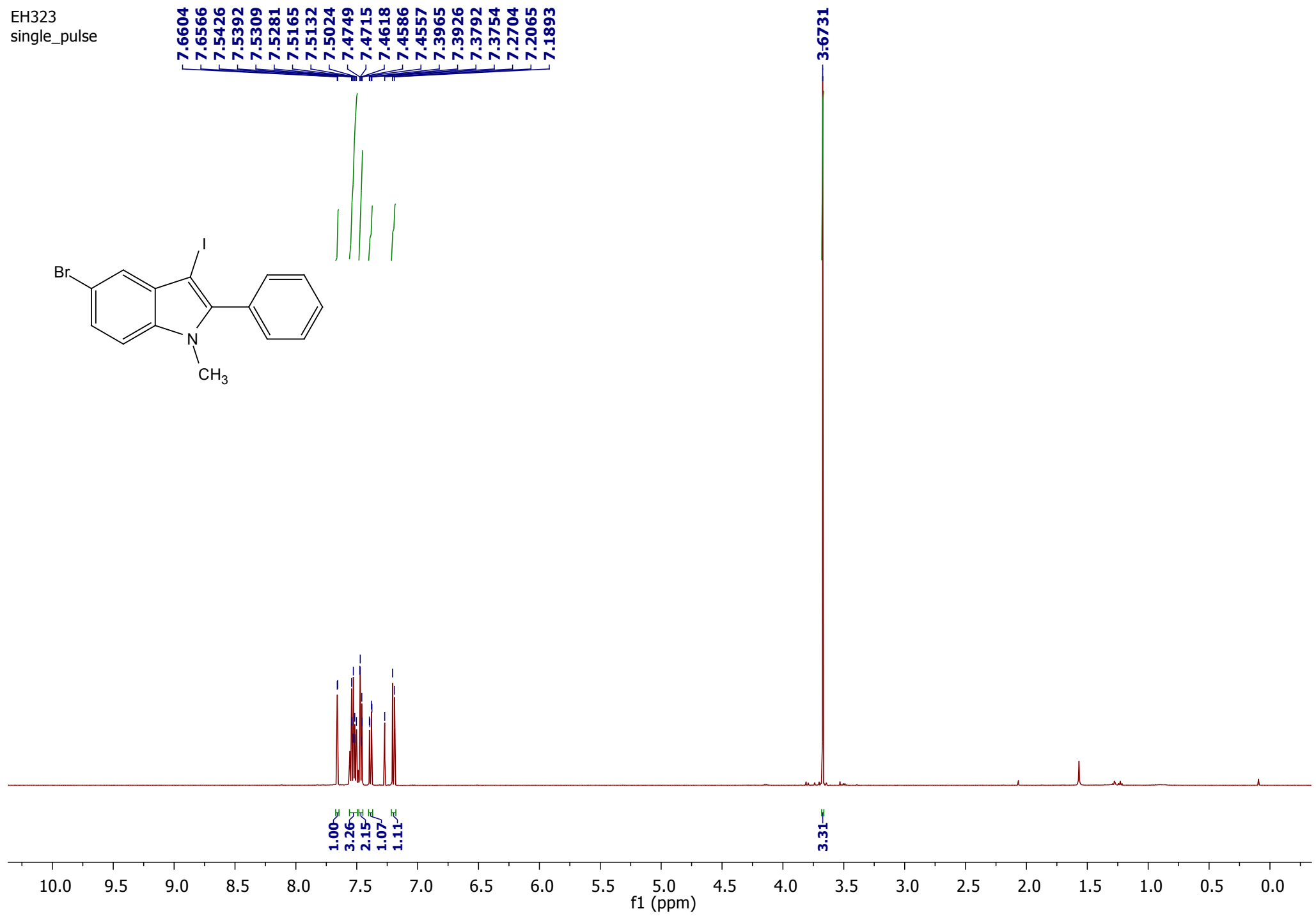

Figure S10. ^{1}H NMR spectrum of b.

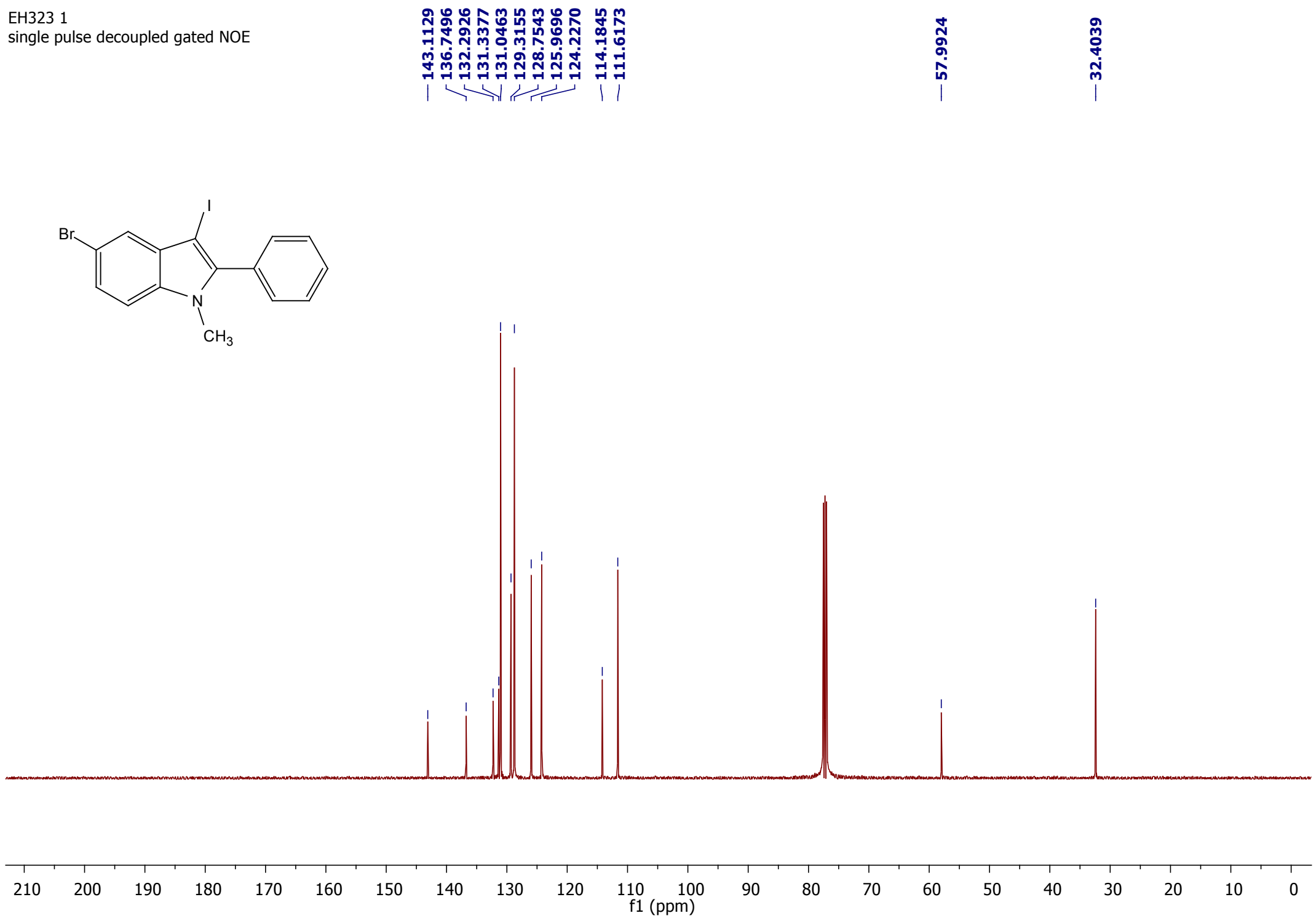

Figure S11. ^{13}C NMR spectrum of b.

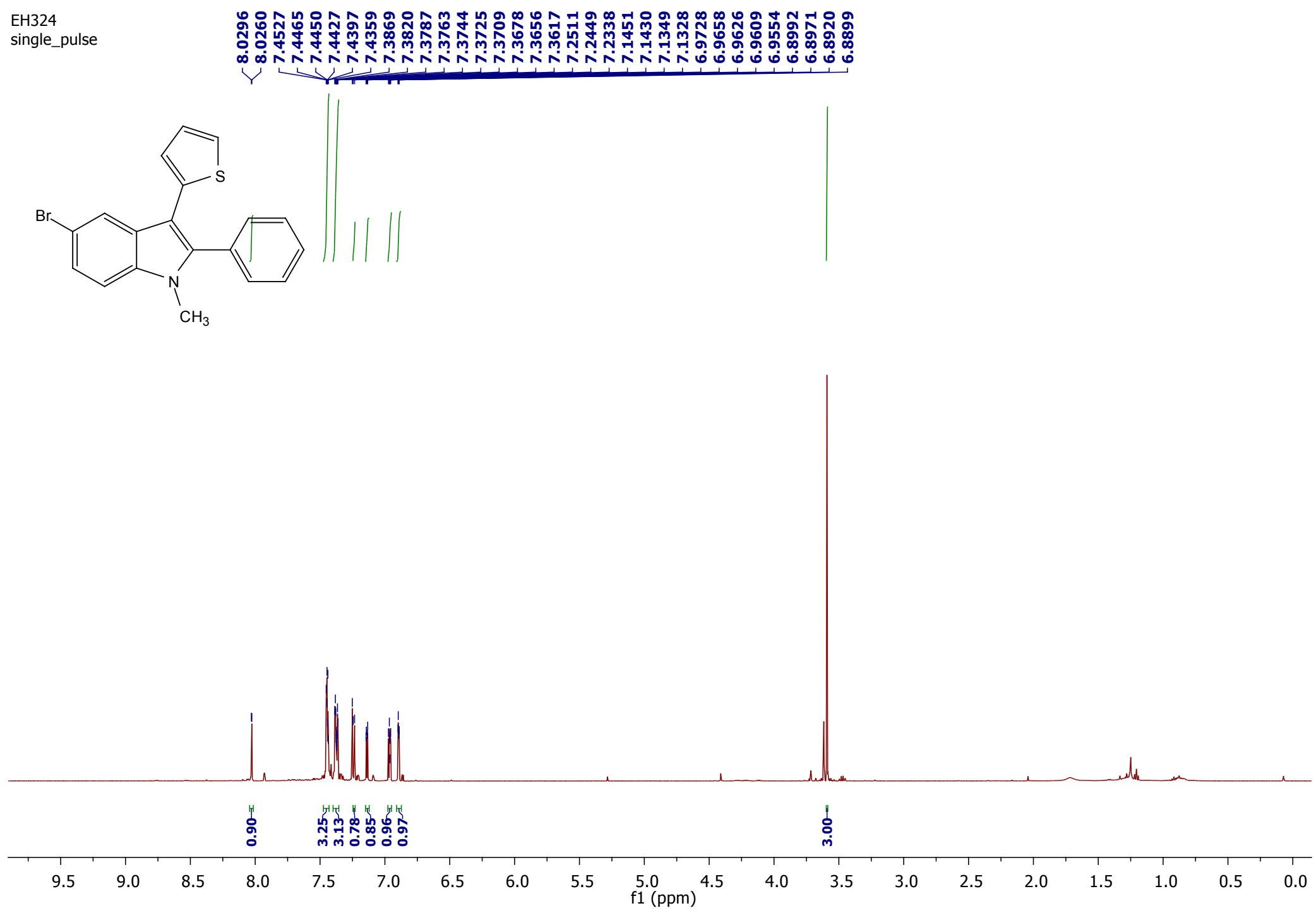

Figure S12. ^{1}H NMR spectrum of 3e.

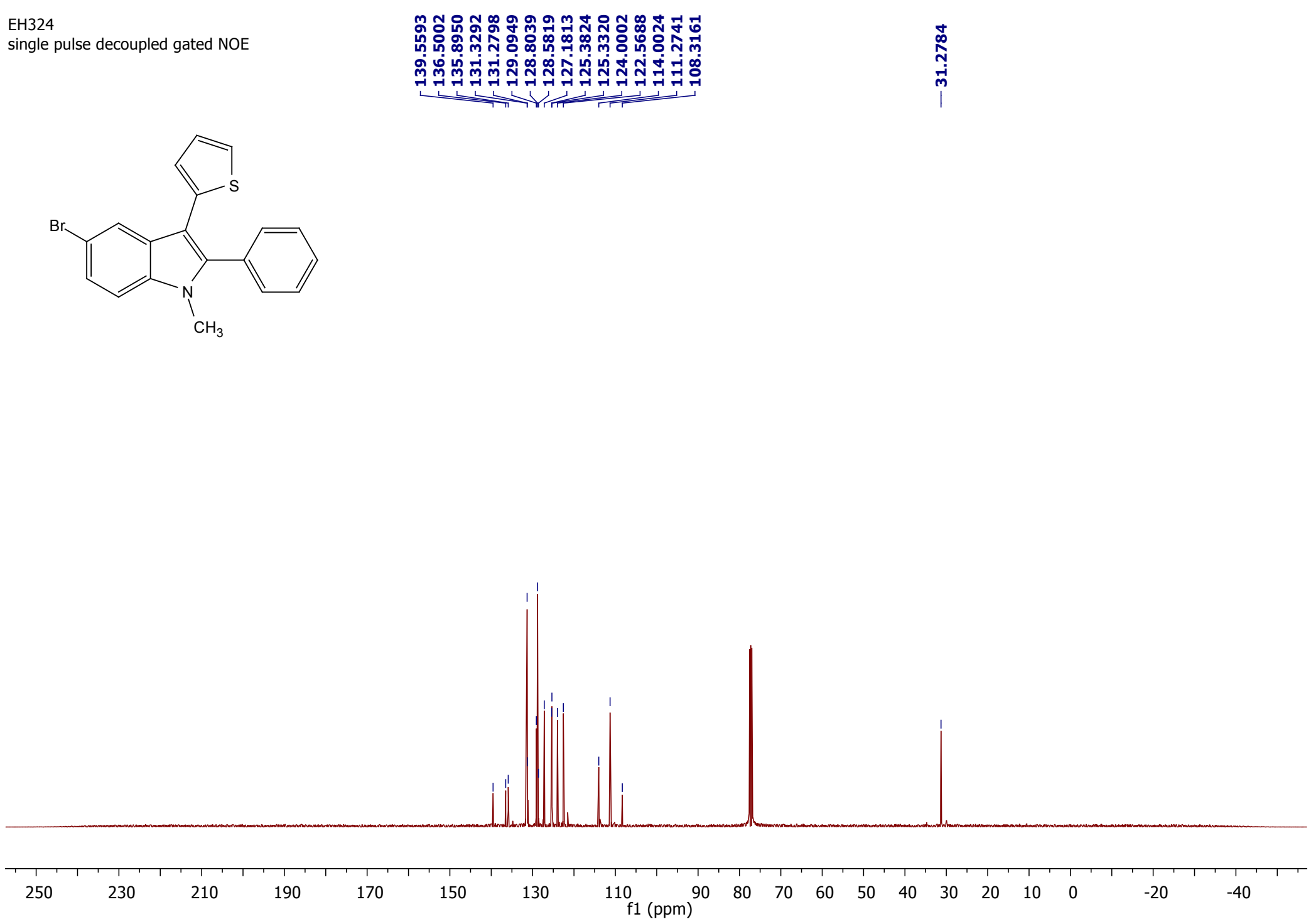

Figure S13. ^{13}C NMR spectrum of 3e.

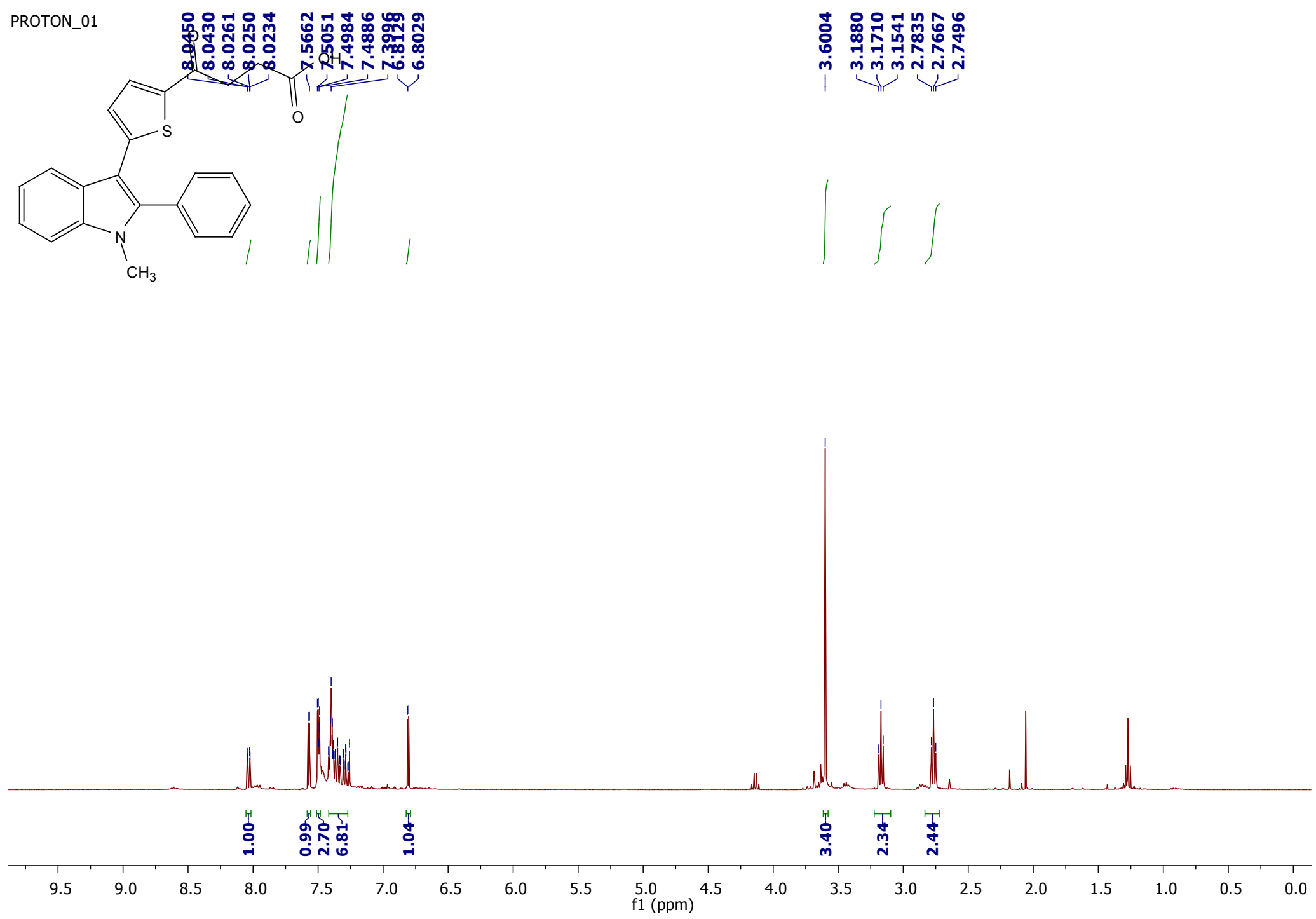

Figure S14. ^{1}H NMR spectrum of 4a

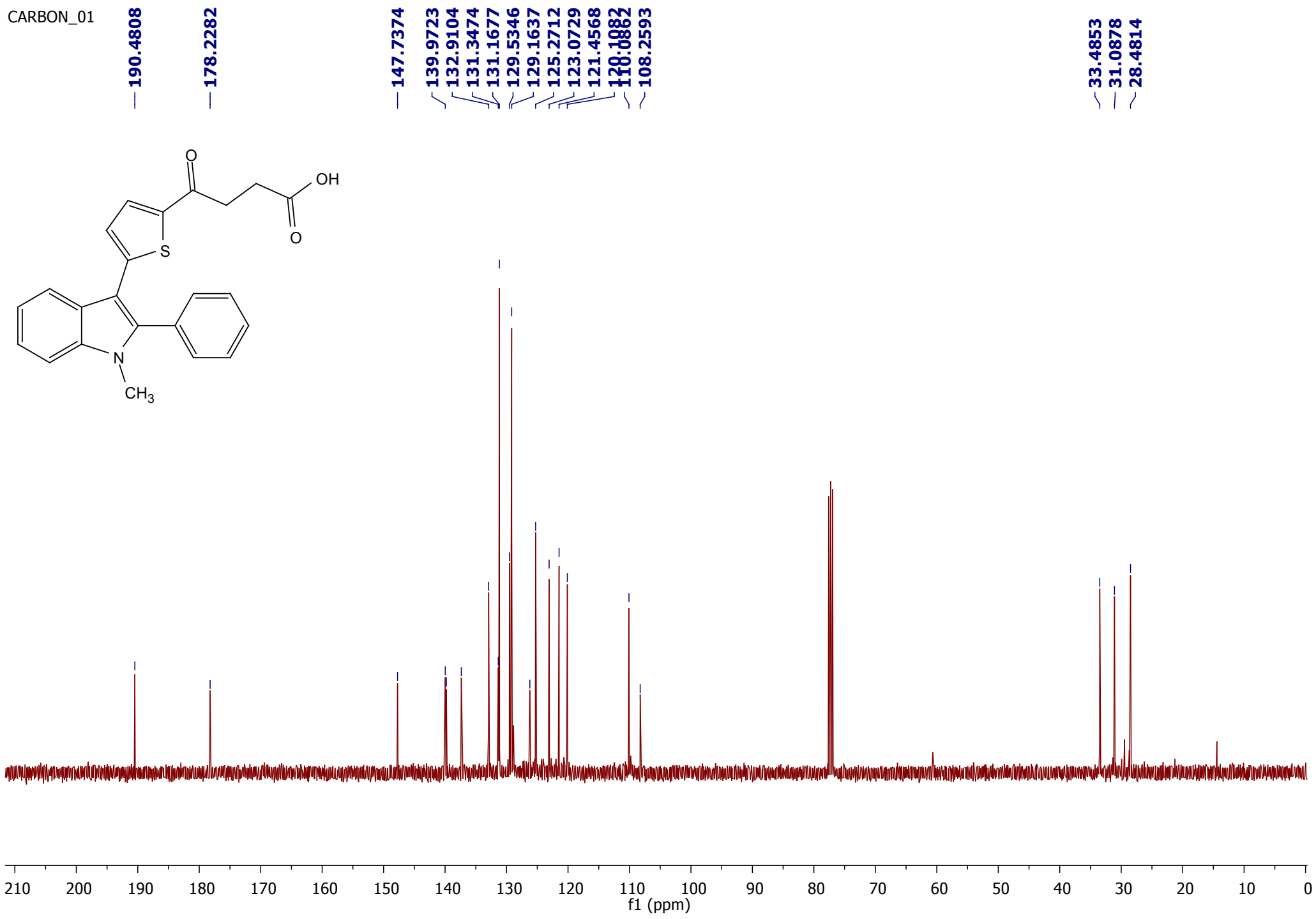

Figure S15. ^{13}C NMR spectrum of 4a.

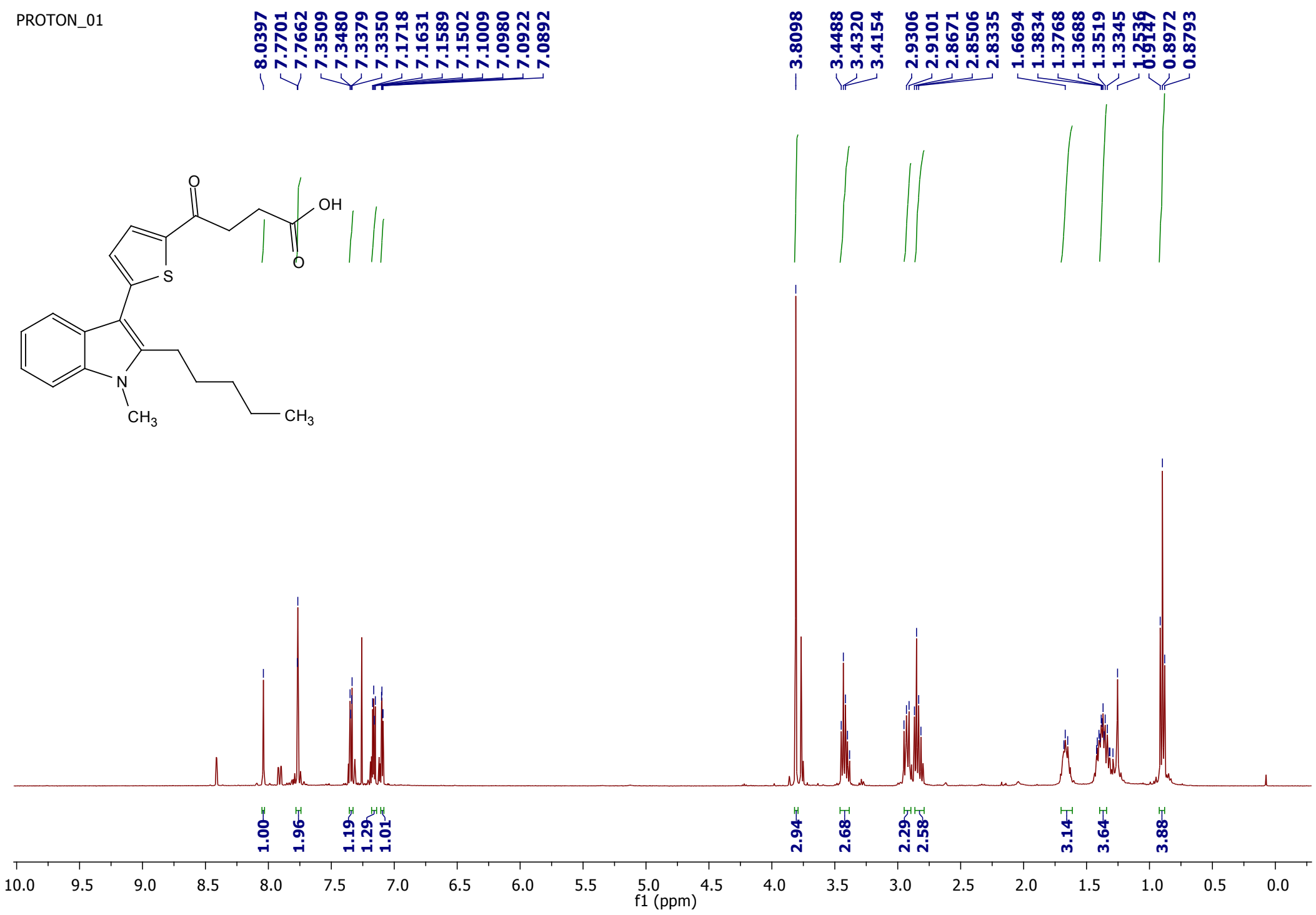

Figure S16. ^{1}H NMR spectrum of 4b.

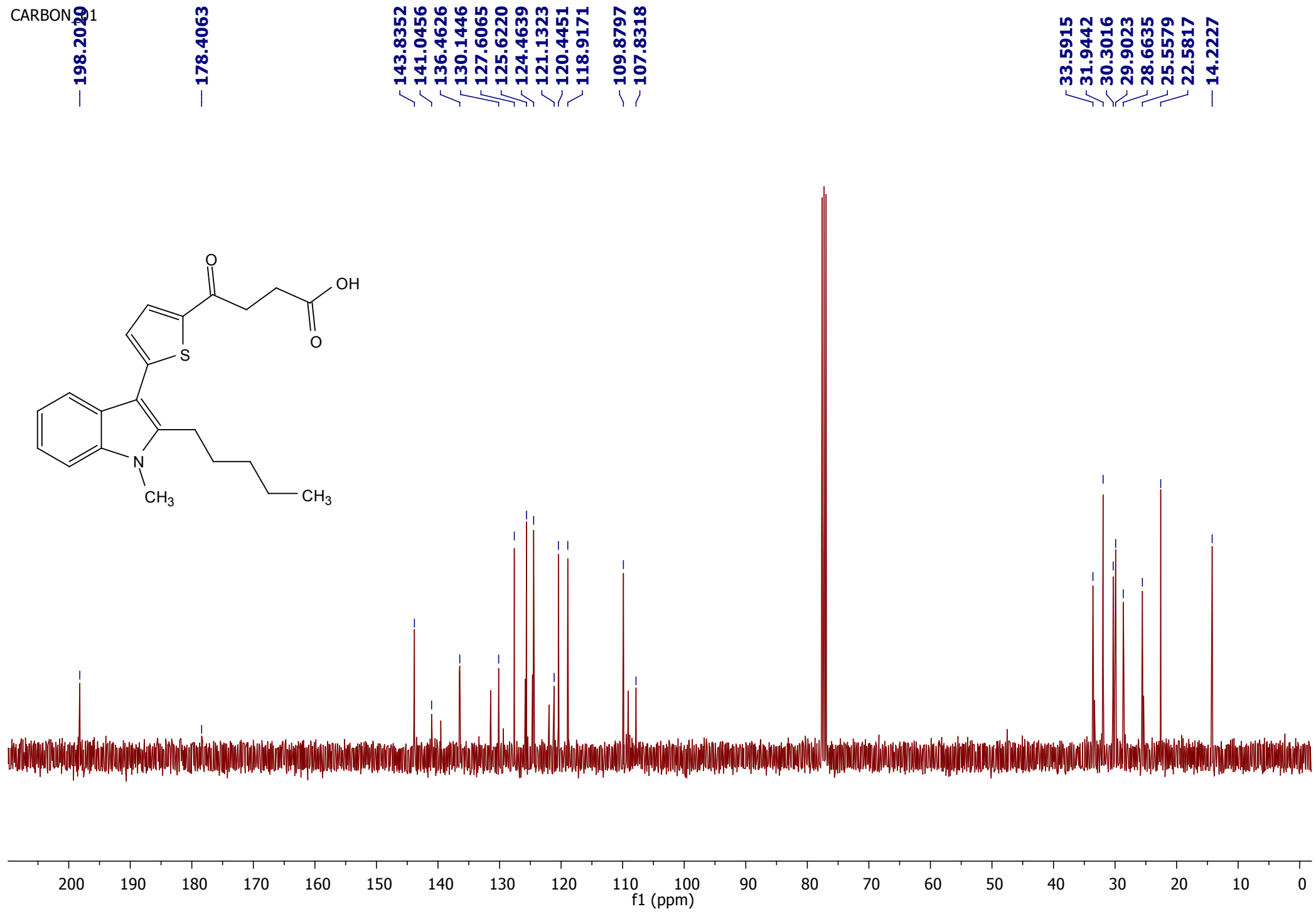

Figure S17. ^{13}C NMR spectrum of 4b.

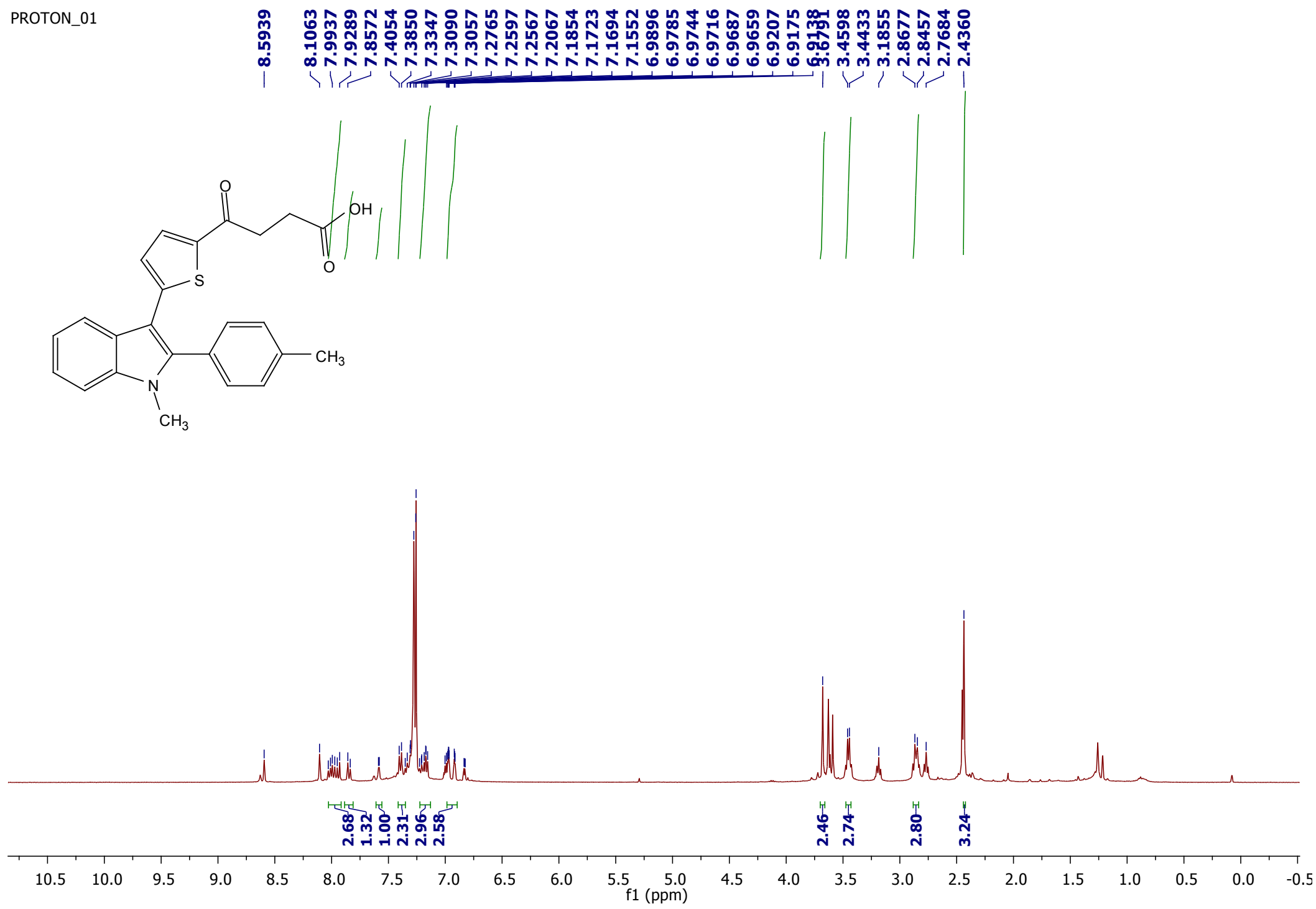

Figure S18. ^{1}H NMR spectrum of 4c.

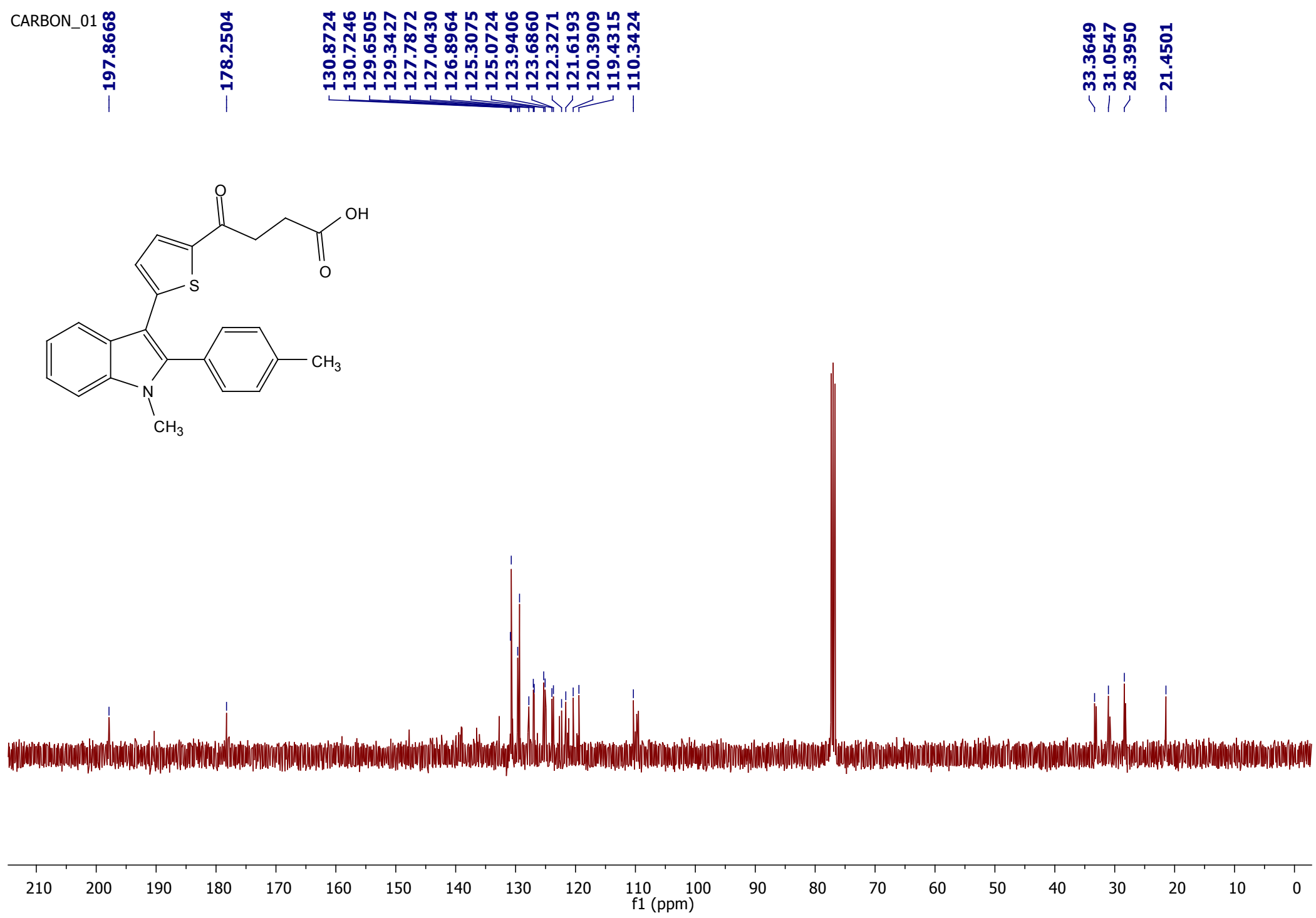

Figure *S19*. ^{13}C NMR spectrum of 4c.

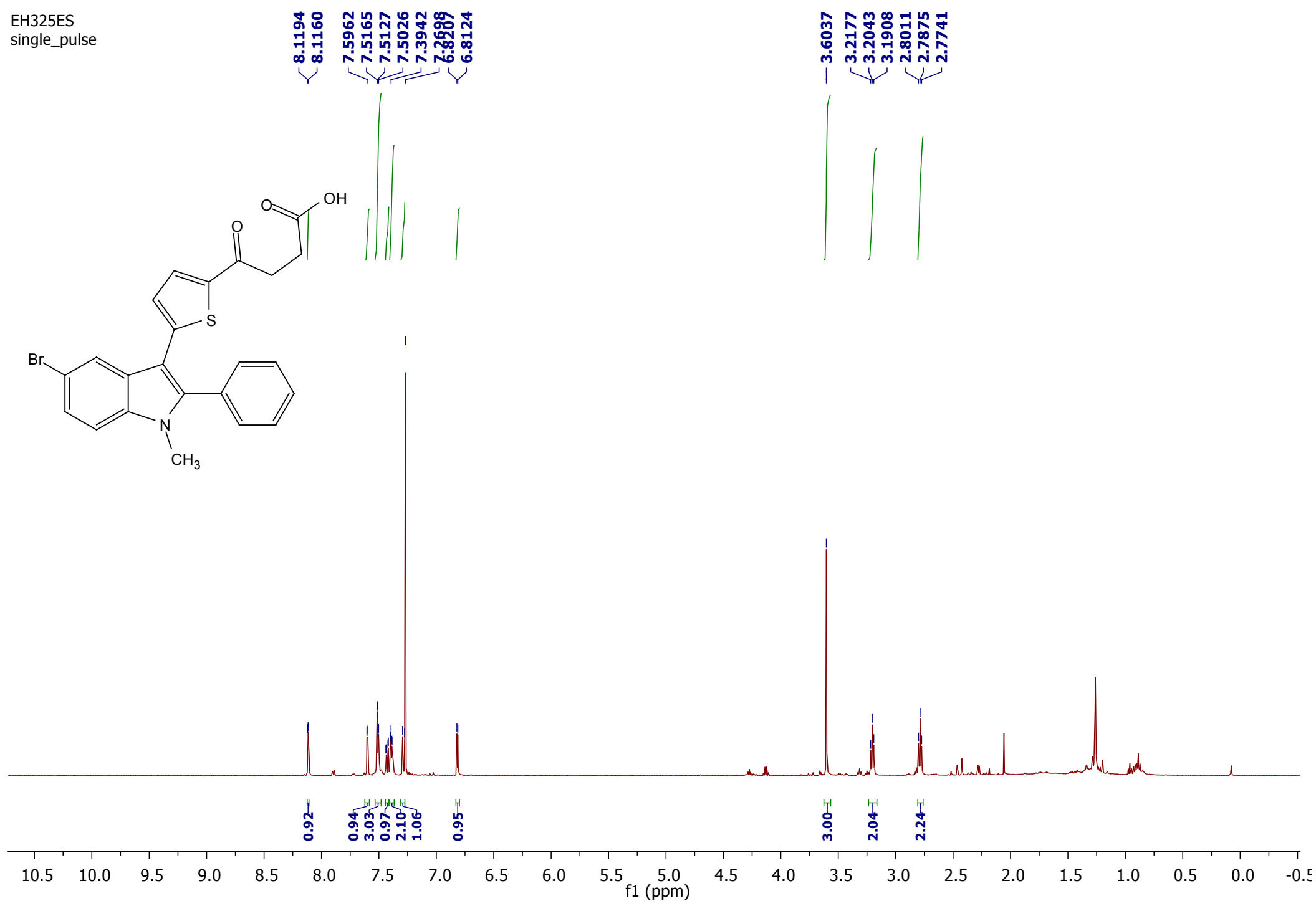

Figure S20. ^{1}H NMR spectrum of 4d.

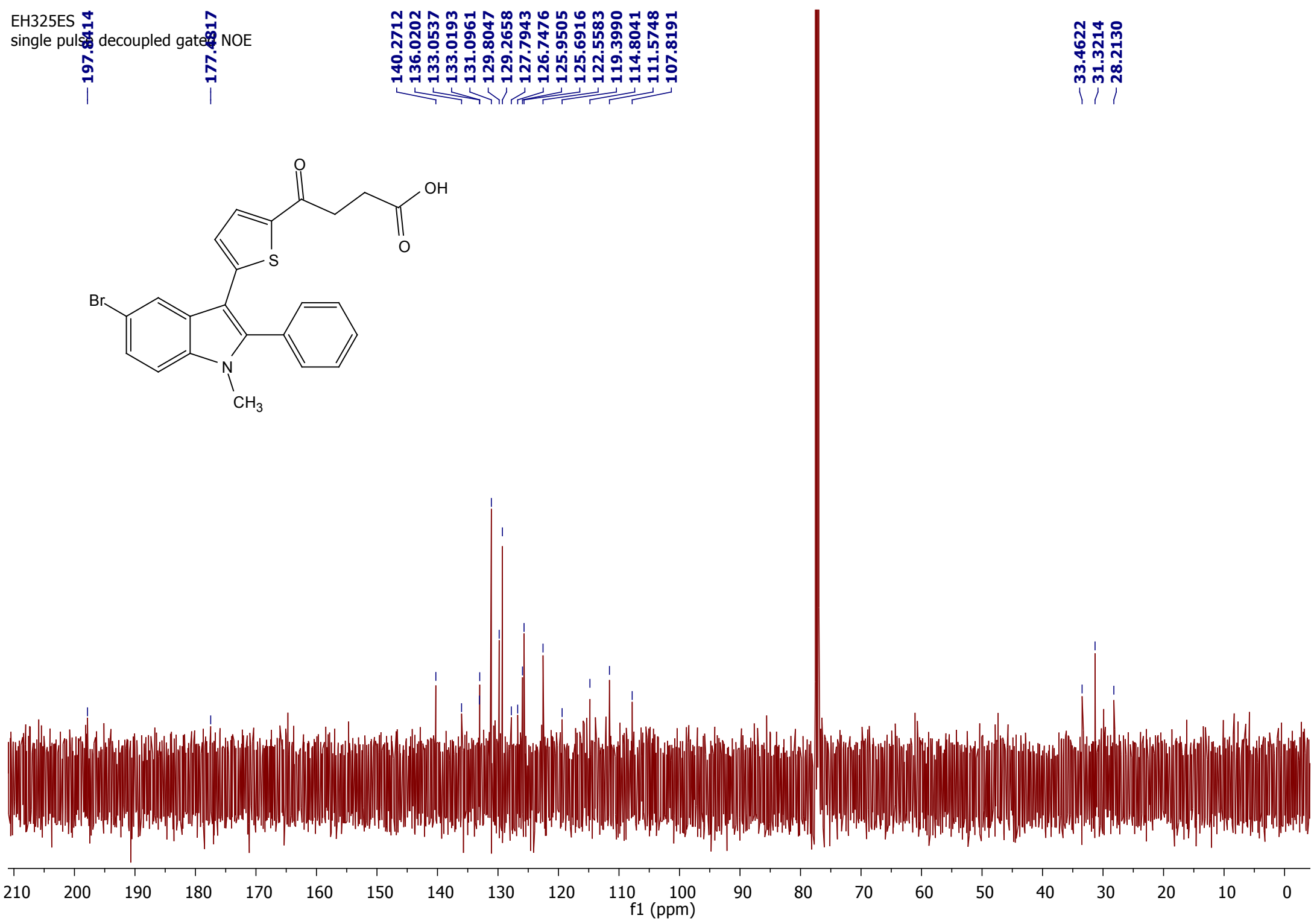

Figure S21. ^{13}C NMR spectrum of 4d.

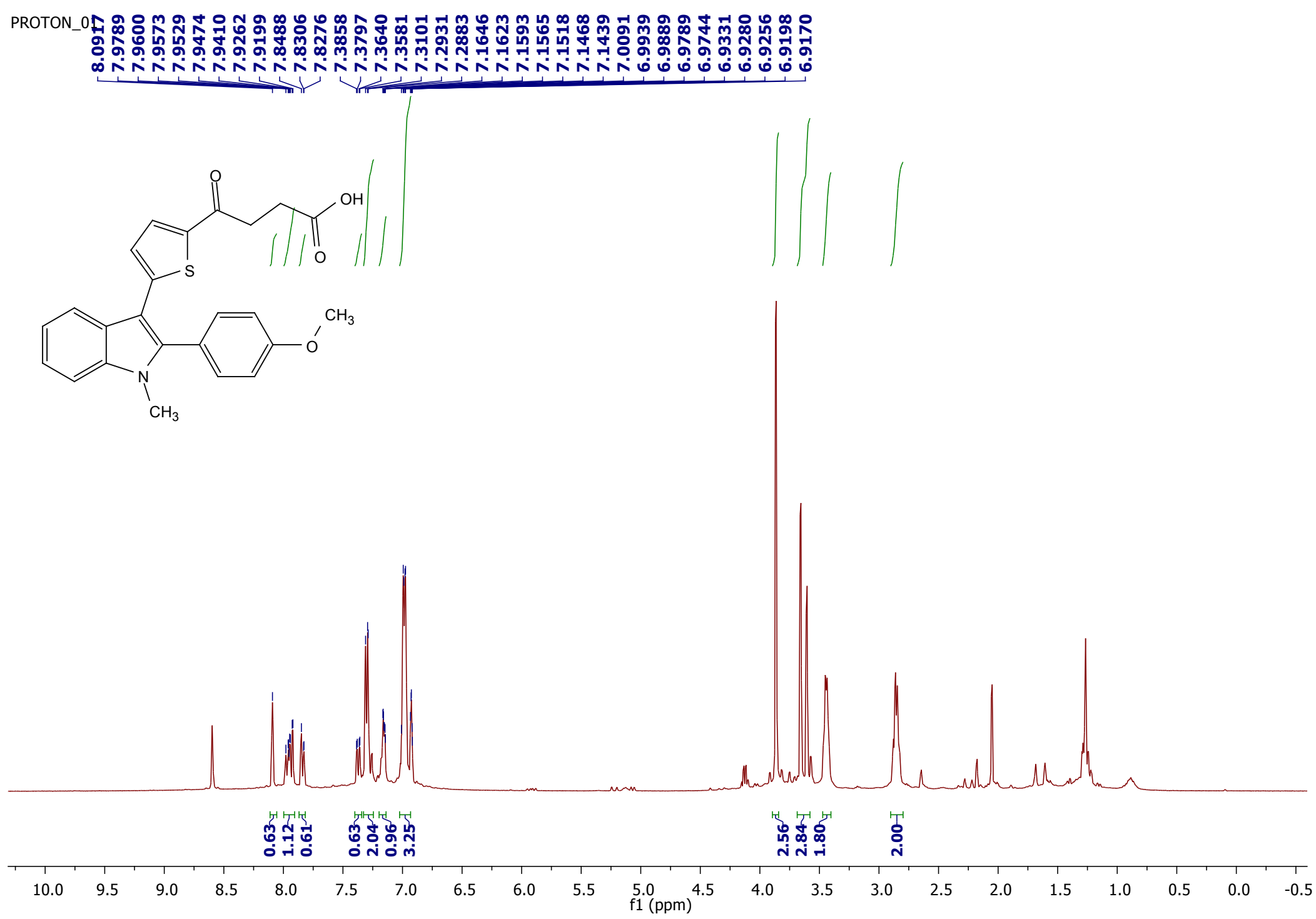

Figure S22. ^{1}H NMR spectrum of 4e.

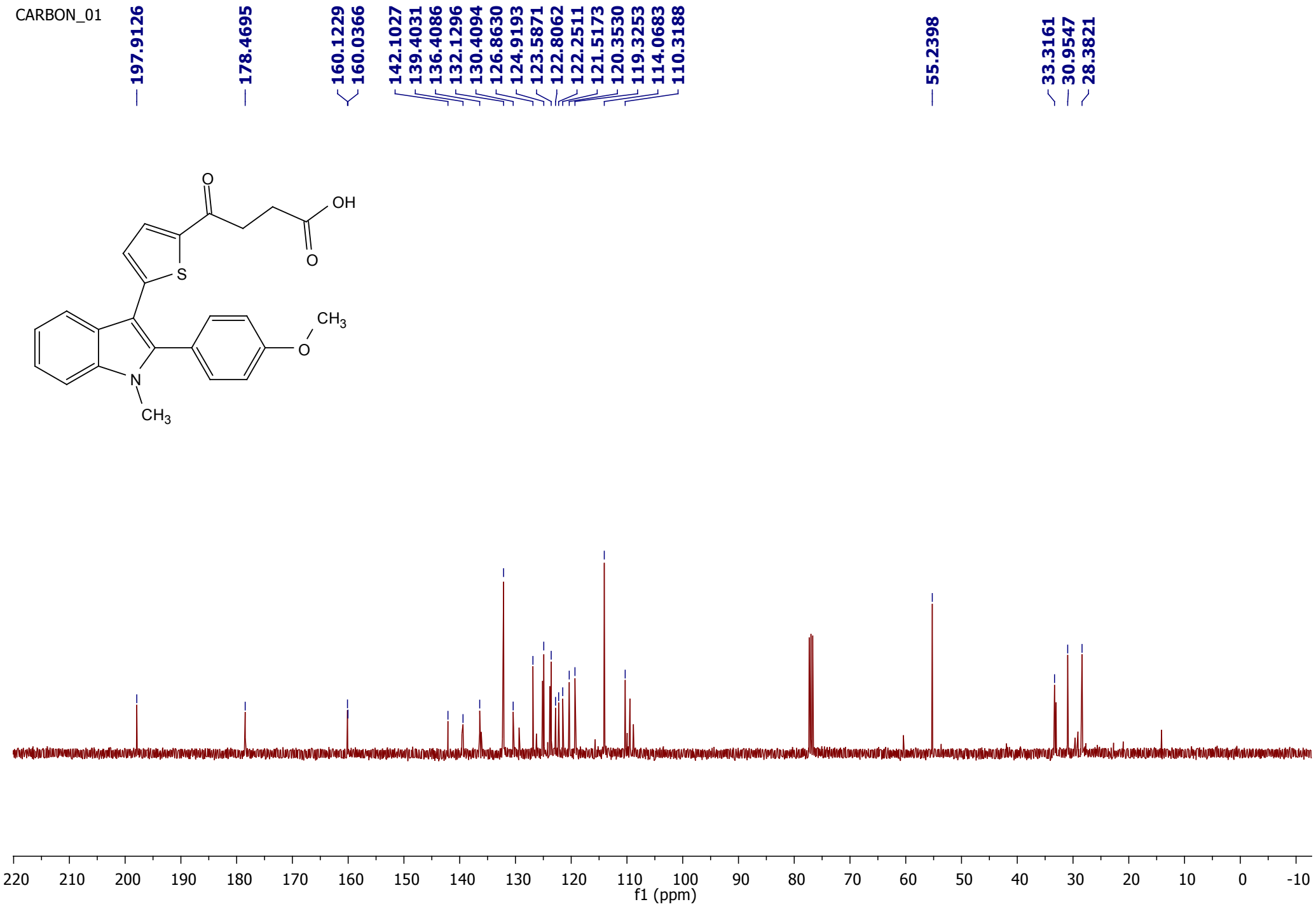

Figure S23. ^{13}C NMR spectrum of 4e.

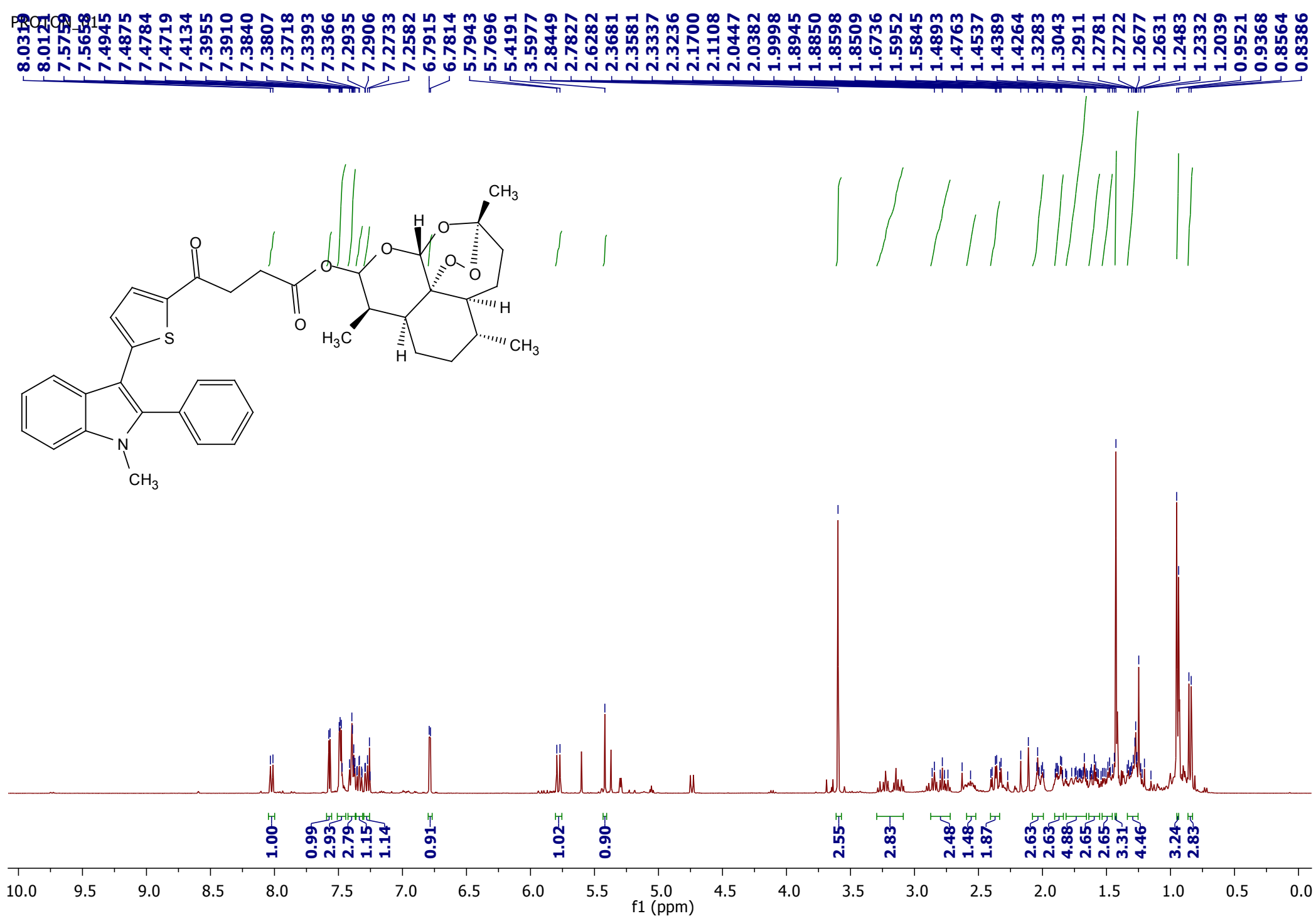

Figure S24. ^{1}H NMR spectrum of D1.

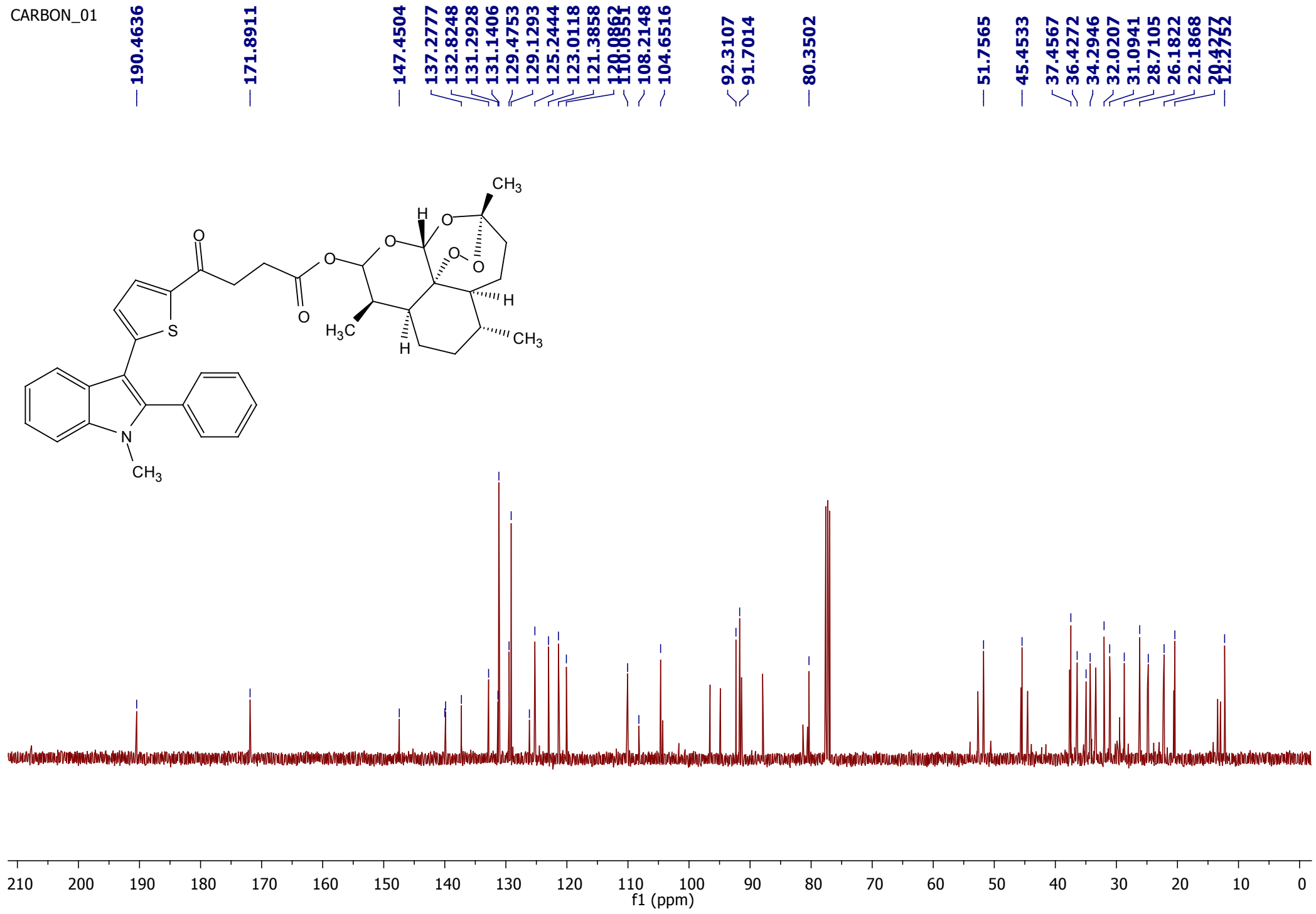

Figure S25. ^{13}C NMR spectrum of D1.

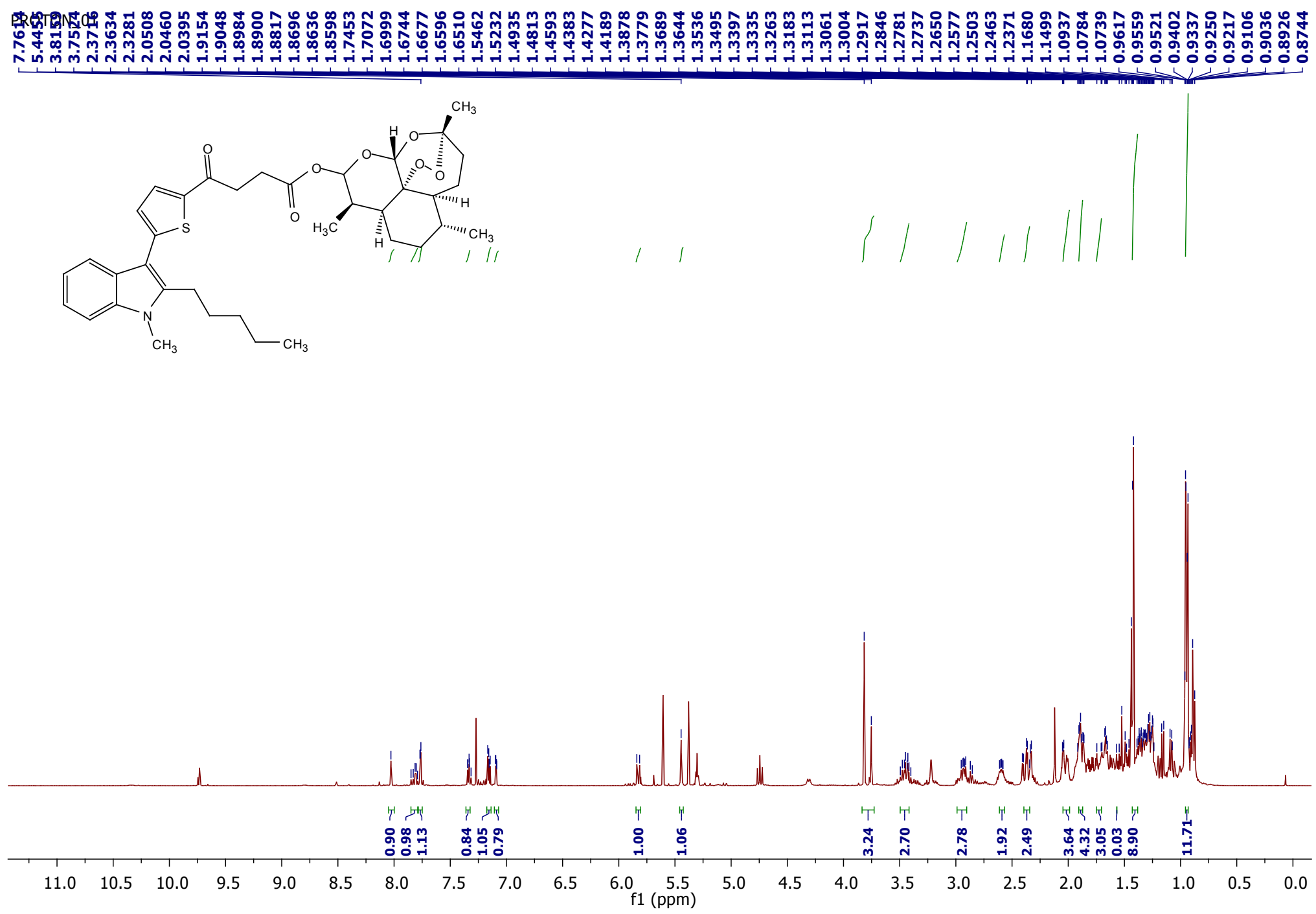

Figure S26. ^{1}H NMR spectrum of D2.

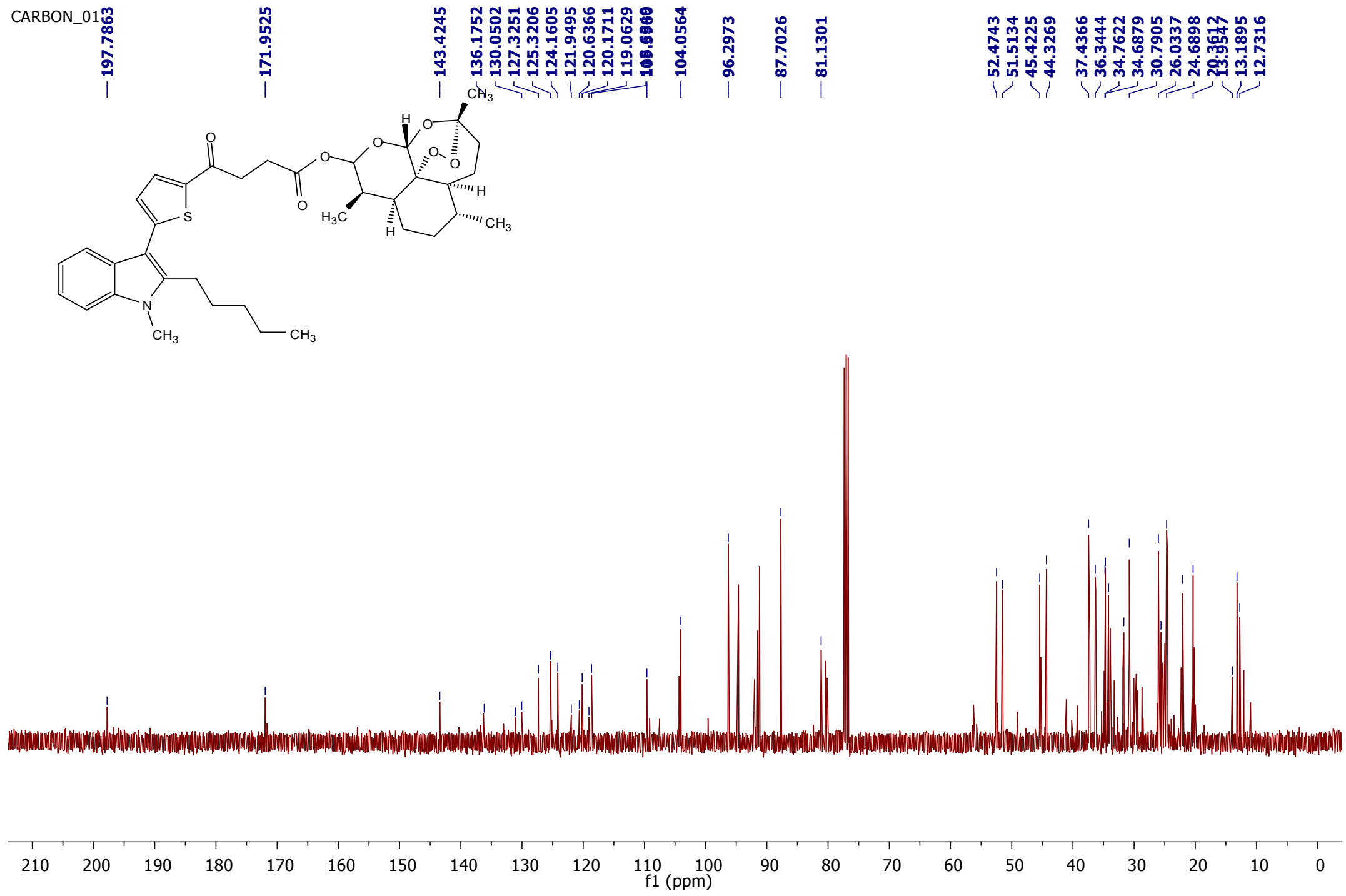

Figure S27. ^{13}C NMR spectrum of D2.

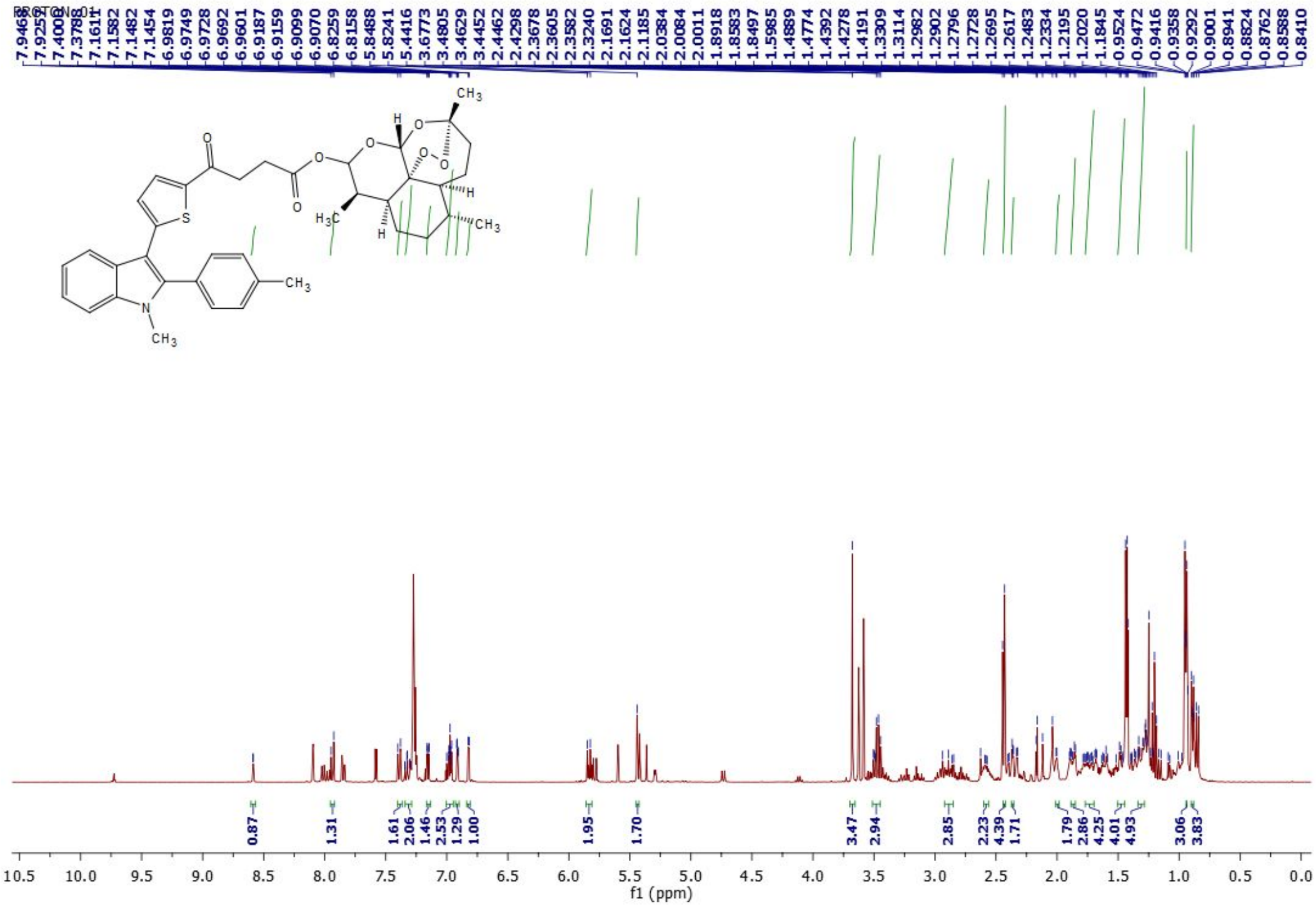

Figure S28. ^{1}H NMR spectrum of D3.

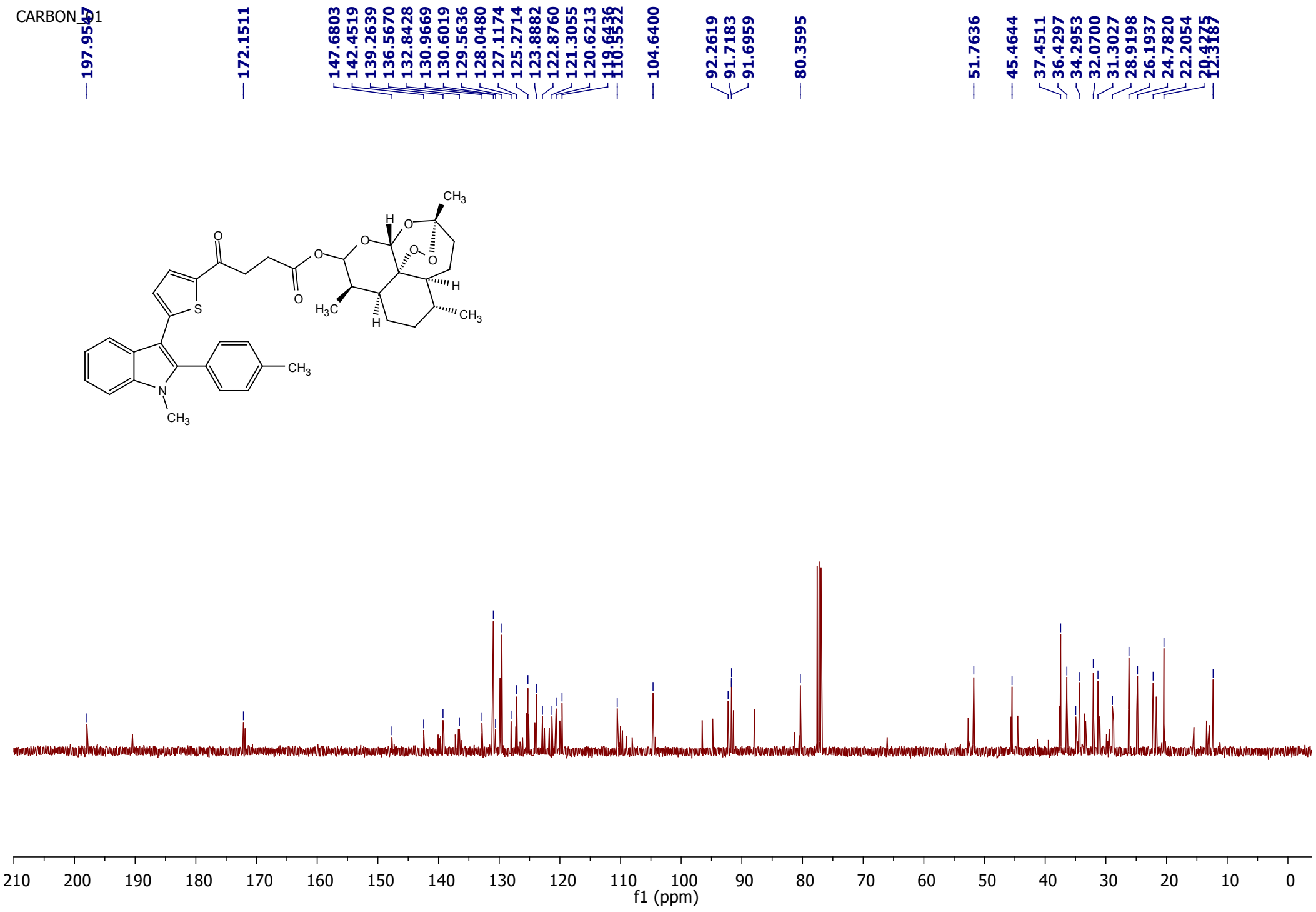

Figure S29. ^{13}C NMR spectrum of D3.

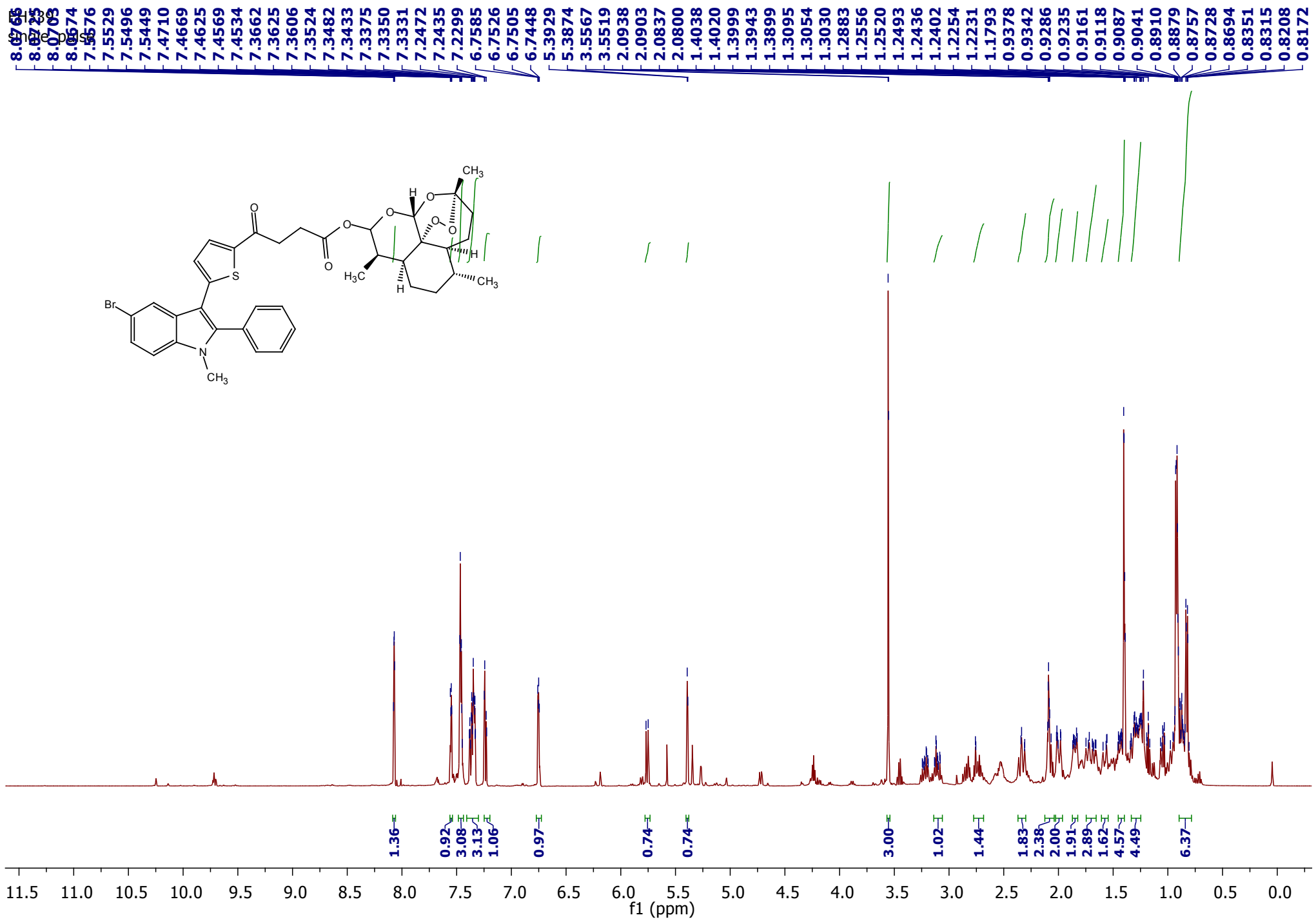

Figure S30. ^{1}H NMR spectrum of D4.

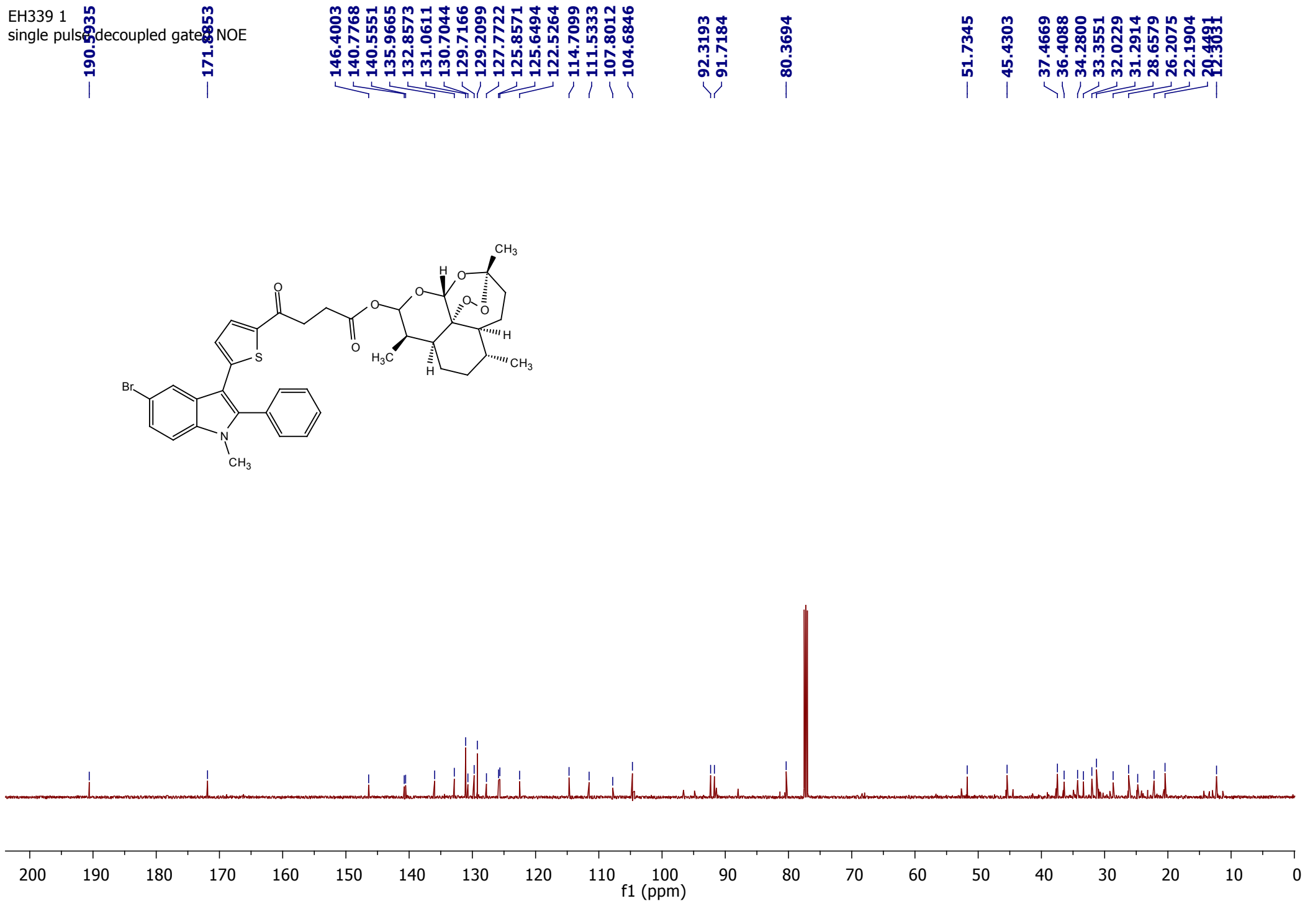

Figure S31. ^{13}C NMR spectrum of D4.

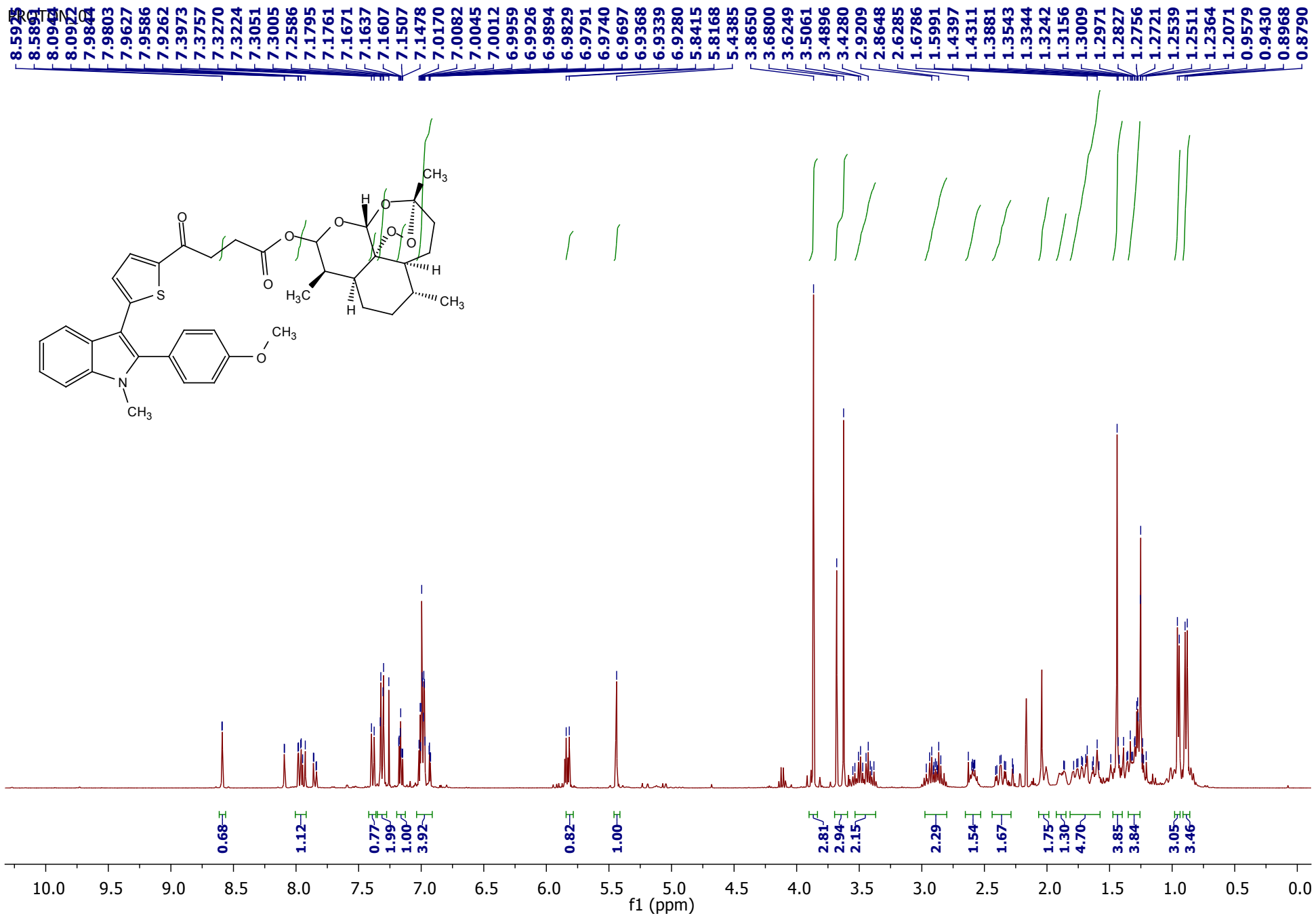

Figure S32. ^{1}H NMR spectrum of D5.

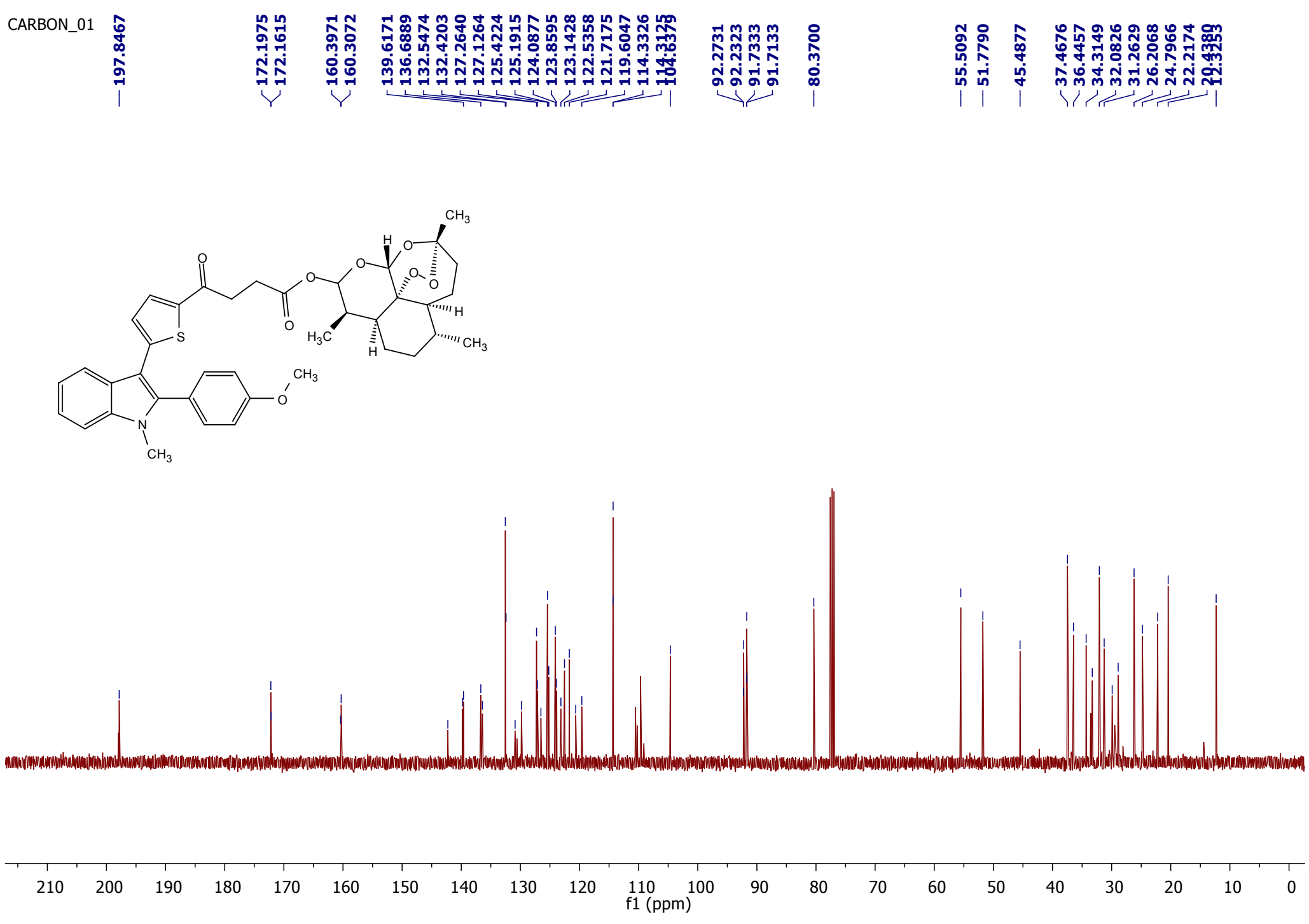

Figure S33. ^{13}C NMR spectrum of D5.

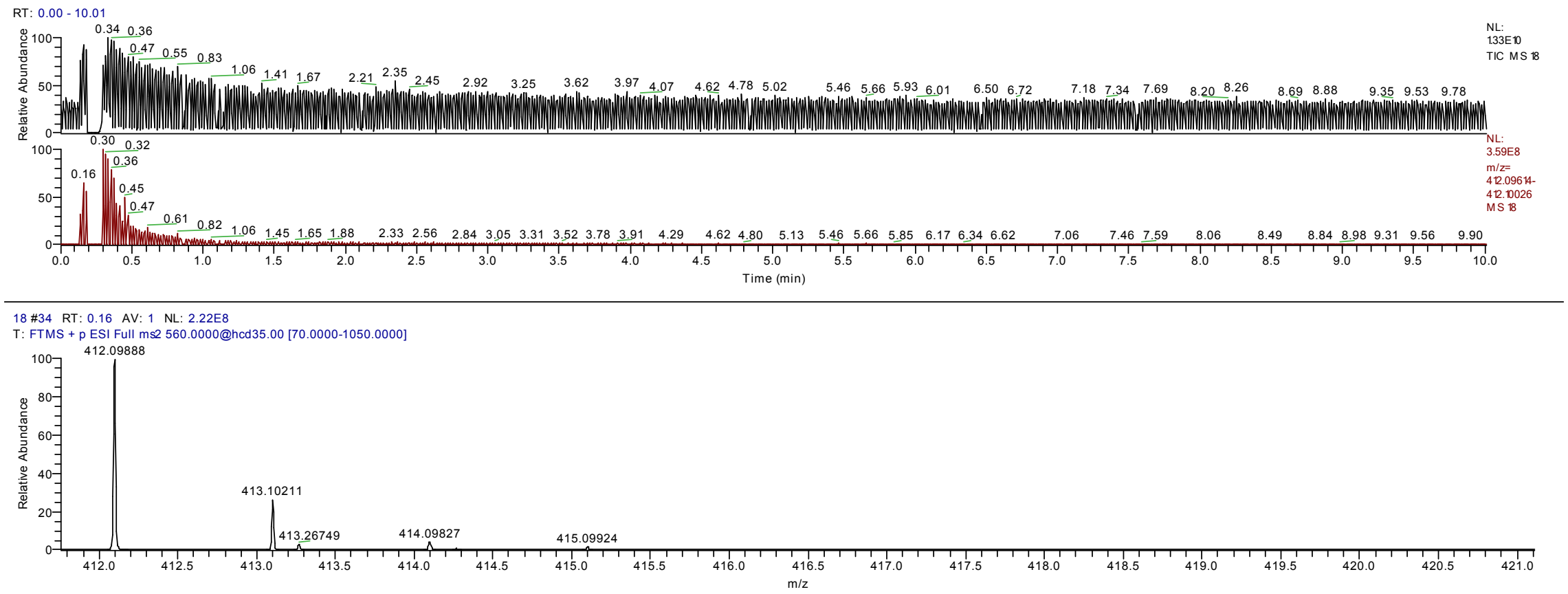

Figure S34. LC MS/MS Spectrum of 4a.

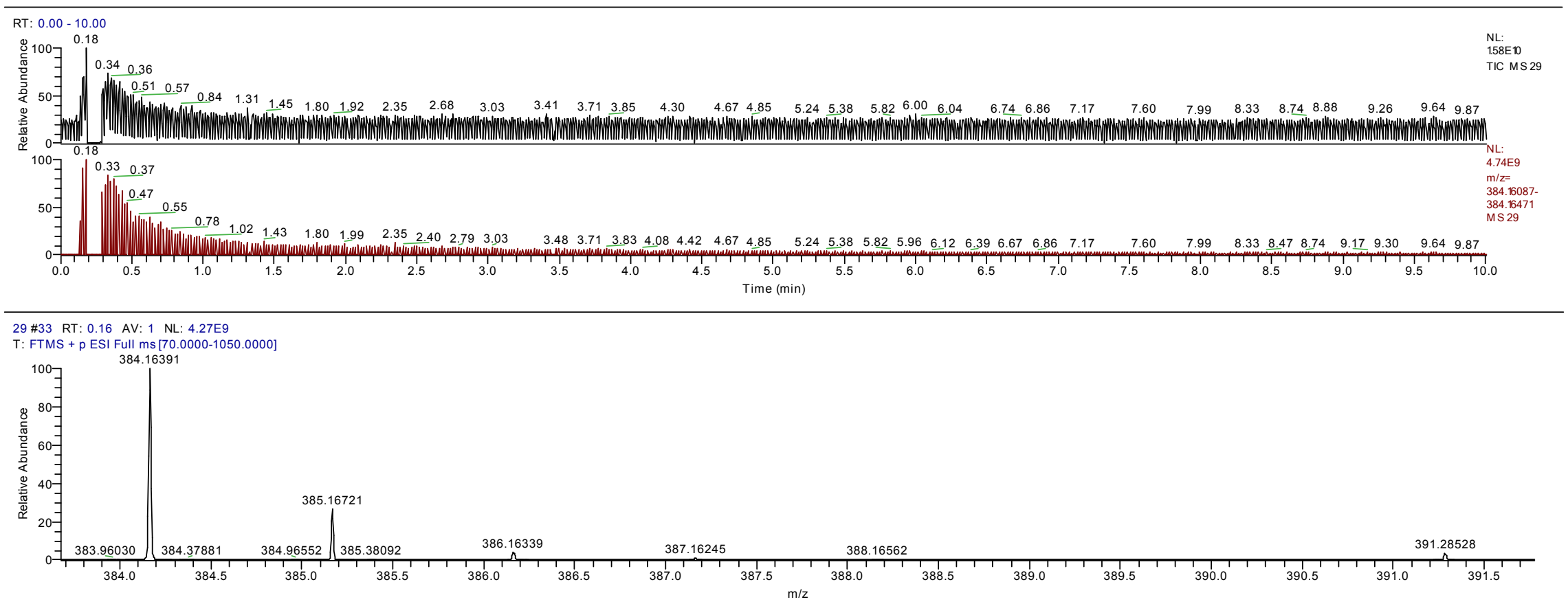

Figure S35. LC MS/MS Spectrum of 4b.

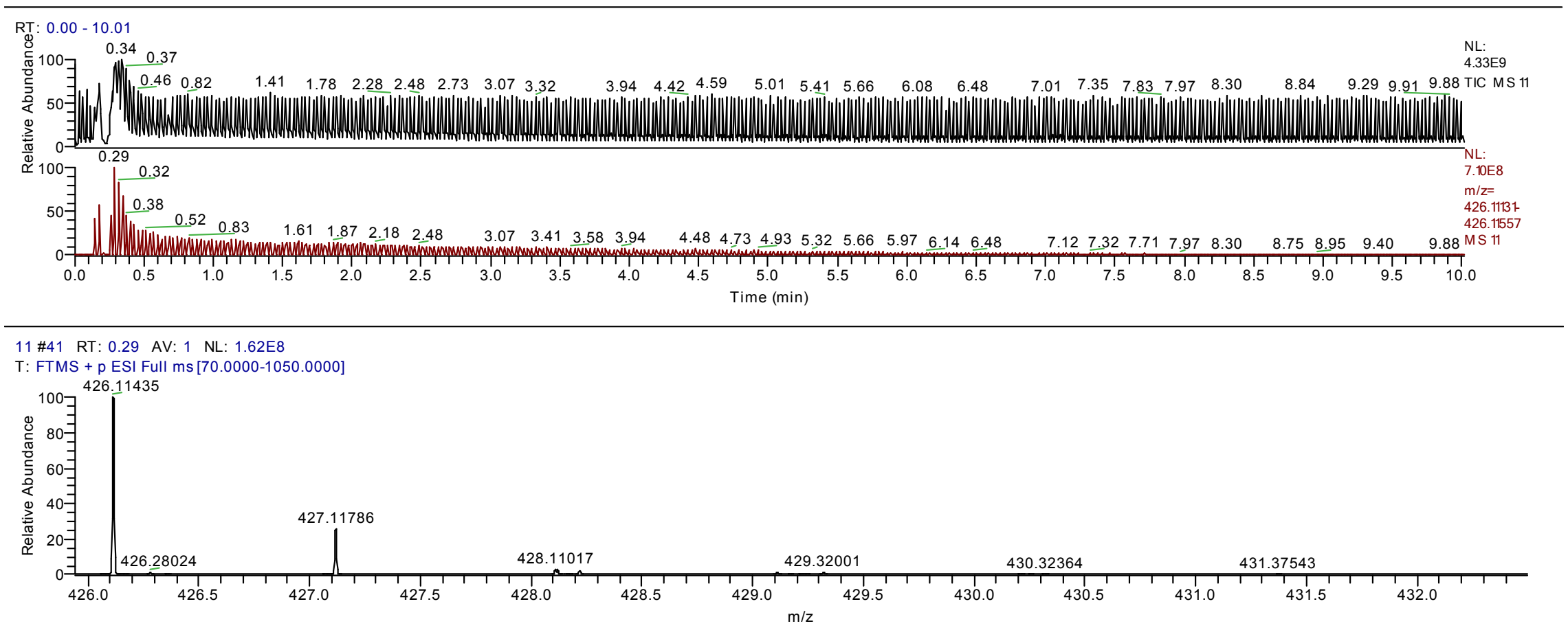

Figure S36. LC MS/MS Spectrum of 4c.

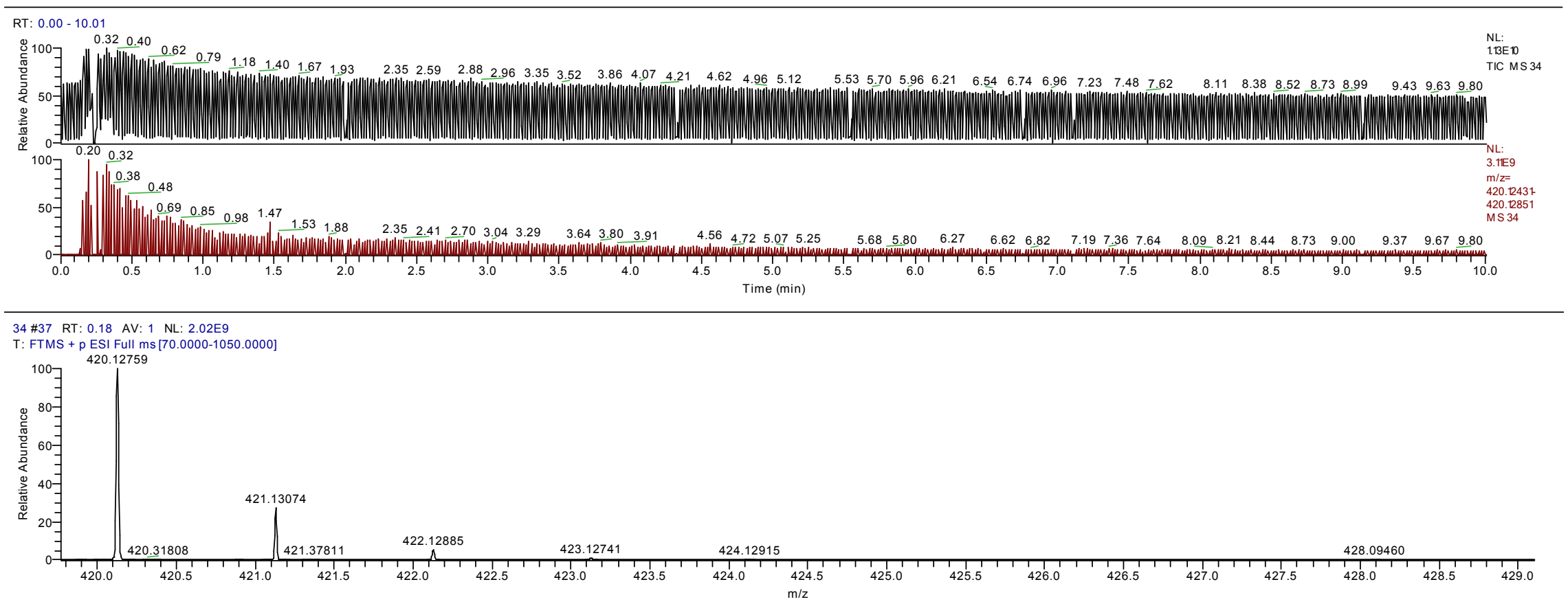

Figure S37. LC MS/MS Spectrum of 4e.

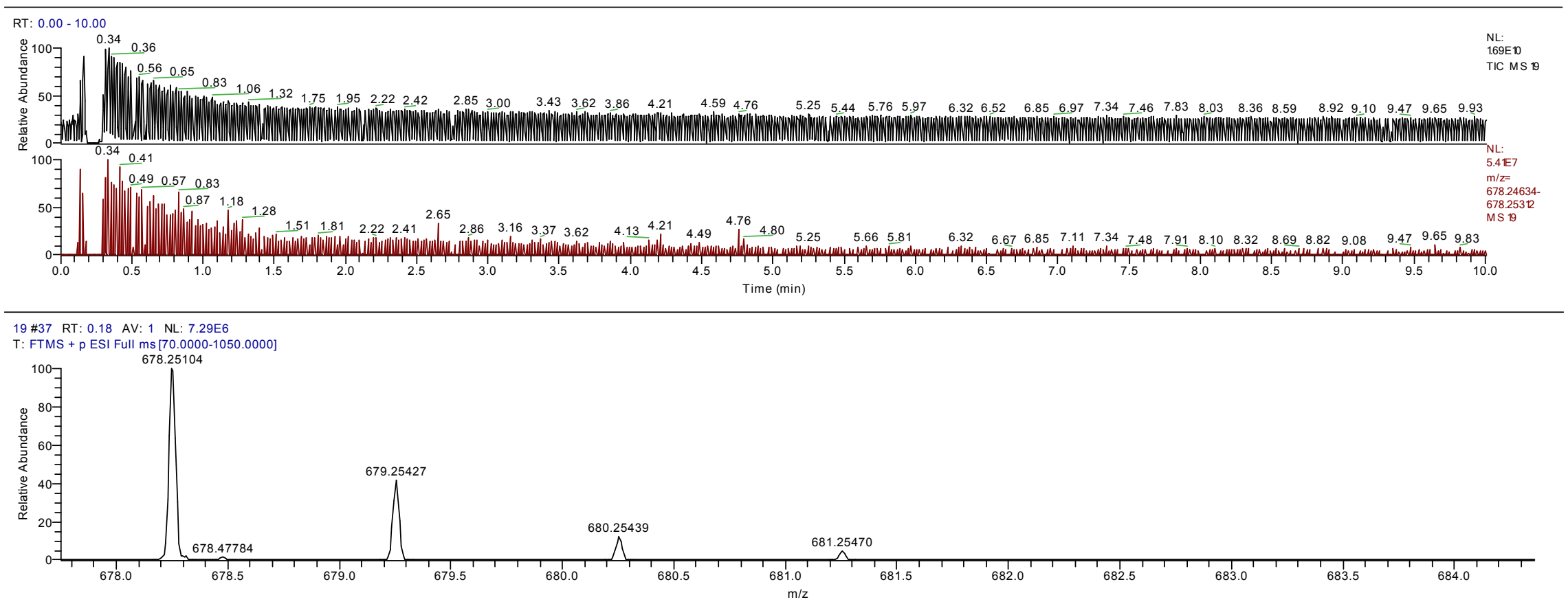

Figure S38. LC MS/MS Spectrum of D1.

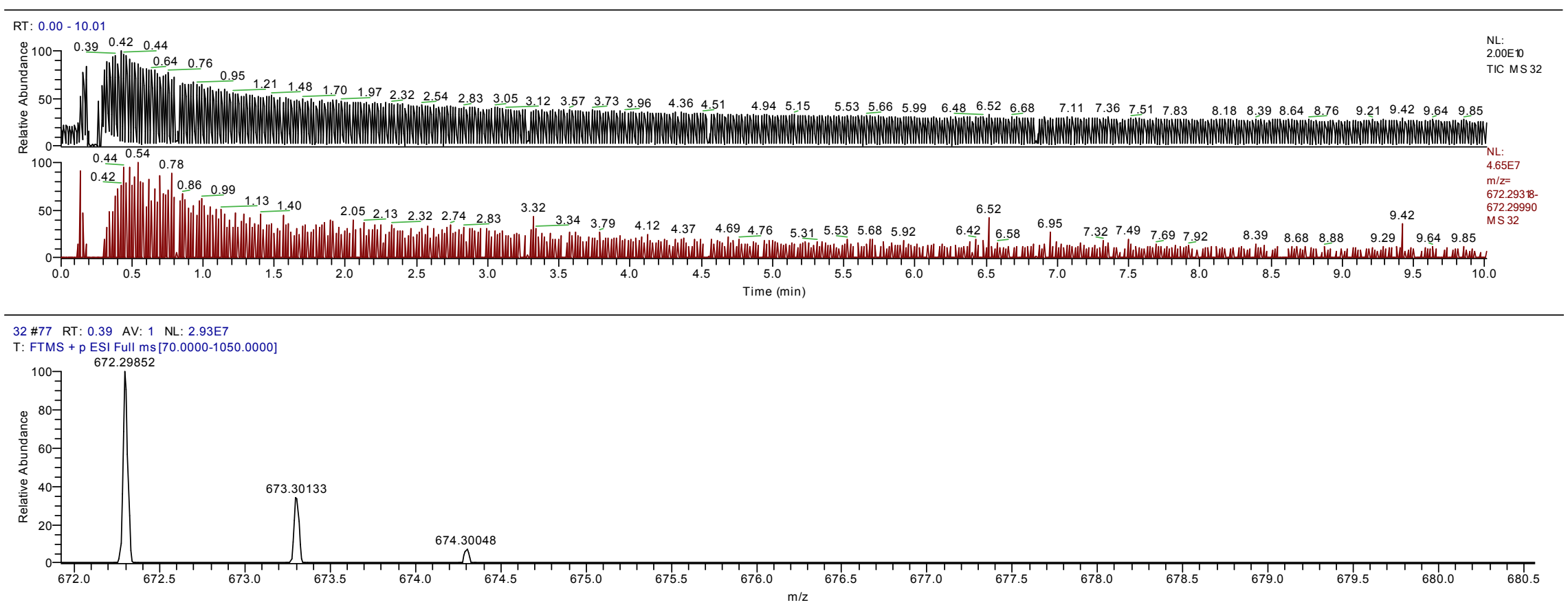

Figure S39. LC MS/MS Spectrum of D2.

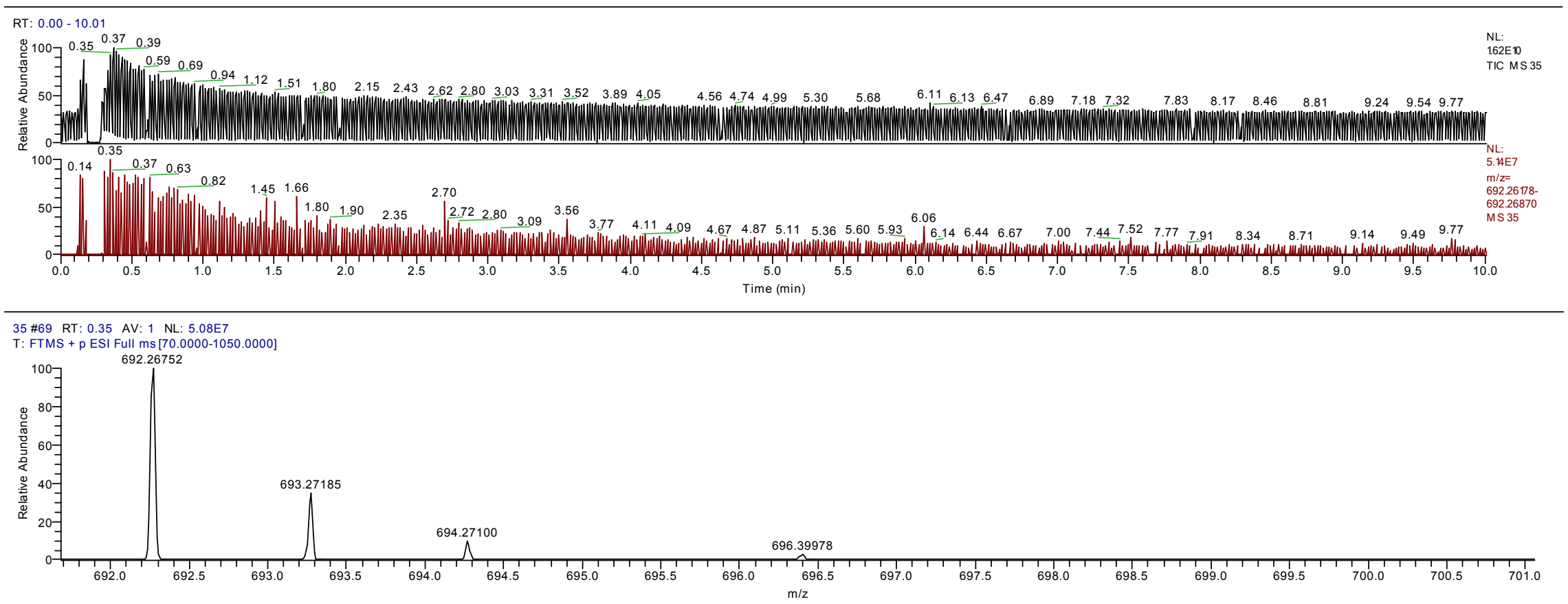

Figure S40. LC MS/MS Spectrum of D3.

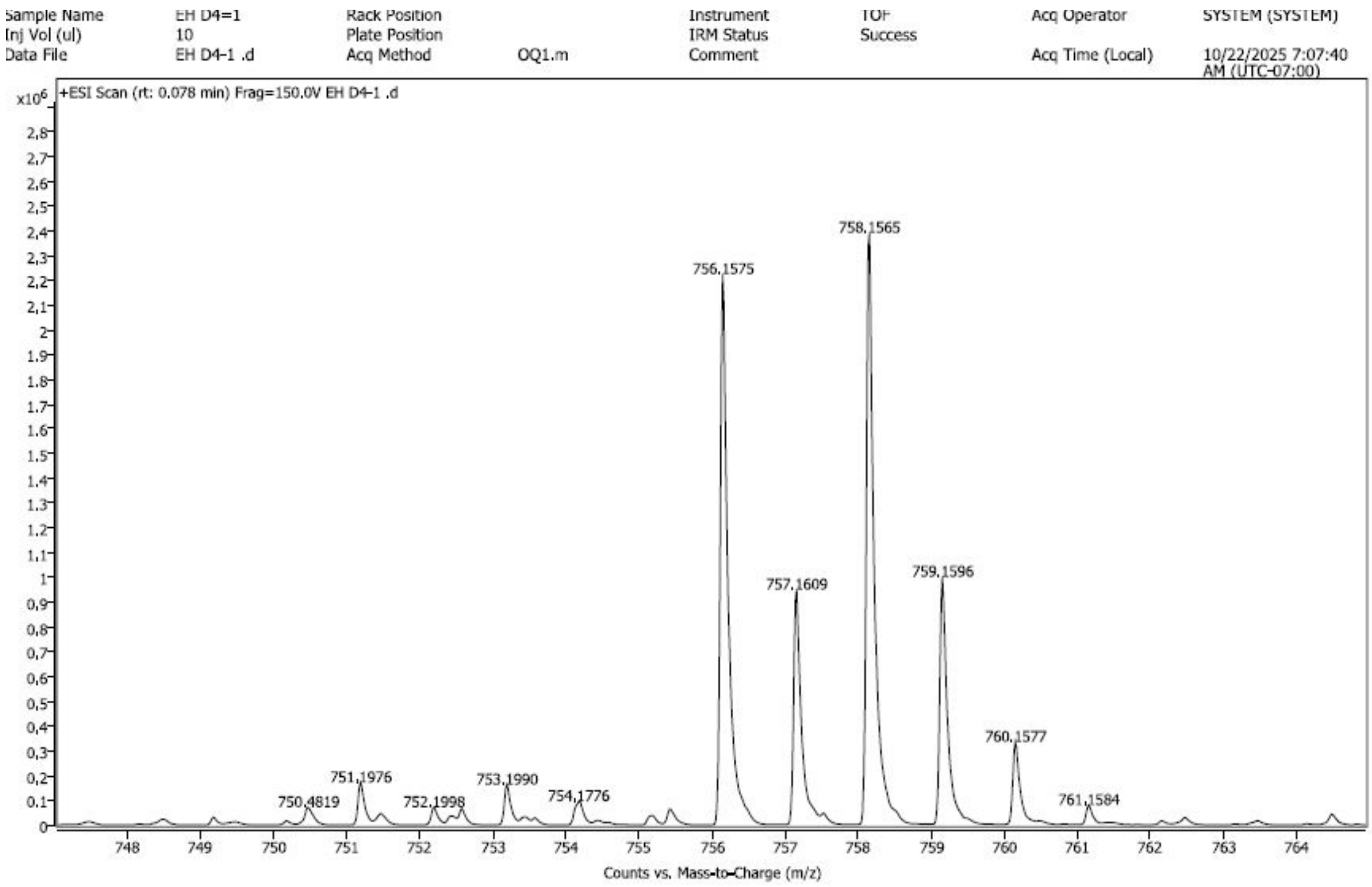

Figure S41. LC MS/MS Spectrum of D4.

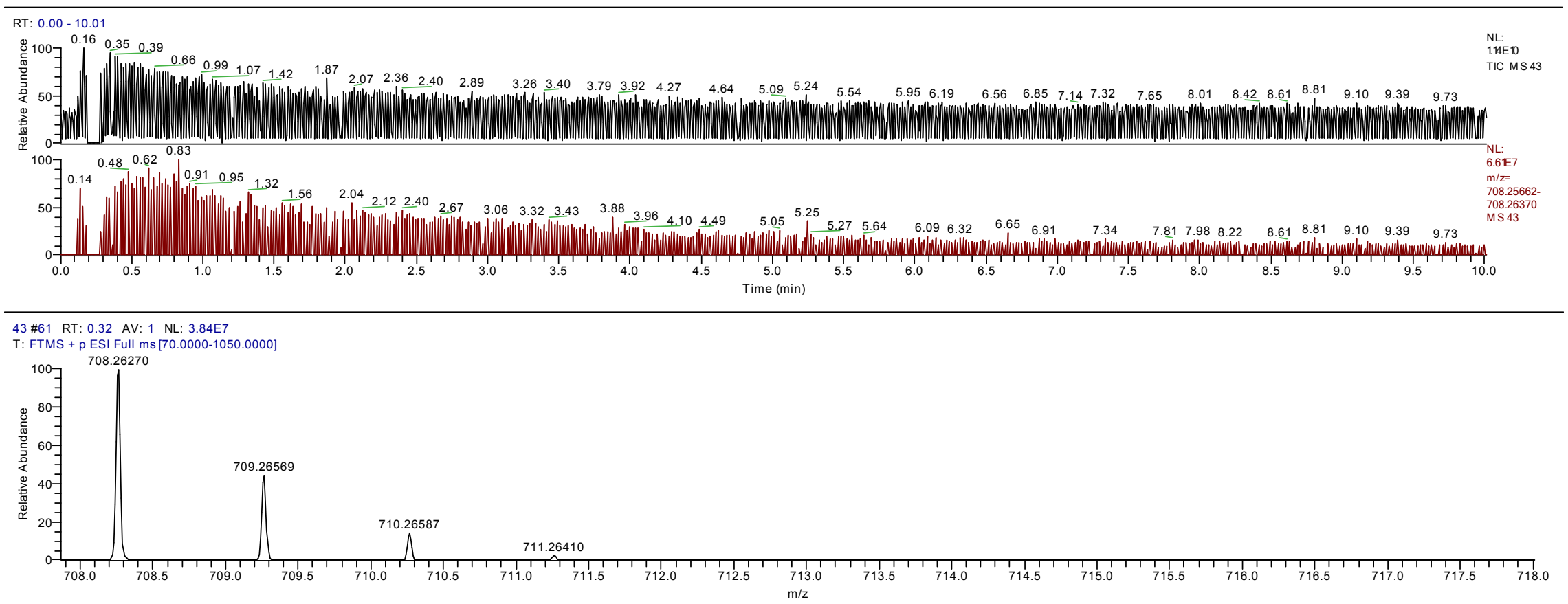

Figure S42. LC MS/MS Spectrum of D5.

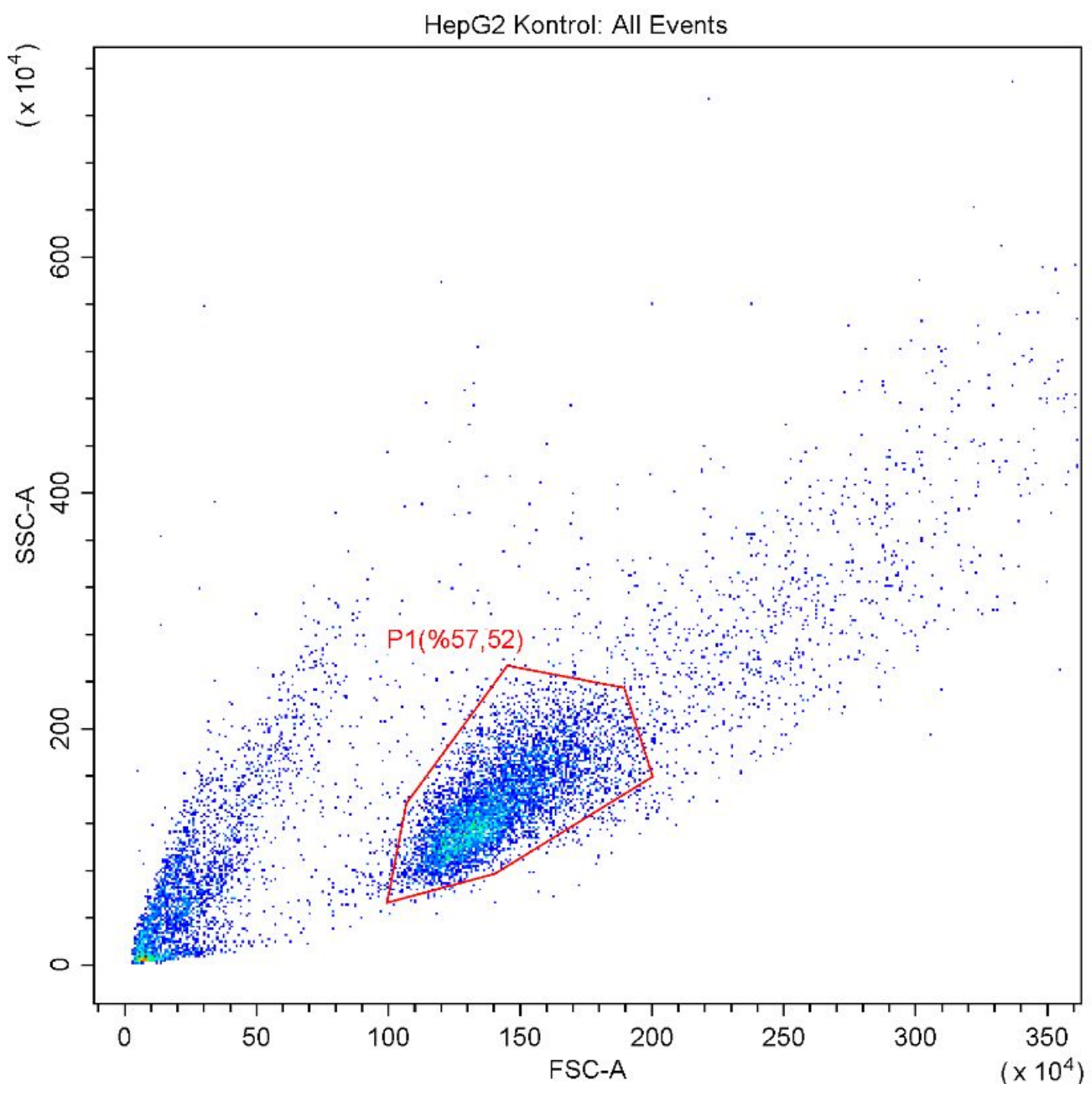

Figure S43. The main cell population was identified using an FSC-A versus SSC-A plot and selected within the P1 gate.

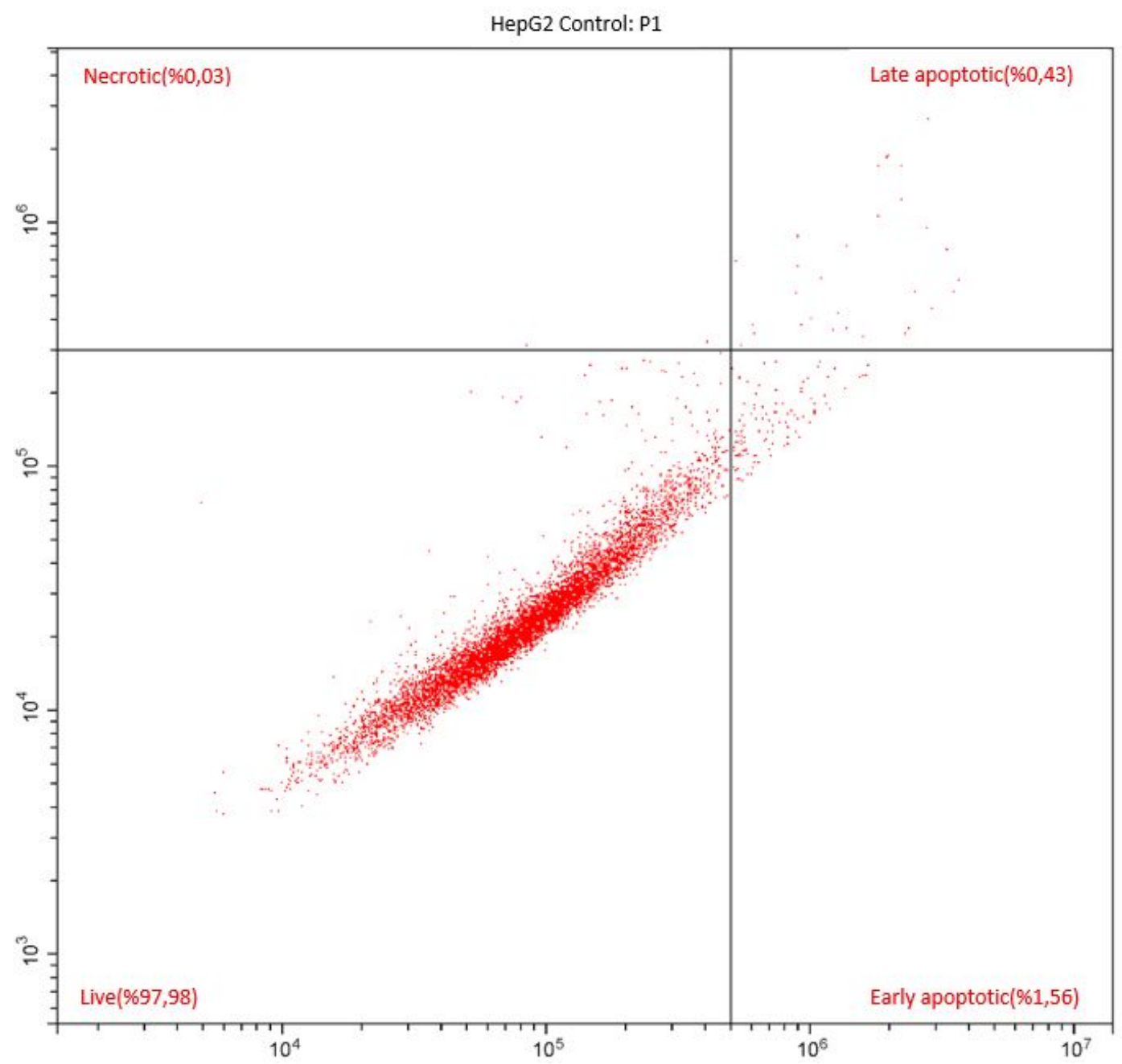

Figure S44. Events within P1 were analyzed using FITC-A versus PE-A fluorescence. The lower-left quadrant represents viable cells (Annexin V−/PI−), the lower-right quadrant represents early apoptotic cells (Annexin V+/PI−), the upper-right quadrant represents late apoptotic/secondary necrotic cells (Annexin V+/PI+), and the upper-left quadrant represents necrotic cells (Annexin V−/PI+). Total apoptosis was calculated as the sum of early and late apoptotic populations.

Table S1. qPCR results for HepG2 cell line

HEPG2(D1)	Ct		ORT		Ct		SD	ORT	Delta Ct	DDCt	2fold (2^-deltadelta c	% EXP
GAPDH - CONTROL	16,92	16,95	16,94	BAX - CONTROL	25,42	25,42	0,00	25,42	8,49			100,00
GAPDH - D1	17,85	17,88	17,87	BAX - D1	25,95	25,83	0,08	25,89	8,03	-0,46	1,38	137,55
GAPDH - CONTROL	16,92	16,95	16,94	P53 - CONTROL	24,76	24,47	0,21	24,62	7,68			100,00
GAPDH - D1	17,85	17,88	17,87	P53 - D1	24,02	24,33	0,22	24,18	6,31	-1,37	2,58	258,47
GAPDH - CONTROL	16,92	16,95	16,94	BCL 2 - CONTROL	25,59	26	0,29	25,80	8,86			100,00
GAPDH - D1	17,85	17,88	17,87	BCL 2 - D1	27,3	27,25	0,04	27,28	9,41	0,55	0,68	68,30
GAPDH - CONTROL	16,92	16,95	16,94	CASP3 - CONTROL	21,68	21,94	0,18	21,81	4,88			100,00
GAPDH - D1	17,85	17,88	17,87	CASP3 - D1	21,01	21,05	0,03	21,03	3,17	-1,71	3,27	327,16
GAPDH - CONTROL	16,92	16,95	16,94	CASP8 - CONTROL	26,19	26,39	0,14	26,29	9,36			100,00
GAPDH - D1	17,85	17,88	17,87	CASP8 - D1	25,59	26,02	0,30	25,81	7,94	-1,42	2,67	266,66
GAPDH - CONTROL	16,92	16,95	16,94	CASP9 - CONTROL	24,89	24,94	0,04	24,92	7,98			100,00
GAPDH - D1	17,85	17,88	17,87	CASP9 - D1	25,03	24,97	0,04	25,00	7,14	-0,84	1,80	179,63

HEPG2(D2)	Ct		ORT		Ct		SD	ORT	Delta Ct	DDCt	2fold (2^-deltadelta c	% EXP
GAPDH - CONTROL	16,92	16,95	16,94	BAX - CONTROL	25,42	25,42	0,00	25,42	8,49			100,00
GAPDH - D2	17,93	17,71	17,82	BAX - D2	25,09	24,89	0,14	24,99	7,17	-1,32	2,49	248,80
GAPDH - CONTROL	16,92	16,95	16,94	P53 - CONTROL	24,76	24,47	0,21	24,62	7,68			100,00
GAPDH - D2	17,93	17,71	17,82	P53 - D2	24,15	24,25	0,07	24,20	6,38	-1,30	2,46	246,23
GAPDH - CONTROL	16,92	16,95	16,94	BCL 2 - CONTROL	26,61	26,93	0,23	26,77	9,84			100,00
GAPDH - D2	17,93	17,71	17,82	BCL 2 - D2	27,75	27,93	0,13	27,84	10,02	0,19	0,88	87,96
GAPDH - CONTROL	16,92	16,95	16,94	CASP3 - CONTROL	21,68	21,94	0,18	21,81	4,88			100,00
GAPDH - D2	17,93	17,71	17,82	CASP3 - D2	21,03	21,01	0,01	21,02	3,20	-1,68	3,19	319,32
GAPDH - CONTROL	16,92	16,95	16,94	CASP8 - CONTROL	26,19	26,39	0,14	26,29	9,36			100,00
GAPDH - D2	17,93	17,71	17,82	CASP8 - D2	26,44	26,93	0,35	26,69	8,87	-0,49	1,40	140,44
GAPDH - CONTROL	16,92	16,95	16,94	CASP9 - CONTROL	24,89	24,94	0,04	24,92	7,98			100,00
GAPDH - D2	17,93	17,71	17,82	CASP9 - D2	24,89	24,93	0,03	24,91	7,09	-0,89	1,85	185,32

HEPG2(D3)	Ct		ORT		Ct		SD	ORT	Delta Ct	DDCt	2fold (2^-deltadelta c	% EXP
GAPDH - CONTROL	16,92	16,95	16,94	BAX - CONTROL	25,42	25,42	0,00	25,42	8,49			100,00
GAPDH - D3	17,51	17,23	17,37	BAX - D3	25,54	25,46	0,06	25,50	8,13	-0,36	1,28	127,90
GAPDH - CONTROL	16,92	16,95	16,94	P53 - CONTROL	24,76	24,47	0,21	24,62	7,68			100,00
GAPDH - D3	17,51	17,23	17,37	P53 - D3	22,24	22,27	0,02	22,26	4,89	-2,80	6,94	694,03
GAPDH - CONTROL	16,92	16,95	16,94	BCL 2 - CONTROL	26,61	26,93	0,23	26,77	9,84			100,00
GAPDH - D3	17,51	17,23	17,37	BCL 2 - D3	27,55	27,9	0,25	27,73	10,36	0,52	0,70	69,74
GAPDH - CONTROL	16,92	16,95	16,94	CASP3 - CONTROL	21,68	21,94	0,18	21,81	4,88			100,00
GAPDH - D3	17,51	17,23	17,37	CASP3 - D3	20,88	20,93	0,04	20,91	3,54	-1,34	2,53	253,15
GAPDH - CONTROL	16,92	16,95	16,94	CASP8 - CONTROL	26,19	26,39	0,14	26,29	9,36			100,00
GAPDH - D3	17,51	17,23	17,37	CASP8 - D3	25,93	25,58	0,25	25,76	8,39	-0,97	1,96	195,88
GAPDH - CONTROL	16,92	16,95	16,94	CASP9 - CONTROL	24,89	24,94	0,04	24,92	7,98			100,00
GAPDH - D3	17,51	17,23	17,37	CASP9 - D3	22,94	22,78	0,11	22,86	5,49	-2,49	5,62	561,78

Table S2. qPCR results for Ishikawa cell line

Ishikawa(D1)	Ct		ORT		Ct		SD	ORT	Delta Ct	DDCt	ld (2^-deltadelt	% EXP
GAPDH - CONTROL	15,09	15,04	15,07	BAX - CONTROL	25,29	25,07	0,16	25,18	10,12			100,00
GAPDH - D1	15,16	14,83	15,00	BAX - D1	24,16	24,25	0,06	24,21	9,21	-0,91	1,87	187,25
GAPDH - CONTROL	15,09	15,04	15,07	P53 - CONTROL	24,04	24,05	0,01	24,05	8,98			100,00
GAPDH - D1	15,16	14,83	15,00	P53 - D1	22,58	22,21	0,26	22,40	7,40	-1,58	2,99	298,97
GAPDH - CONTROL	15,09	15,04	15,07	BCL 2 - CONTROL	27,94	27,44	0,35	27,69	12,63			100,00
GAPDH - D1	15,16	14,83	15,00	BCL 2 - D1	28,78	28,81	0,02	28,80	13,80	1,18	0,44	44,29
GAPDH - CONTROL	15,09	15,04	15,07	CASP3 - CONTROL	22,92	22,88	0,03	22,90	7,84			100,00
GAPDH - D1	15,16	14,83	15,00	CASP3 - D1	21,93	21,59	0,24	21,76	6,77	-1,07	2,10	209,94
GAPDH - CONTROL	15,09	15,04	15,07	CASP8 - CONTROL	28,08	29,12	0,74	28,60	13,54			100,00
GAPDH - D1	15,16	14,83	15,00	CASP8 - D1	27,81	27,93	0,08	27,87	12,88	-0,66	1,58	158,01
GAPDH - CONTROL	15,09	15,04	15,07	CASP9 - CONTROL	25,06	25,86	0,57	25,46	10,40			100,00
GAPDH - D1	15,16	14,83	15,00	CASP9 - D1	24,49	24,36	0,09	24,43	9,43	-0,97	1,95	195,21

Ishikawa(D2)	Ct		ORT		Ct		SD	ORT	Delta Ct	DDCt	ld (2^-deltadelt	% EXP
GAPDH - CONTROL	15,09	15,04	15,07	BAX - CONTROL	25,29	25,07	0,16	25,18	10,12			100,00
GAPDH - D2	16	15,78	15,89	BAX - D2	24,17	23,95	0,16	24,06	8,17	-1,95	3,85	385,04
GAPDH - CONTROL	15,09	15,04	15,07	P53 - CONTROL	24,04	24,05	0,01	24,05	8,98			100,00
GAPDH - D2	16	15,78	15,89	P53 - D2	22,93	22,65	0,20	22,79	6,90	-2,08	4,23	422,81
GAPDH - CONTROL	15,09	15,04	15,07	BCL 2 - CONTROL	27,94	27,44	0,35	27,69	12,63			100,00
GAPDH - D2	16	15,78	15,89	BCL 2 - D2	28,12	28,15	0,02	28,14	12,25	-0,38	1,30	130,13
GAPDH - CONTROL	15,09	15,04	15,07	CASP3 - CONTROL	22,92	22,88	0,03	22,90	7,84			100,00
GAPDH - D2	16	15,78	15,89	CASP3 - D2	22,79	21,93	0,61	22,36	6,47	-1,37	2,58	257,58
GAPDH - CONTROL	15,09	15,04	15,07	CASP8 - CONTROL	28,08	29,12	0,74	28,60	13,54			100,00
GAPDH - D2	16	15,78	15,89	CASP8 - D2	27,35	27,66	0,22	27,51	11,62	-1,92	3,78	378,42
GAPDH - CONTROL	15,09	15,04	15,07	CASP9 - CONTROL	25,06	25,86	0,57	25,46	10,40			100,00
GAPDH - D2	16	15,78	15,89	CASP9 - D2	25,25	25,35	0,07	25,30	9,41	-0,99	1,98	197,93

Table S3. qPCR results for MDA-MB-231 cell line

**MDA-MB-231(D1)**	Ct		ORT		Ct		SD	ORT	Delta Ct	DDCt	ld (2^-deltadelt	% EXP
**GAPDH - CONTROL**	16,78	16,59	16,69	**BAX - CONTROL**	26,35	26,26	0,06	26,31	9,62			100,00
**GAPDH - D1**	16,95	16,73	16,84	**BAX - D1**	25,11	25,21	0,07	25,16	8,32	-1,30	2,46	246,23
**GAPDH - CONTROL**	16,78	16,59	16,69	**P53 - CONTROL**	22,71	22,5	0,15	22,61	5,92			100,00
**GAPDH - D1**	16,95	16,73	16,84	**P53 - D1**	21,09	22,02	0,66	21,56	4,72	-1,21	2,31	230,54
**GAPDH - CONTROL**	16,78	16,59	16,69	**BCL 2 - CONTROL**	27,9	28,31	0,29	28,11	11,42			100,00
**GAPDH - D1**	16,95	16,73	16,84	**BCL 2 - D1**	28,72	28,88	0,11	28,80	11,96	0,54	0,69	68,78
**GAPDH - CONTROL**	16,78	16,59	16,69	**CASP3 - CONTROL**	24,19	24,49	0,21	24,34	7,66			100,00
**GAPDH - D1**	16,95	16,73	16,84	**CASP3 - D1**	24,95	23,28	1,18	24,12	7,28	-0,38	1,30	130,13
**GAPDH - CONTROL**	16,78	16,59	16,69	**CASP8 - CONTROL**	28,58	28,55	0,02	28,57	11,88			100,00
**GAPDH - D1**	16,95	16,73	16,84	**CASP8 - D1**	27,98	28,02	0,03	28,00	11,16	-0,72	1,65	164,72
**GAPDH - CONTROL**	16,78	16,59	16,69	**CASP9 - CONTROL**	27,85	27,4	0,32	27,63	10,94			100,00
**GAPDH - D1**	16,95	16,73	16,84	**CASP9 - D1**	26,81	26,71	0,07	26,76	9,92	-1,02	2,03	202,79

**MDA-MB-231(D2)**	Ct		ORT		Ct		SD	ORT	Delta Ct	DDCt	ld (2^-deltadelt	% EXP
**GAPDH - CONTROL**	16,78	16,59	16,69	**BAX - CONTROL**	26,35	26,26	0,06	26,31	9,62			100,00
**GAPDH - D2**	17,04	17	17,02	**BAX - D2**	24,18	24,13	0,04	24,16	7,14	-2,49	5,60	559,83
**GAPDH - CONTROL**	16,78	16,59	16,69	**P53 - CONTROL**	22,71	22,5	0,15	22,61	5,92			100,00
**GAPDH - D2**	17,04	17	17,02	**P53 - D2**	21,18	21,45	0,19	21,32	4,30	-1,63	3,08	308,44
**GAPDH - CONTROL**	16,78	16,59	16,69	**BCL 2 - CONTROL**	27,9	28,31	0,29	28,11	11,42			100,00
**GAPDH - D2**	17,04	17	17,02	**BCL 2 - D2**	28,03	28,03	0,00	28,03	11,01	-0,41	1,33	132,87
**GAPDH - CONTROL**	16,78	16,59	16,69	**CASP3 - CONTROL**	24,19	24,49	0,21	24,34	7,66			100,00
**GAPDH - D2**	17,04	17	17,02	**CASP3 - D2**	22,99	23,94	0,67	23,47	6,45	-1,21	2,31	231,34
**GAPDH - CONTROL**	16,78	16,59	16,69	**CASP8 - CONTROL**	28,58	28,55	0,02	28,57	11,88			100,00
**GAPDH - D2**	17,04	17	17,02	**CASP8 - D2**	27,75	27,6	0,11	27,68	10,66	-1,22	2,34	233,76
**GAPDH - CONTROL**	16,78	16,59	16,69	**CASP9 - CONTROL**	27,85	27,4	0,32	27,63	10,94			100,00
**GAPDH - D2**	17,04	17	17,02	**CASP9 - D2**	26,49	26,73	0,17	26,61	9,59	-1,35	2,55	254,91

**MDA-MB-231(D3)**	Ct		ORT		Ct		SD	ORT	Delta Ct	DDCt	ld (2^-deltadelt	% EXP
**GAPDH - CONTROL**	16,78	16,59	16,69	**BAX - CONTROL**	26,35	26,26	0,06	26,31	9,62			100,00
**GAPDH - D3**	17,16	16,98	17,07	**BAX - D3**	25,34	25,99	0,46	25,67	8,60	-1,03	2,03	203,50
**GAPDH - CONTROL**	16,78	16,59	16,69	**P53 - CONTROL**	22,71	22,5	0,15	22,61	5,92			100,00
**GAPDH - D3**	17,16	16,98	17,07	**P53 - D3**	21,94	22,15	0,15	22,05	4,98	-0,94	1,93	192,52
**GAPDH - CONTROL**	16,78	16,59	16,69	**BCL 2 - CONTROL**	27,9	28,31	0,29	28,11	11,42			100,00
**GAPDH - D3**	17,16	16,98	17,07	**BCL 2 - D3**	28,17	29,01	0,59	28,59	11,52	0,10	0,93	93,30
**GAPDH - CONTROL**	16,78	16,59	16,69	**CASP3 - CONTROL**	24,19	24,49	0,21	24,34	7,66			100,00
**GAPDH - D3**	17,16	16,98	17,07	**CASP3 - D3**	22,96	23,89	0,66	23,43	6,36	-1,30	2,46	246,23
**GAPDH - CONTROL**	16,78	16,59	16,69	**CASP8 - CONTROL**	28,58	28,55	0,02	28,57	11,88			100,00
**GAPDH - D3**	17,16	16,98	17,07	**CASP8 - D3**	27,84	27,72	0,08	27,78	10,71	-1,17	2,25	225,01
**GAPDH - CONTROL**	16,78	16,59	16,69	**CASP9 - CONTROL**	27,85	27,4	0,32	27,63	10,94			100,00
**GAPDH - D3**	17,16	16,98	17,07	**CASP9 - D3**	26,87	25,87	0,71	26,37	9,30	-1,64	3,12	311,67

Table S4. qPCR results for HT-29 cell line

Ht29(D1)	Ct		ORT		Ct		SD	ORT	Delta Ct	DDCt	old (2^-deltadelt	% EXP
GAPDH - CONTROL	16,95	16,95	16,95	BAX - CONTROL	24,41	24,63	0,16	24,52	7,57			100,00
GAPDH - D1	17,32	17,21	17,27	BAX - D1	24,17	24,96	0,56	24,57	7,30	-0,27	1,21	120,58
GAPDH - CONTROL	16,95	16,95	16,95	P53 - CONTROL	19,93	20,71	0,55	20,32	3,37			100,00
GAPDH - D1	17,32	17,21	17,27	P53 - D1	18,72	19,03	0,22	18,88	1,61	-1,76	3,39	338,70
GAPDH - CONTROL	16,95	16,95	16,95	BCL 2 - CONTROL	25,57	27,95	1,68	26,76	9,81			100,00
GAPDH - D1	17,32	17,21	17,27	BCL 2 - D1	27,34	27,18	0,11	27,26	10,00	0,18	0,88	87,96
GAPDH - CONTROL	16,95	16,95	16,95	CASP3 - CONTROL	21,31	21,97	0,47	21,64	4,69			100,00
GAPDH - D1	17,32	17,21	17,27	CASP3 - D1	21,24	21,24	0,00	21,24	3,98	-0,72	1,64	164,15
GAPDH - CONTROL	16,95	16,95	16,95	CASP8 - CONTROL	26,36	26,39	0,02	26,38	9,43			100,00
GAPDH - D1	17,32	17,21	17,27	CASP8 - D1	25,73	25,21	0,37	25,47	8,21	-1,22	2,33	232,95
GAPDH - CONTROL	16,95	16,95	16,95	CASP9 - CONTROL	23,08	23,34	0,18	23,21	6,26			100,00
GAPDH - D1	17,32	17,21	17,27	CASP9 - D1	23,41	22,97	0,31	23,19	5,93	-0,34	1,26	126,14

Ht29(D2)	Ct		ORT		Ct		SD	ORT	Delta Ct	DDCt	old (2^-deltadelt	% EXP
GAPDH - CONTROL	16,95	16,95	16,95	BAX - CONTROL	24,41	24,63	0,16	24,52	7,57			100,00
GAPDH - D2	17,63	17,84	17,74	BAX - D2	25,13	24,93	0,14	25,03	7,30	-0,27	1,21	121,00
GAPDH - CONTROL	16,95	16,95	16,95	P53 - CONTROL	19,93	20,71	0,55	20,32	3,37			100,00
GAPDH - D2	17,63	17,84	17,74	P53 - D2	19,71	19,53	0,13	19,62	1,89	-1,49	2,80	279,92
GAPDH - CONTROL	16,95	16,95	16,95	BCL 2 - CONTROL	25,57	27,95	1,68	26,76	9,81			100,00
GAPDH - D2	17,63	17,84	17,74	BCL 2 - D2	27,61	27,95	0,24	27,78	10,05	0,24	0,85	84,97
GAPDH - CONTROL	16,95	16,95	16,95	CASP3 - CONTROL	21,31	21,97	0,47	21,64	4,69			100,00
GAPDH - D2	17,63	17,84	17,74	CASP3 - D2	21,45	21,58	0,09	21,52	3,78	-0,91	1,88	187,90
GAPDH - CONTROL	16,95	16,95	16,95	CASP8 - CONTROL	26,36	26,39	0,02	26,38	9,43			100,00
GAPDH - D2	17,63	17,84	17,74	CASP8 - D2	26,63	26,6	0,02	26,62	8,88	-0,54	1,46	145,90
GAPDH - CONTROL	16,95	16,95	16,95	CASP9 - CONTROL	23,08	23,34	0,18	23,21	6,26			100,00
GAPDH - D2	17,63	17,84	17,74	CASP9 - D2	23,28	23,53	0,18	23,41	5,67	-0,59	1,51	150,52

Ht29(D3)	Ct		ORT		Ct		SD	ORT	Delta Ct	DDCt	old (2^-deltadelt	% EXP
GAPDH - CONTROL	16,95	16,95	16,95	BAX - CONTROL	24,41	24,63	0,16	24,52	7,57			100,00
GAPDH - D3	15,91	15,75	15,83	BAX - D3	22,17	23,5	0,94	22,84	7,01	-0,57	1,48	147,94
GAPDH - CONTROL	16,95	16,95	16,95	P53 - CONTROL	19,93	20,71	0,55	20,32	3,37			100,00
GAPDH - D3	15,91	15,75	15,83	P53 - D3	17,91	17,65	0,18	17,78	1,95	-1,42	2,68	267,59
GAPDH - CONTROL	16,95	16,95	16,95	BCL 2 - CONTROL	25,57	27,95	1,68	26,76	9,81			100,00
GAPDH - D3	15,91	15,75	15,83	BCL 2 - D3	27,39	26,96	0,30	27,18	11,35	1,54	0,35	34,51
GAPDH - CONTROL	16,95	16,95	16,95	CASP3 - CONTROL	21,31	21,97	0,47	21,64	4,69			100,00
GAPDH - D3	15,91	15,75	15,83	CASP3 - D3	19,09	20,13	0,74	19,61	3,78	-0,91	1,88	187,90
GAPDH - CONTROL	16,95	16,95	16,95	CASP8 - CONTROL	26,36	26,39	0,02	26,38	9,43			100,00
GAPDH - D3	15,91	15,75	15,83	CASP8 - D3	24,88	24,76	0,08	24,82	8,99	-0,44	1,35	135,19
GAPDH - CONTROL	16,95	16,95	16,95	CASP9 - CONTROL	23,08	23,34	0,18	23,21	6,26			100,00
GAPDH - D3	15,91	15,75	15,83	CASP9 - D3	22,02	21,02	0,71	21,52	5,69	-0,57	1,48	148,45

Table S5. Table S5. qPCR results for Panc-1 cell line

PANC-1(D1)	Ct		ORT		Ct		SD	ORT	Delta Ct	DDCt	D2fold (2^-deltadelta ct)	% EXP
GAPDH - CONTROL	18,17	18,09	18,13	BAX - CONTROL	25,79	26,01	0,16	25,90	7,77			100,00
GAPDH - D1	18,79	18,71	18,75	BAX - D1	25,83	25,8	0,02	25,82	7,07	-0,70	1,63	163,01
GAPDH - CONTROL	18,17	18,09	18,13	P53 - CONTROL	22,91	21,98	0,66	22,45	4,32			100,00
GAPDH - D1	18,79	18,71	18,75	P53 - D1	21,97	21,08	0,63	21,53	2,78	-1,54	2,91	290,79
GAPDH - CONTROL	18,17	18,09	18,13	BCL 2 - CONTROL	25,5	25,89	0,28	25,70	7,57			100,00
GAPDH - D1	18,79	18,71	18,75	BCL 2 - D1	26,98	27,66	0,48	27,32	8,57	1,01	0,50	49,83
GAPDH - CONTROL	18,17	18,09	18,13	CASP3 - CONTROL	27,52	27,72	0,14	27,62	9,49			100,00
GAPDH - D1	18,79	18,71	18,75	CASP3 - D1	26,21	27,89	1,19	27,05	8,30	-1,19	2,28	228,15
GAPDH - CONTROL	18,17	18,09	18,13	CASP8 - CONTROL	30,32	30,79	0,33	30,56	12,43			100,00
GAPDH - D1	18,79	18,71	18,75	CASP8 - D1	30,12	30,92	0,57	30,52	11,77	-0,65	1,57	157,46
GAPDH - CONTROL	18,17	18,09	18,13	CASP9 - CONTROL	25,46	25,93	0,33	25,70	7,57			100,00
GAPDH - D1	18,79	18,71	18,75	CASP9 - D1	25,97	25,87	0,07	25,92	7,17	-0,39	1,31	131,49

PANC-1(D2)	Ct		ORT		Ct		SD	ORT	Delta Ct	DDCt	D2fold (2^-deltadelta ct)	% EXP
GAPDH - CONTROL	18,17	18,09	18,13	BAX - CONTROL	25,79	26,01	0,16	25,90	7,77			100,00
GAPDH - D2	18,44	18,23	18,34	BAX - D2	25,71	25,66	0,04	25,69	7,35	-0,42	1,34	133,79
GAPDH - CONTROL	18,17	18,09	18,13	P53 - CONTROL	22,91	22,8	0,08	22,86	4,73			100,00
GAPDH - D2	18,44	18,23	18,34	P53 - D2	22,35	22,19	0,11	22,27	3,94	-0,79	1,73	172,91
GAPDH - CONTROL	18,17	18,09	18,13	BCL 2 - CONTROL	25,5	25,89	0,28	25,70	7,57			100,00
GAPDH - D2	18,44	18,23	18,34	BCL 2 - D2	26,98	27,66	0,48	27,32	8,99	1,42	0,37	37,37
GAPDH - CONTROL	18,17	18,09	18,13	CASP3 - CONTROL	27,52	27,72	0,14	27,62	9,49			100,00
GAPDH - D2	18,44	18,23	18,34	CASP3 - D2	27,21	26,12	0,77	26,67	8,33	-1,16	2,23	223,46
GAPDH - CONTROL	18,17	18,09	18,13	CASP8 - CONTROL	30,32	30,79	0,33	30,56	12,43			100,00
GAPDH - D2	18,44	18,23	18,34	CASP8 - D2	29,95	30,19	0,17	30,07	11,74	-0,69	1,61	161,33
GAPDH - CONTROL	18,17	18,09	18,13	CASP9 - CONTROL	25,46	25,93	0,33	25,70	7,57			100,00
GAPDH - D2	18,44	18,23	18,34	CASP9 - D2	25,16	24,37	0,56	24,77	6,43	-1,14	2,20	219,62

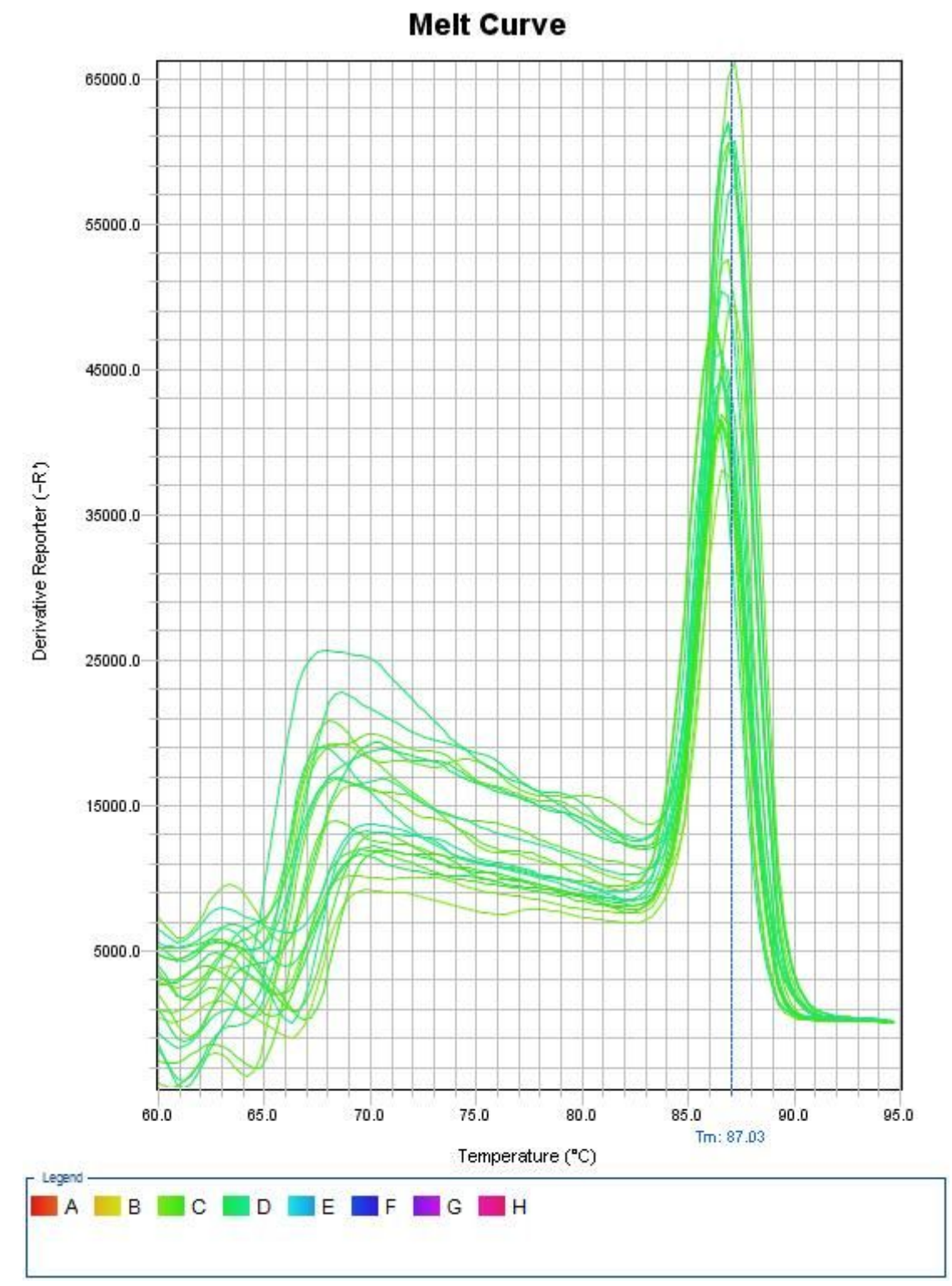

Figure S45. Melt curve of BAX.

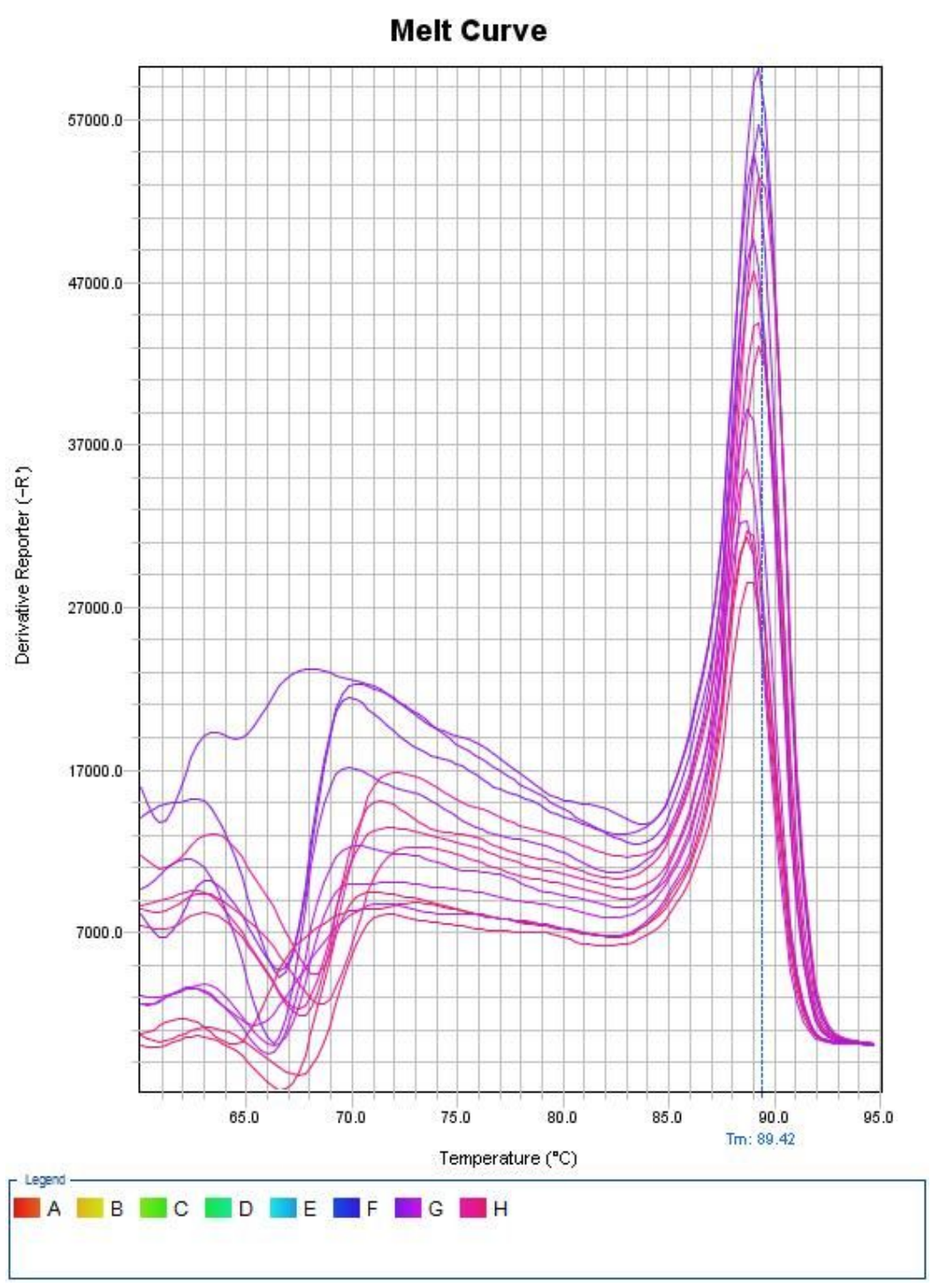

Figure S46. Melt curve of P53.

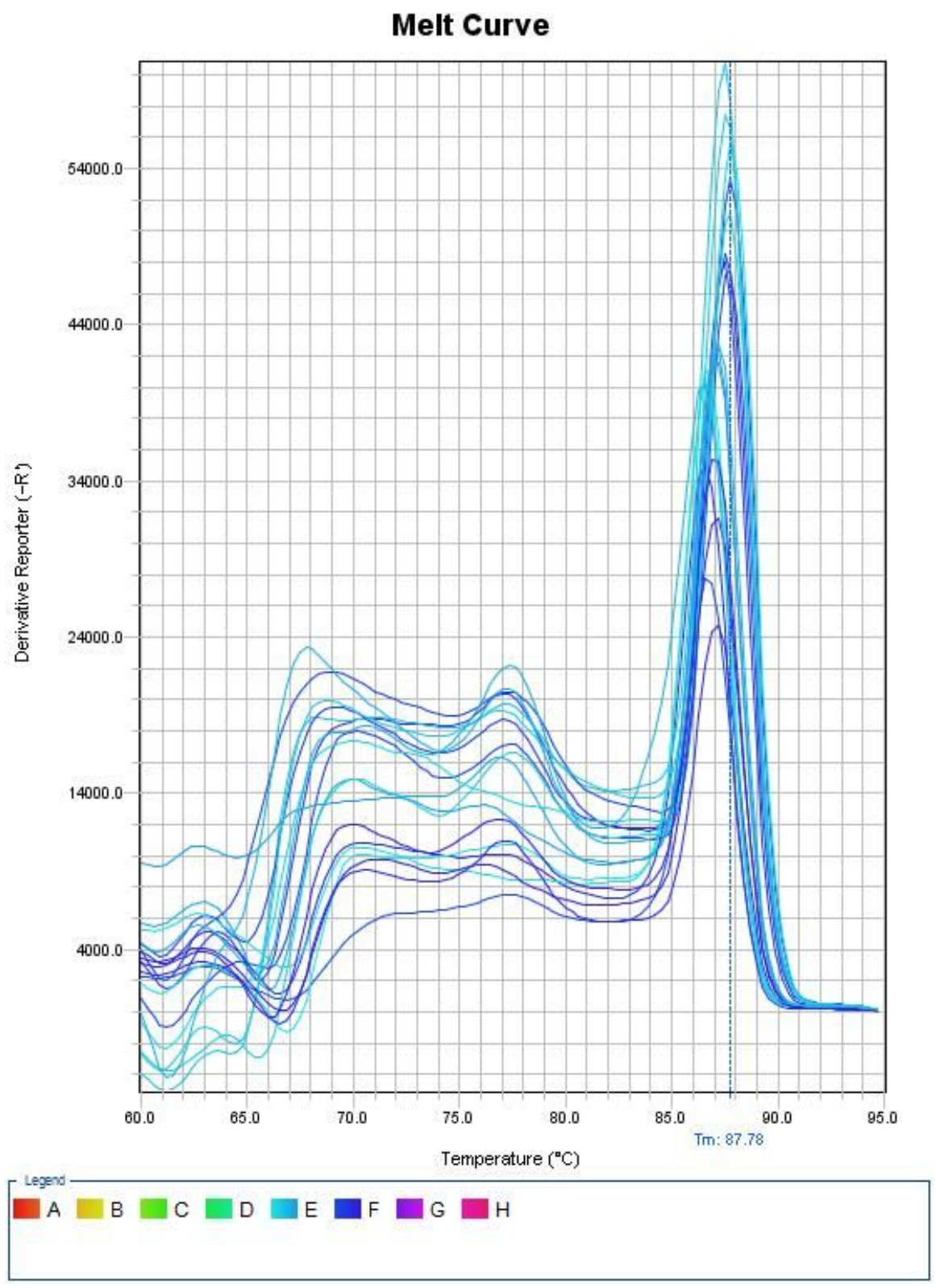

Figure S47. Melt curve of Bcl-2.

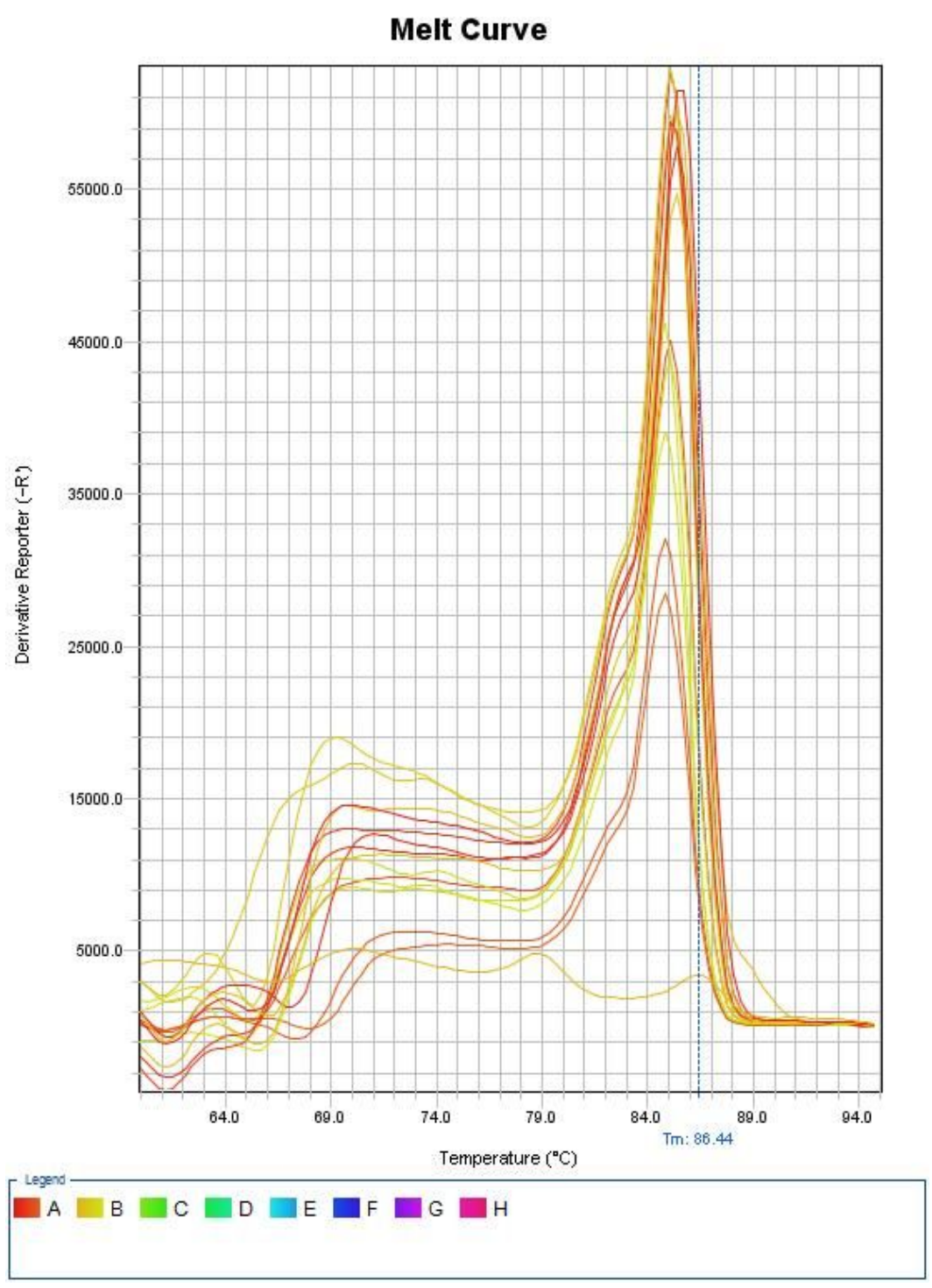

Figure S48. Melt curve of Caspase-3.

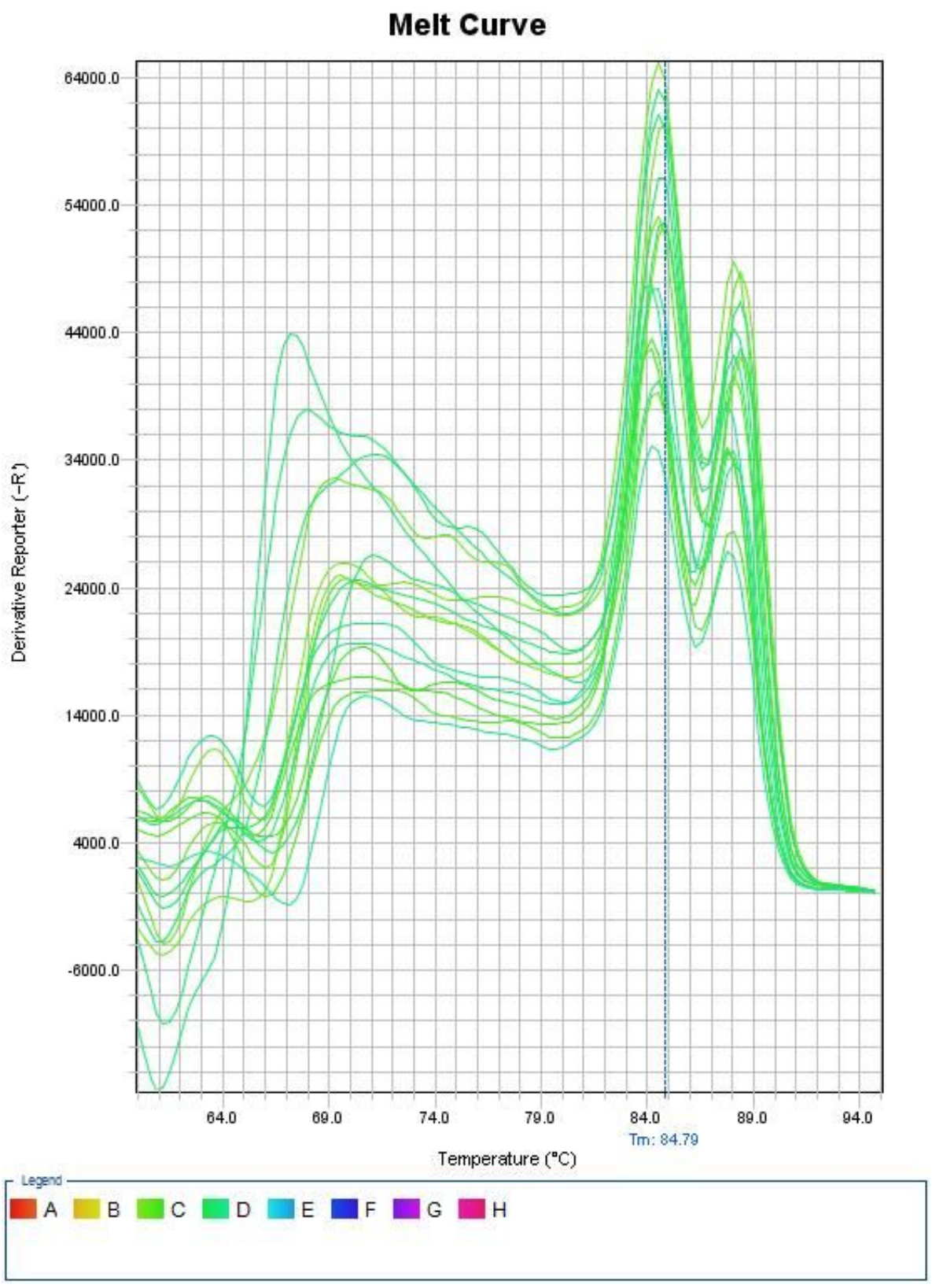

Figure S49. Melt curve of Caspase-8.

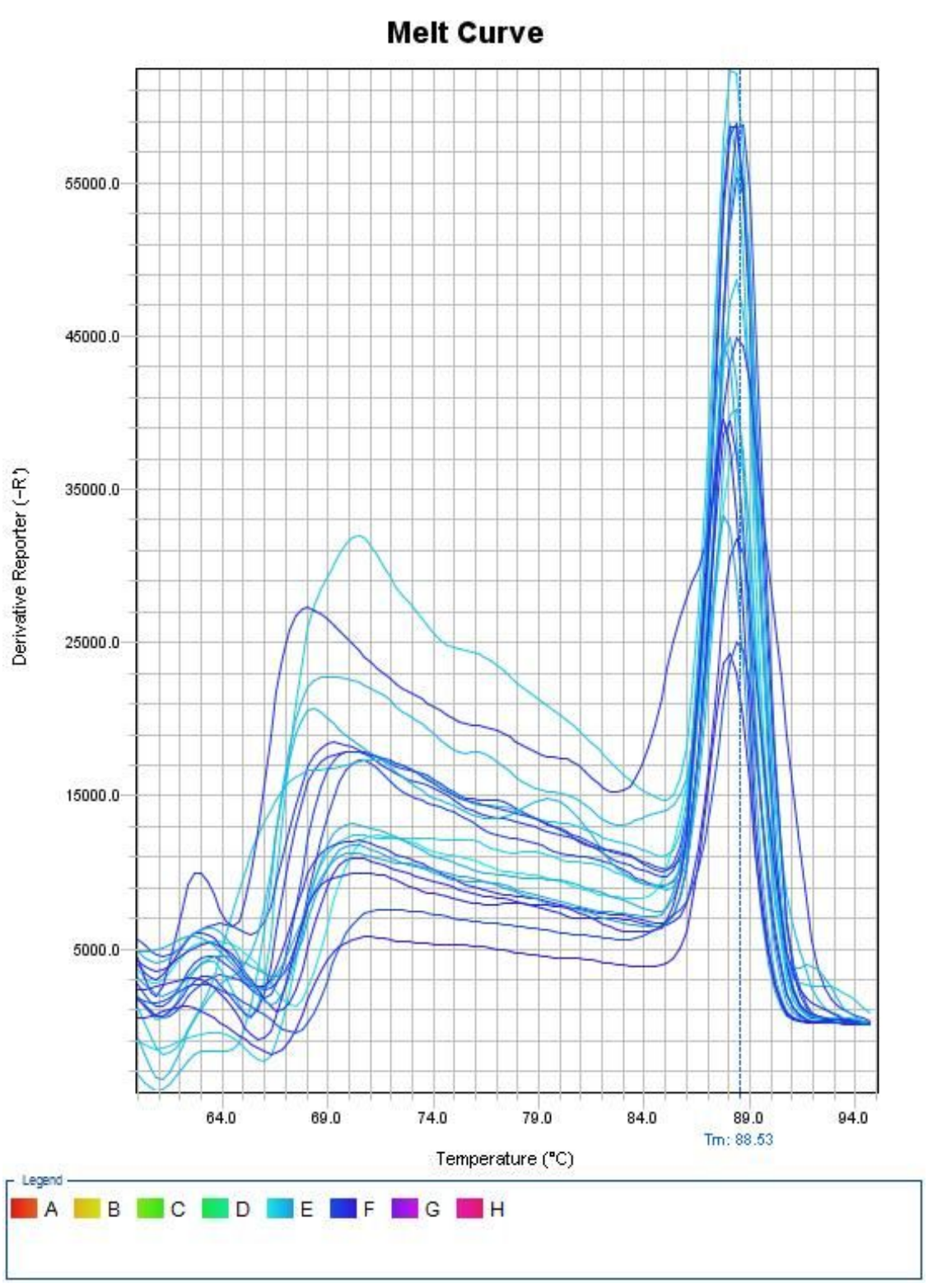

Figure S50. Melt curve of Caspase-9.

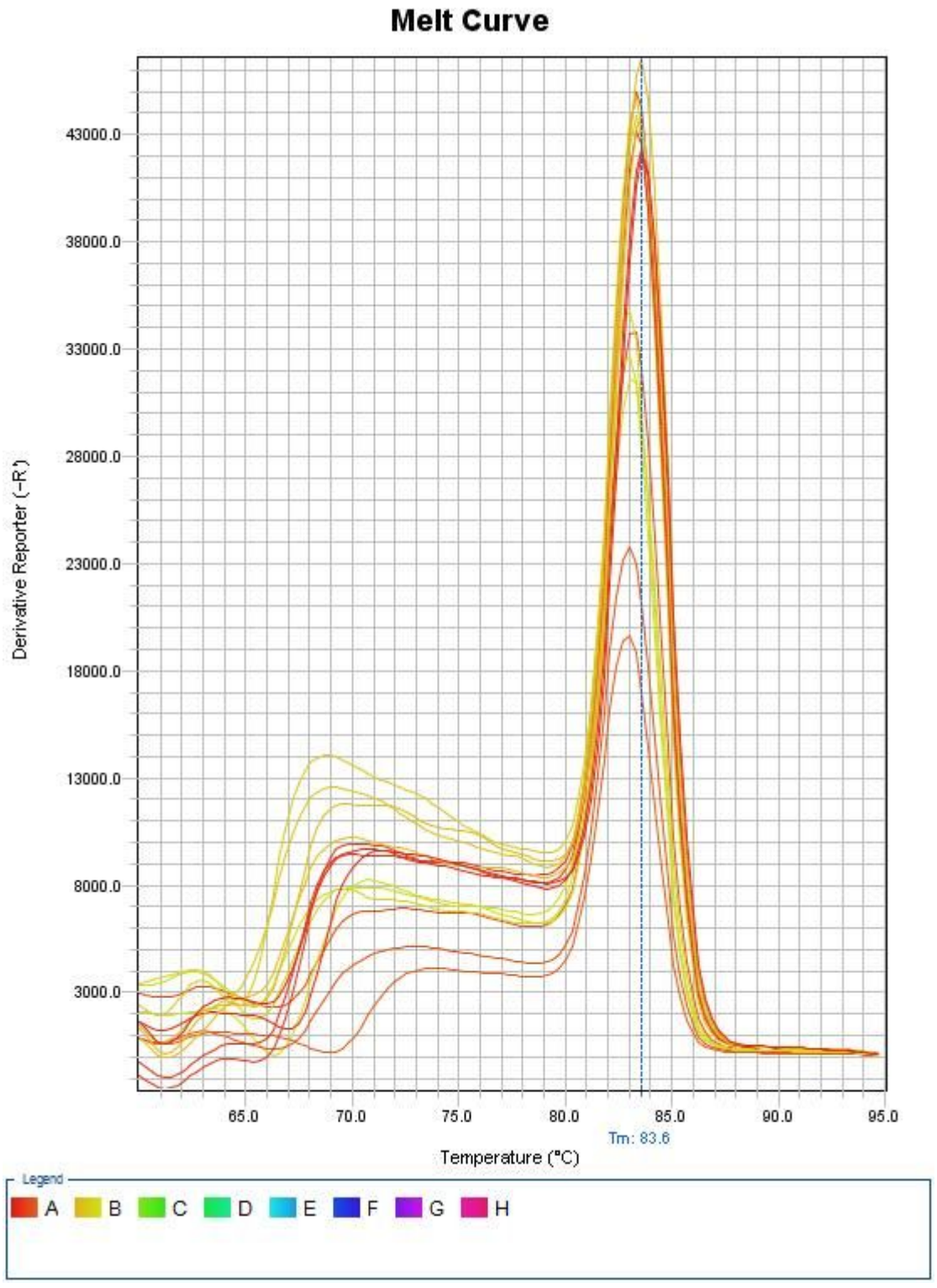

Figure S51. Melt curve of GAPDH.
